# Methylation risk scores are associated with a collection of phenotypes within electronic health record systems

**DOI:** 10.1038/s41525-022-00320-1

**Published:** 2022-08-25

**Authors:** Mike Thompson, Brian L. Hill, Nadav Rakocz, Jeffrey N. Chiang, Daniel Geschwind, Sriram Sankararaman, Ira Hofer, Maxime Cannesson, Noah Zaitlen, Eran Halperin

**Affiliations:** 1 Department of Computer Science, University of California Los Angeles, Los Angeles, CA USA; 2 Department of Computational Medicine, University of California Los Angeles, Los Angeles, CA USA; 3 Institute of Precision Health, University of California Los Angeles, Los Angeles, CA USA; 4 Department of Human Genetics, University of California Los Angeles, Los Angeles, CA USA; 5 Department of Anesthesiology and Perioperative Medicine, University of California Los Angeles, Los Angeles, CA USA; 6 Department of Neurology, University of California Los Angeles, Los Angeles, CA USA

**Keywords:** DNA methylation, Epigenomics, Genetic testing, Diagnostic markers, Personalized medicine

## Abstract

Inference of clinical phenotypes is a fundamental task in precision medicine, and has therefore been heavily investigated in recent years in the context of electronic health records (EHR) using a large arsenal of machine learning techniques, as well as in the context of genetics using polygenic risk scores (PRS). In this work, we considered the epigenetic analog of PRS, methylation risk scores (MRS), a linear combination of methylation states. We measured methylation across a large cohort (*n* = 831) of diverse samples in the UCLA Health biobank, for which both genetic and complete EHR data are available. We constructed MRS for 607 phenotypes spanning diagnoses, clinical lab tests, and medication prescriptions. When added to a baseline set of predictive features, MRS significantly improved the imputation of 139 outcomes, whereas the PRS improved only 22 (median improvement for methylation 10.74%, 141.52%, and 15.46% in medications, labs, and diagnosis codes, respectively, whereas genotypes only improved the labs at a median increase of 18.42%). We added significant MRS to state-of-the-art EHR imputation methods that leverage the entire set of medical records, and found that including MRS as a medical feature in the algorithm significantly improves EHR imputation in 37% of lab tests examined (median *R*^2^ increase 47.6%). Finally, we replicated several MRS in multiple external studies of methylation (minimum *p*-value of 2.72 × 10^−7^) and replicated 22 of 30 tested MRS internally in two separate cohorts of different ethnicity. Our publicly available results and weights show promise for methylation risk scores as clinical and scientific tools.

## Introduction

Widespread adoption of electronic health record systems coupled with an increasing interest in hospital biobanking systems has spurred research efforts spanning machine learning and genomics communities^1–7^. These efforts have produced increasingly accurate imputation (current state) and prediction (future state) of patient phenotypes from medical records^8,9^ and polygenic risk scores^1–3,10–14^, and are already being investigated in translational contexts^15–18^. For example, recent work has shown that machine learning can leverage high-dimensional data to aid in the prediction of a multitude of clinical phenotypes including cardiac function and arrhythmia^19–21^, post-operative complications^8,9^, sepsis^22^, breast cancer^11,23^, and prostate cancer^24^. Nonetheless, a genetics-based predictor such as the polygenic risk score may be limited in predictive utility as it does not account for changes in disease risk—for example, due to age, or changes in environment—throughout one’s lifespan^13^.

In this work, we examine the potential for epigenetic information to improve phenotype inference in combined biobank-EHR systems. As DNA methylation, henceforth referred to as simply “methylation”, is affected by both genetics and environment—such as lifestyle choices, diet, exercise, and smoking status—it captures multi-factorial information about predispositions to clinical conditions^25–31^. Moreover, methylation is readily available for use in existing biobanks that collect DNA samples, and recent advancements in methylation profiling technologies have enabled an abundance of large-scale studies of methylation and its role as a biomarker for a variety of phenotypes and health-related outcomes^25,31–37^. It is therefore a natural candidate for an extension of PRS, and we hypothesized that methylation can be used to complement genetics as a clinical prediction tool. To that end, we have generated and evaluated methylation risk scores (MRS), which are linear combinations of CpG methylation states^25^.

To comprehensively investigate the utility of MRS and characterize its properties, we conducted a study of 607 EHR-derived phenotypes spanning medications (e.g., vasopressers, glucocorticosteroids, uoroquinolones), labs (e.g., creatinine, glucose, prothrombin time), and diagnoses (e.g., T2D, bacterial pneumonia, anemia) that were available for a sufficient number of patients in the cohort. The cohort contained 831 patients—to the best of our knowledge, the largest epigenetic biobank dataset to date (including genetics, methylation, and EHR)—from the UCLA Health ATLAS cohort across a wide range of ages (18–90), racial and ethnic groups, and overall health (including patients ascertained on kidney and heart disease, with matched controls), with corresponding genetic and EHR data. This provides the opportunity to study the potential contribution of methylation to larger biobanks and in multiple clinical contexts. We find that the MRS-based imputations were more informative compared to PRS in 84 (92%) medications, 32 (94%) labs, and 123 (82%) diagnoses, more than doubling the imputation accuracy in over half of the outcomes considered. We also show that the MRS improves the imputation accuracy over PRS for cases in which the PRS is trained on very large external biobanks (roughly 3 orders of magnitude larger), as opposed to 831 samples that are available in this study. We observe that MRS improves over PRS learned from large biobanks in 40% of the tested phenotypes. Further, as our cohort was ethnically diverse, we performed replicability analyses within each racial and ethnic subset of our data. We broadly showed the replicability of the five best-imputed (by MRS) medications, labs, and diagnoses—46% and 100% of which replicated in (*n* = 118) non-white Hispanic-Latino- and (*n* = 543) white non-Hispanic-Latino-identifying individuals, respectively. Finally, we demonstrate the ability of MRS to transfer between methylation arrays and cohorts by conducting association studies of several MRS in multiple external EWAS datasets, where the minimum replication *p*-value 61 was 2.72 × 10^−7^.

These results provide evidence for the utility of methylation in phenotype imputation in general, and in biobank settings in particular. However, the promise of clinical translation of genomic risk scores, including PRS or MRS, is highly dependent on the clinical context of the patient. There is a large body of work investigating phenotype imputation and prediction in clinical settings using EHR data alone, typically with machine learning techniques, without any genomic data. To the best of our knowledge, the question of whether genomic data can be used to complement such algorithms has not been studied. Since the application of MRS or PRS to clinical data without taking into account the EHR data provides a limited clinical utility, this is a natural question.

Here, we demonstrate that MRS can be used in conjunction with EHR data to improve the imputation of clinical data of patients. Critically, most machine learning approaches rely on imputation because of the inability of such algorithms to process missing data, making accurate imputation a crucial step. We found that the combination of MRS with a gold standard imputation approach—SoftImpute^38^—for clinical data imputation, provides improved accuracy (*R*^2^) in 37.3% of the examined phenotypes with a median increase of 47.6%. This result provides the potential to improve machine learning algorithms that use the EHR data, by complementing the data with methylation information for the patients.

In summary, our results quantify the contribution of methylation information in clinical settings, both in isolation and in conjunction with the EHR data, and they demonstrate the potential utility of epigenetic biobanks in clinical settings.

## Results

### Risk model description

Analogous to the PRS^39,40^, we defined the MRS by a linear combination of *m* CpG site beta values *c* and weights *w*:

1 $${{{\rm{MRS}}}}=\mathop{\sum }\limits_{i=1}^{m}{w}_{i}{c}_{i}$$

To ensure the methylation risk score added predictive value over commonly captured features (e.g., age and sex), we created a baseline predictive model that included patients’ age, sex, reference-based methylation cell-type composition estimates^41^, self-reported race-ethnicity, self-reported smoking status, and the first ten genetic principal components^27^ (see Supplementary Table 1 for cohort demographic data). We fit the baseline model using a linear or logistic regression model depending on whether the outcome was continuous or binary. We compared the baseline model to models that included the baseline features as well as either methylation or genotype data. For both the MRS and PRS, we used regression with LASSO, elastic net, and ridge regularization over the genomic features while treating the baseline features as fixed covariates. We fit all models using 10-fold double cross-validation, wherein each training set an additional cross-validation was performed for hyperparameter selection, then this training-set cross-validated model was used to predict the held-out test set. We tested for significance using an association test (via linear regression) between the cross-validated predicted outcome (i.e., the concatenated predictor across all folds) and the true outcome. For full details see “Methods”.

### Methylation risk scores significantly outperform the baseline and PRS models

From our EHR database, we extracted diagnosis codes, medication orders, and the most recent lab results, all of which occurred before the methylation samples were collected. We aggregated the ICD codes into higher-level phenotypes according to the phenotype code (Phecode) mapping proposed by Denny et al.^42,43^ and grouped individual medications by pharmaceutical subclass to increase generalizability and power.

We trained penalized linear models to predict clinical phenotypes for which there was a sufficient number of patient data available, which included 168 medication subclasses, 69 lab values, and 370 Phecodes. Using a Bonferroni-adjusted association test, the baseline and MRS models significantly imputed the usage of 69 and 88 medications, 18 and 33 labs, and 106 and 139 Phecodes, respectively (Supplementary Fig. 1). We compared the performance of the MRS to a model that used both the PRS and baseline features on the same set of individuals, which significantly imputed the usage of 53 medications, 20 lab results, and 93 Phecodes. Notably, the baseline model imputed a greater number of medications and Phecodes than models that leveraged a PRS, which suggests that including genomic features may either add noise or our sample size may not have been sufficient to discover their effects for certain outcomes. We also show in Supplementary Fig. 2 that the baseline model gains some of its predictive power from genomics-derived features like ancestry PCs or estimated cell counts, and therefore a PRS or MRS may not offer a substantial improvement over these features for certain outcomes under the current sample sizes.

**Fig. 1:**
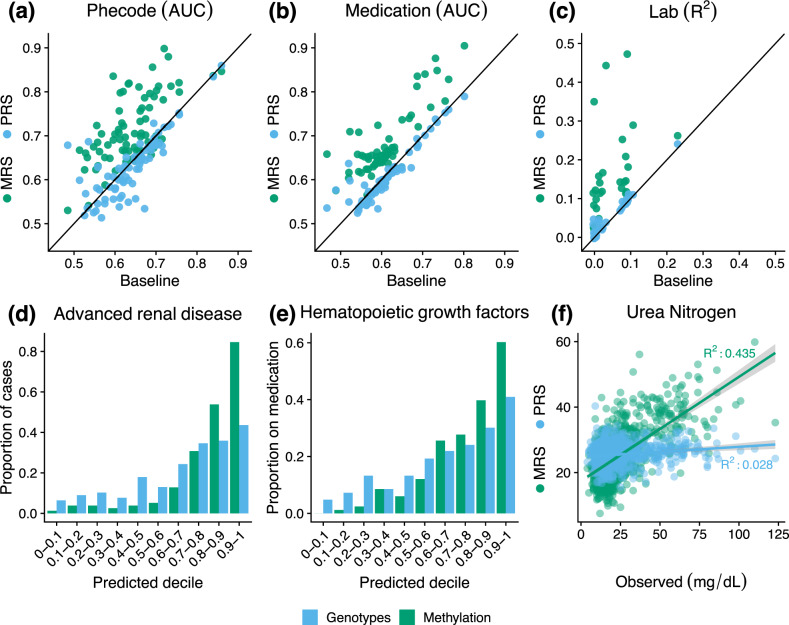
MRS increases imputation accuracy on a variety of outcomes. **a**–**c** The performance of the PRS (blue) and MRS (green) imputations on the y-axis with the baseline model performance on the x-axis. The performance of binary phenotypes (Phecodes (**a**), medications (**b**)) is measured using area under the ROC curve (AUC) and the performance of continuous phenotypes (lab results (**c**)) is measured using proportion of variance explained (*R*^2^). Shown is the performance on the union of outcomes that were significantly improved over the baseline model by either the MRS or PRS and that were significantly imputed by their corresponding predictor (72 Phecodes, 59 medications, and 31 labs). **d**–**f** The disease incidence as a function of the PRS (blue) and MRS (green) binned by deciles (**d**, **e**); and the observed Urea Nitrogen lab result value plotted against its imputed value (**f**).

Next, we investigated outcomes for which genomics-based predictors add predictive power to the baseline features and, in such cases, the extent to which their inclusion improves predictive accuracy. On the outcomes for which the genomics-based predictors produced statistically significant imputations, we conducted a likelihood ratio test comparing an association test of the true outcome using the cross-validated baseline predictor alone, to a model that included the cross-validated baseline predictor as well as the cross-validated predictor that included both baseline and genomic features (“Methods”). The methylation significantly improved the baseline predictor for 54 medications, 29 labs, and 56 Phecodes, and led to a median increase of 10.74%, 141.52%, and 15.46% over the baseline predictor’s accuracy (AUC, *R*^2^) in each outcome, respectively (Fig. 1). The genotypes significantly improved the baseline predictor for 8 medications, 3 labs, and 11 Phecodes, and led to a median increase of 18.42% over the baseline in the *R*^2^ of the labs, but a median decrease of 1.75% and 0.94% in AUC of the medications and Phecodes, respectively (Fig. 1). We note that our internal sample size is likely under-powered to discover small genetic effects and therefore suggest the contributions made by the genotypes may be due to additional ancestry signal that was not captured by the first few genetic PCs.

The medications that improved the greatest using methylation corresponded to drugs often prescribed to individuals with neutropenia (hematopoeitic growth factors, AUC baseline 0.706 95% CI [0.661,0.748] to AUC methylation 0.840 [0.807,0.871]) or chronic kidney disease (phosphate binder agents AUC from 0.731 [0.683,0.777] to 0.876 [0.842,0.907]). The lab panels best improved with the addition of the methylation-based predictor included those related to kidney function as well as cell counts (Urea nitrogen baseline adjusted *R*^2^ 0.032 [0.007,0.057] compared to 0.443 [0.377,0.509] with methylation, hemoglobin 0.107 [0.063,0.151] to 0.289 [0.232,0.346]). The addition of the genotype-based predictor improved the imputation of hematocrit (adjusted *R*^2^ from 0.077 [0.041,0.114] to 0.092 [0.052,0.132]) and total protein (adjusted *R*^2^ 0.094 [0.047,0.141] to 0.111 [0.060,0.162]), both of which are influenced by ancestry^44,45^. In the context of Phecodes, methylation greatly increased the imputation of advanced renal disease over the baseline and genotype models (for example, AUC baseline 0.720 [0.673,0.762] to 0.898 [0.867,0.927] with methylation), and the genotype model increased the imputation of actinic keratosis (AUC from 0.694 [0.631,0.747] to 0.728 [0.672,0.784]).

Overall, when looking at the intersection of medications significantly imputed by either the methylation and genotypes or methylation and baseline, 92% were better imputed by methylation sites than genotypes (median 9.13% increase) and 78% were better imputed by methylation compared to the baseline (median 6.81% increase). Methylation improved the baseline imputation accuracy by over 15% for 14 medications. In the context of significantly imputed lab values, methylation explained more variability than the baseline (median 398% increase) and genotype (median 274% increase) predictors in 97% and 94% of the respective union of significantly imputed labs. For 22 labs, the percent increase of imputation accuracy was greater than 15% over the baseline model. Methylation was more accurate than the baseline (median 3.48% increase) or genotypes (median 6.58% increase) for 70% and 83% of each respective union of Phecodes. For 29 Phecodes, the methylation offered over a 15% increase in predictive accuracy compared to the baseline model. For a substantial proportion of outcomes, the MRS predictor added statistically significant predictive value over the PRS predictor (Supplementary Fig. 3). This was generally not true when comparing whether the PRS added predictive value over the MRS. For the imputation performance on the full list of phenotypes, see Supplementary Tables 2, 3, and 4. To see the number of CpGs selected for each MRS, see Supplementary Tables 5, 6, and 7.

Importantly, cell-type composition, age, sex, BMI, smoking status, and ancestry provide sufficient power for the imputation of many EHR outcomes. We show explicitly in Supplementary Fig. 4 that genomics-derived features such as cell-type composition and ancestry PCs likely contribute to accurate imputation of several outcomes. In our analyses, we directly compared the power gained by methylation over the aforementioned set of baseline features. However, we note that obtaining these baseline features may be unnecessary as the methylation alone may capture their signal^27,30,32,46,47^. Further, previous reports have suggested that approaches that fit all methylation probes simultaneously with regularization may perform better when excluding latent confounders, such as cell-type composition^48^. We therefore suggest that using the methylation alone is sufficient to replicate a substantial proportion of the associations generated from the baseline features.

### Using methylation risk scores improves imputation approaches

Due to significant heterogeneity in patient populations, the diagnosis and treatment process can vary widely between patients, causing many variables to be left unobserved. This sparse structure in the data must be reconciled before performing many downstream analyses, and the imputation accuracy of these unobserved variables is therefore crucial to subsequent steps. A commonly-used approach for imputation is matrix completion, for example, SoftImpute^38^, where the data matrix is reconstructed from a low-rank representation. Often, one would jointly use demographic information, diagnosis codes, lab results, and medications to generate an estimate of the unobserved EHR values using an imputation method such as SoftImpute, and therefore we used this as our baseline imputation estimate^49^.

**Fig. 2:**
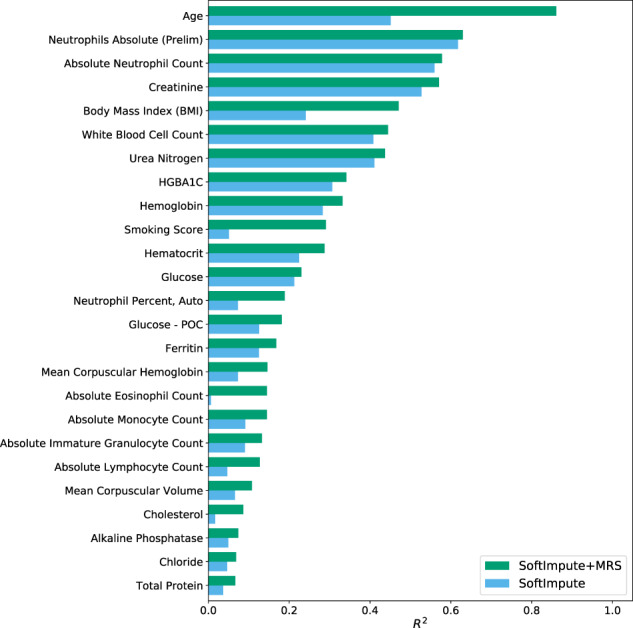
Improvement in lab result imputation performance by including MRS. For lab results that were significantly better imputed using a matrix completion imputation procedure that included the MRS values, we compare the quality of the imputed values (*R*^2^) using only the EHR data (SoftImpute) to the values generated when including the MRS values in addition to the EHR data (SoftImpute+MRS).

To investigate whether methylation can add additional useful information to the imputation, we included the MRS values as part of the imputation procedure and compared the performance to the estimates that do not take methylation data into account (see “Methods”). Specifically, we included cross-validated MRS values for diagnosis codes, lab results, medications, and demographics that were significantly imputed as 261 additional features (i.e., columns of the input matrix) in the imputation procedure. We randomly removed a subset of the observed lab results, as well as other labs that are ordered as part of the same lab panel(s), and imputed the masked values using the remaining observed values. The imputed values were then compared to the held-out, masked values to assess the quality of the imputation. In Fig. 2, we show the imputation accuracy (*R*^2^ between the masked true and imputed values) for labs where the addition of cross-validated MRS to the baseline SoftImpute procedure explained significantly more variability. Of the 67 lab results considered, 25 (37.3%) were significantly better imputed by including the MRS values. Including the MRS values led to a median increase of 47.6% (95% CI 17.3–90.9%) in the imputation *R*^2^ values.

#### Methylation risk scores will improve with larger sample sizes

In this study, our analyses of imputation accuracy were performed on 831 individuals’ methylation and genetic features. For many phenotypes, the genetic effects are relatively small and require large sample sizes to identify associations between genomic features and the outcome of interest. Consequently, in many biobanks the number of individuals with measured genomic features is several orders of magnitude larger than our sample size^1–3^. While the methylation data provided sufficient power to significantly impute numerous outcomes, there may remain much power to be gained by increasing the number of methylation samples to numbers approaching biobank-scale.

**Fig. 3:**
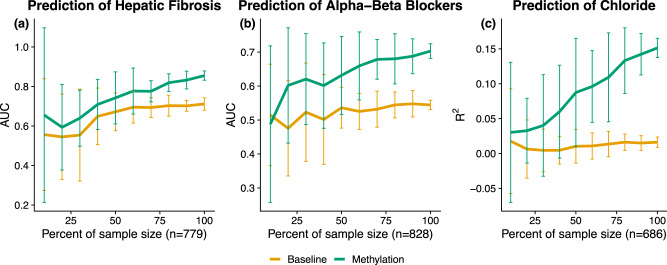
Imputation accuracy may improve with additional samples. We downsampled the number of individuals to evaluate the imputation performance as a function of sample size using a well-imputed Phecode (**a**), medication (**b**), and lab value (**c**). The performance is significantly affected by the number of individuals, suggesting that there is additional power to be gained with the addition of more methylation samples. Error bars indicate 95% confidence intervals.

To determine the role of sample size in our imputation accuracy, we performed an experiment in which we downsampled the number of individuals in our data and trained models on the subsampled data. From the set of outcomes most accurately imputed by methylation and that also significantly improved the baseline’s imputation, we chose 10 medications, labs, and Phecodes on which to perform 10-fold cross-validation. For each sample size, we repeated the procedure 20 times to attempt to mitigate variance due to ascertainment effect. Though we selected features that had high accuracy using the full set of data, our results suggest that our models may become more accurate as the sample size increases (Fig. 3; Supplementary Figs. 6, 7, 8). We further posit that there may be additional outcomes that will be significantly imputed as the number of methylation samples increases.

### Comparing MRS to UKBiobank PRS

**Fig. 4:**
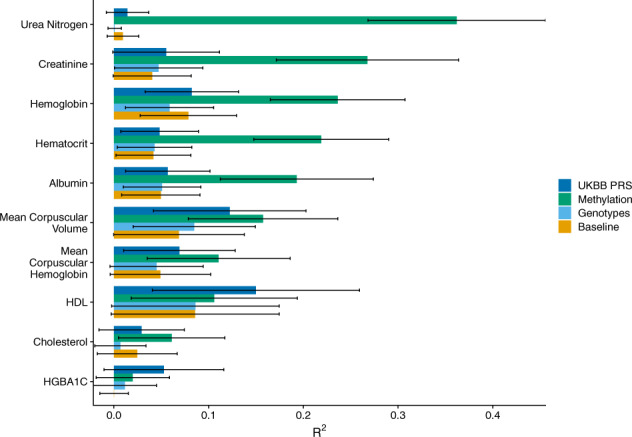
Labs as imputed by methylation, genotypes, and an externally-trained polygenic risk score. The cross-validated *R*^2^ between the true and imputed lab value on 541 unrelated patients of non-Hispanic-Latino white-identifying individuals using a baseline predictor as well as a baseline predictor with methylation, genotypes, and a PRS externally-trained from UKBiobank summary statistics. HDL corresponds to high-density lipoprotein cholesterol and HGBA1C to glycated hemoglobin. Error bars indicate 95% confidence intervals.

As expected, due to a small sample size and the likely small effects of SNPs on phenotypes, the PRS developed using the UCLA cohort did not add substantial predictive power over the baseline features. Studies leveraging biobanks with sample sizes several magnitudes larger than the cohort at UCLA however, have shown non-zero heritability for a variety of phenotypes^1,50–52^. Therefore, we sought to compare the MRS and PRS generated with the UCLA data to a polygenic risk score created using the UKBiobank data^1^. To do so, we obtained the genotype weights corresponding to 10 polygenic risk scores trained on the UKBiobank (Supplementary Table 10)^1,51–53^ data and imputed the external risk scores into our health record system using PLINK^54^. We included in the comparison labs that were significantly imputed by the baseline model and excluded labs that corresponded to cell counts or labs for which the internal PRS outperformed the external PRS (indicating a mismatch in the phenotypes or cryptic population structure that was unaccounted for by principal components). While the external polygenic risk score improved substantially the imputation performance relative to the internal polygenic risk score, it did not significantly outperform the methylation for any of the tested phenotypes (Fig. 4). The methylation remained the best predictor in general—even when trained on fewer than 1000 samples—significantly outperforming the other models in the imputation of urea nitrogen, creatinine, hemoglobin, hematocrit, and albumin. The externally-derived polygenic risk score greatly outperformed both the internally-derived PRS and the MRS when predicting glycated hemoglobin (HGBA1C) and HDL levels, however, the improvement was not significant. For detailed information on the external PRS and accession numbers, see Supplementary Table 10.

Similarly to the analyses in which we examined whether predictors that leverage genomics offered predictive value over the baseline predictor, we examined whether our internal MRS and the externally trained PRS offer information that is complementary to the other. To do so, we measured the accuracy when using the MRS, external PRS, both risk scores, and as well as both risk scores and their interaction on the same set of labs as our original analysis. None of the models significantly outperformed the MRS alone (Supplementary Fig. 10). However, there was a significant interaction effect between the MRS and external PRS on creatinine (*p* = 9.16e−05), as well as a nominally significant interaction effect on mean corpuscular hemoglobin (*p* = 1.45e−02). As the interaction terms improved the accuracy for both outcomes, there may be added value in leveraging both MRS and PRS for imputation tasks, especially those that take advantage of non-linear effects.

### Evaluation of methylation risk scores across ancestral populations

Previous reports have suggested that a significant confounder to the application and versatility of polygenic risk scores is population structure, where a population-specific bias is induced that affects generalizability of PRS to different ancestries^55–57^. The collection of samples analyzed throughout this study is ethnically heterogeneous—individuals were self-identified as non-Hispanic/Latino European, Hispanic/Latino, Black, or Asian. Methylation data is also influenced by differences in population^58^, and in particular the first several methylation principal components sufficiently capture population structure in European and African groups^59,60^). Consequently, we examined the performance of the methylation risk scores within and across ancestral populations.

**Fig. 5:**
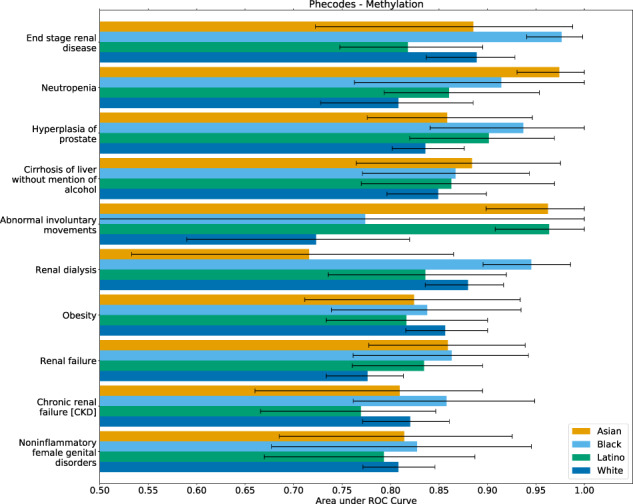
Best methylation-imputed Phecodes within ancestral populations. After training a model on the entire heterogeneous population of individuals, we evaluated the predictive performance within each population separately. We observed only 6 (of 60) significant differences between self-reported ancestral groupings. Error bars indicate 95% confidence intervals.

Primarily, after training the models on the entire heterogeneous set of samples, we examined the predictive performance within each ancestral population. When we examined the top 10 best-imputed (by MRS across the entire set of individuals) lab panels, medications, and Phecodes, only 10 of the entire 180 possible comparisons ($4 \choose 2$ comparisons across 30 outcomes) displayed significant differences between the predictive performance within each population separately (Fig. 5, Supplementary Figs. 11, 12).

In a second replication analysis we trained predictive models within ancestral groupings separately. As the individuals self-identified as either Black or Asian comprised <100 individuals in both groupings, we focused our analyses on Hispanic/Latino- and white-non-Hispanic/Latino-identifying individuals. We re-trained models for the top 5 best-imputed (by MRS) medications, lab panels, and unique Phecodes on the Hispanic/Latino individuals and white non-Hispanic/Latino individuals alone and treated a prediction as significant if its association p-value was lower than 0.01. Creatinine, hemoglobin, and urea nitrogen replicated across both groupings, however, hematocrit and mean corpuscular hemoglobin did not replicate in the Latino/Hispanic grouping (Table 1). In the context of medications, CMV agents, osmotic diuretics, phosphate binder agents, hematopoietic growth factors, and immunosuppressive agents replicated within the white non-Hispanic/Latino population but only CMV and immunosuppressive agents replicated within the Hispanic/Latino population (Table 1). Finally, Phecodes corresponding to immunity deficiency, hypertensive renal disease, and end-stage renal failure replicated within both groupings, however, neutropenia and anemia replicated only within the white non-Hispanic/Latino set of individuals (Table 1).

**Table 1:** Replication statistics within ethnic groupings.

Outcome	Metric	Accuracy, *p*-value Hispanic/Latino (*n* = 118)	Accuracy, *p*-value white, non-Hispanic/Latino (*n* = 543)	Accuracy, *p*-value all ethnicities (*n* = 833)
Creatinine	*R*^2^	0.217, 4.63e−07	0.356, 7.47e−46	0.457, 1.27e−95
Hematocrit	*R*^2^	0.045, 2.91e−02	0.188, 1.87e−21	0.246, 1.14e−42
Hemoglobin	*R*^2^	0.096, 1.21e−03	0.204, 2.54e−23	0.283, 3.02e−50
Mean corpuscular hemoglobin	*R*^2^	0.050, 2.12e−02	0.122, 9.70e−14	0.208, 7.04e−35
Urea nitrogen	*R*^2^	0.289, 2.97e−09	0.349, 7.61e−44	0.435, 2.50e−87
CMV Agents	AUC	0.874, 9.27e−07	0.875, 3.47e−16	0.905, 1.72e−38
Osmotic Diuretics	AUC	0.530, 0.841	0.842, 2.27e−12	848, 6.37e−34
Phosphate binder agents	AUC	0.608, 0.321	0.819, 7.76e−17	0.876, 1.11e−50
Hematopoietic growth factors	AUC	0.567, 0.476	0.780, 1.51e−19	0.840, 1.75e−45
Immunosuppressive agents	AUC	0.721, 1.43e−04	0.823, 6.36e−22	0.828, 9.44e−41
Neutropenia	AUC	0.689, 5.60e−02	0.800, 7.68e−10	0.836, 1.11e−19
Immunity deficiency	AUC	0.889, 4.06e−09	0.818, 3.26e−19	0.821, 9.74e−33
Anemia	AUC	0.637, 9.75e−02	0.698, 3.13e−08	0.789, 1.40e−32
Hypertensive renal disease	AUC	0.715, 1.35e−04	0.688, 6.74e−10	0.801,1.45e−42
End-stage renal failure	AUC	0.677, 1.80e−03	0.868, 2.51e−29	0.898, 5.46e−72

Predictive accuracy (*R*^2^ and AUC) for MRS trained within only Latino/Hispanic- or white-non-Latino/Hispanic-identifying individuals compared to the accuracy trained on the entire, cross-ethnic cohort.

### Replication of methylation risk scores across external datasets

To evaluate the transferability of the MRS to a different population, we performed several experiments in which we imputed the MRS into external datasets. Primarily, we focused on imputation of kidney-related outcomes as they were the most accurately imputed in our own cohort. To do so, we leveraged a dataset that used the HumanMethylation27k array to measure the methylation of 194 individuals who had Type 1 Diabetes, 49.7% of whom had nephropathy (cases)^61^. We re-trained the models for a Phecode corresponding to chronic renal disease as well as labs corresponding to creatinine and urea nitrogen on our in-house data, limiting our analysis to the 27,000 sites that belonged to the external dataset. The imputed chronic renal disease was significantly associated with nephropathy in the external dataset (*p* = 8.32e−05, AUC = 0.684 [0.615,0.758]. Further, both of the imputed values for creatinine and urea nitrogen were significantly associated with nephropathy (*p* = 5.11e−07, AUC=0.739 [0.670,0.808] and *p* = 3.71e−05, AUC = 0.693 [0.619,0.767], respectively). Importantly, when limiting our internal analysis to sites only on the 27k array, the association signal decreased (for chronic renal disease from *p* = 6.81e−51 to *p* = 3.13e−29, creatinine *p* = 1.27e−95 to *p* = 3.14e−62, and urea nitrogen *p* = 2.50e−87 to *p* = 8.44e−34). However, likely due to correlation between CpGs, the association tests for outcomes trained on the smaller set of sites were still significant.

Second, we expanded our replication analyses to include phenotypes that were unrelated to kidney function. In these analyses, we revisited epigenome-wide association studies (EWAS) of Schizophrenia^62^ and Rheumatoid Arthritis^63^ and imputed commonly prescribed medications for each dataset—for Schizophrenia we used phenothiazines, and for Rheumatoid Arthritis we used glucocorticosteroids. To ensure our MRS captured medication intake status and were not merely serving as proxies for the disease, we re-trained our models while conditioning on the trait of interest. The imputed phenothiazine intake was significantly associated with Schizophrenia case-control status (*p* = 8.71e−04, AUC = 0.568 [0.527,0.611]) and the imputed glucocorticosteroids usage was significantly associated with Rheumatoid Arthritis case-control status (*p* = 2.72e−07, AUC = 0.626 [0.584,0.669]. Weights for both medications were trained on CpGs corresponding to those present on the HumanMethylation450k array and also included their corresponding disease in the baseline set of covariates. Accordingly, the association signal of phenothiazines dropped from 1.14e−07 to 3.99e−05 and the performance of glucocorticoids dropped from 1.35e−16 to 1.82e−15 when compared to the MRS trained on the set of EPIC array CpGs and with the baseline features as covariates.

## Discussion

In this study, we provide a comprehensive investigation of the utility of methylation risk scores in a clinical setting. We used (to our knowledge) the largest methylation biobank cohort produced to date, which includes methylation, genotype, and comprehensive EHR data for all patients. We find that the MRS improved imputation performance over a baseline model by 10.65%, 156.31%, and 14.59% when predicting medication usage, lab panel values, and diagnosis codes, respectively. These contributions are significantly more substantial than those obtained by PRS.

The vision of genomic biobanks is that the genomic data will be translated into improved clinical diagnoses and treatment decisions^12,13,64^. In practice, clinical decisions are not expected to be based solely on genomic information, but rather on the combination of the genomic, medical, and demographic information of the patient. While previous studies have used a limited number of key features as a baseline for imputation of a phenotype (e.g., age, sex, and major comorbidities)^49,65–67^, to the best of our knowledge, these studies did not take into account the entire familial-genetic or environmental history of the patients. Thus, the question of whether genomic data (methylation or genetics) can be used to improve imputation over the EHR data is critical in order to claim clinical relevance. Our results demonstrate that adding MRS to existing EHR-based imputation frameworks improve imputation accuracy by over 47% in a clinical context.

It is well appreciated that PRS are sensitive to the studied population, and it is often the case that a PRS developed for one ethnic group performs poorly on others^55,57^. It is therefore important to evaluate the population effect on MRS performance. For this reason, we measured the transferability of our results across different populations, and we observe that the accuracy of the MRS was robust to population structure. This is likely driven by the diversity of the training cohort used, but also due to the fact that we are under-powered to discover subtle differences in imputation accuracy due to our sample sizes. Nonetheless, since we observed very few large differences in accuracy across populations, we are hopeful that our results will inspire future investigations to continue to recruit diverse cohorts and to examine these differences at length with greater sample sizes.

While our study was focused on methylation, there are many other possibilities for the introduction of genomic data in clinical settings. First and foremost, genetic data has been heavily studied by others and large biobanks including genetic data of patients already exist. However, other measurements such as RNA, microbiome, metabolomics, or proteomics may also be relevant. Some of these have logistic and cost considerations at scale. One of the advantages of methylation is that DNA biobanks already exist in large numbers, and the cost of measuring methylation is close to that of measuring genetic data. Moreover, different genomic measurements may provide different snapshots of the patient’s data, risk, or health status. Methylation, for example, is known to capture one’s smoking status^26^, and may therefore be particularly useful for cases in which researchers intend to use self-reported features that may suffer from patient recall bias or honesty. Tangentially, while polygenic risk scores provide a lifetime risk for a patient, methylation risk scores may provide the current risk of the patient over the last few months^68–70^, and other genomic information may provide risk with the resolution of days or hours (e.g., RNA or certain metabolomics^71–74^). Nonetheless, owing to the dynamic nature of methylation, it is currently unclear what the range or duration of the methylation risk scores are. Furthermore, while methylation patterns are associated with outcomes, it is generally unknown if they cause a disease or are a response to a disease^75^.

To assist the research community in investigating methylation in the context of disease, we provide the MRS predictors for all significantly predicted outcomes at https://github.com/cozygene/EHR_MRS_UCLA. While our samples were ascertained on kidney and heart disease, we show that our weights successfully replicated across three external datasets, including studies of Rheumatoid Arthritis and Schizophrenia. Consequently, our weights may be used by researchers and clinicians in different ways. For example, in many epigenome-wide association studies (EWAS), in which associations between specific methylation CpG sites and a phenotype are studied, one may wish to account for patients’ comorbidities and medications, which are often not available to the study. Using the MRS database, the researchers leveraging EWAS will be able to incorporate such covariates into their model.

There are multiple potential next steps for the examination of methylation in clinical contexts. First, in this work, we focused our attention on the imputation of the phenotypes, or in other words, the inference as to whether the patient is currently diagnosed with a disease. We hope that our findings will be able to be translated to the inference of future clinical events, i.e., prediction of future deterioration or disease occurrence. Second, our analyses did not focus on generating models for a specific patient demographic (e.g., only senior patients) and we were limited to methylation collected from blood samples. As methylation is known to vary across age and tissue type, models may be improved by focusing on individuals of a specific demographic, or by assaying a tissue relevant for a given phenotype (e.g., liver tissue for metabolic disorders). Third, although our evaluation is across the largest dataset which includes EHR, methylation, and genotype data, the sample size of our study is still moderate compared to genetic studies that are performed on biobanks. Indeed, we demonstrate that for some of the phenotypes, an increase in sample size will likely lead to a substantially improved imputation accuracy (Fig. 3; Supplementary Figs. 6–8). Moreover, larger sample size data may be able to reveal the quantity or contribution of genetics verses methylation to the MRS imputation accuracy^48^. In light of our results, as well as the fact that many biobanks have already obtained blood or DNA samples, we recommend that future biobanks consider measuring methylation in addition to the genotypes across a large number of patients.

## Methods

### Electronic health record data

De-identified electronic health record data for this study was extracted from the perioperative data warehouse (PDW), a custom-built, robust data warehouse containing all patients who have undergone surgery at UCLA Health since the implementation of UCLA’s EMR (EPIC Systems, Madison, WI, USA) in March 2013. The PDW, which has been described previously^76^, has a two-stage design. First, data are extracted from EPIC’s Clarity database into 29 tables organized around three distinct concepts: patients, surgical procedures, and health system encounters. Then, these data are used to populate a series of 4000 distinct measures and metrics such as procedure duration, admission ICD codes, lab results, and medication orders.

### Patient ascertainment

Methylation and genotype samples were collected using blood from 831 patients as part of the UCLA ATLAS precision health initiative between October 26, 2016 and December 10, 2018^77^. We include the following statements from ref. ^77^ detailing IRB approval. Retrospective data collection and analysis was approved by the UCLA IRB. Patient Recruitment and Sample Collection for Precision Health Activities at UCLA is an approved study by the UCLA Institutional Review Board (UCLA IRB). IRB17-001013. All necessary patient/participant consent has been obtained and the appropriate institutional forms have been archived.

The samples were collected from patients before undergoing surgery with general anesthesia at UCLA Health, and the patients had not undergone surgery in the 30 days prior to blood sample collection. Of these patients, 302 were selected for inclusion based on the presence of acute kidney injury (AKI), defined as an Acute Kidney Injury Network (AKIN) classification of one or greater, after undergoing surgery. An additional 348 patients were risk-matched controls, with either glomerular filtration rate (GFR) less than or equal to 38 (210 patients), or GFR >38 and a propensity risk score that matched case patients (348 patients). The propensity score was created using available EHR features such as age, weight, BMI, and other preoperative features that were measured in the hospital. Within the control group, we also performed a similar procedure ascertained on whether individuals were a heart attack case. Controls for heart attack patients were also selected using propensity scoring. Demographics of the patient population are further described in Supplementary Table 1.

### Medication usage

For each medication, a patient was labeled as using a medication if the electronic health record contained a medication order that occurred before the methylation sample collection date. Medications were grouped by pharmaceutical subclass using the Generic Product Identifier (GPI) hierarchical classification system codes. Any medications that were ordered in fewer than 5% of the patients were excluded from the analysis. In total, 168 pharmaceutical subclasses were considered in our analysis. The number of patients using medications from each subclass is shown in Supplementary Table 8. In Supplementary Table 9, we show for each pharmaceutical subclass the specific medication that patients in our cohort received.

### Lab results

The most recent lab result prior to the methylation sample collection was extracted from the PDW for each patient. Any labs with a result date that occurred more than 365 days before the methylation sample collection date were excluded from the analyses. In addition, labs for which there were <50 patients with valid results were excluded. We were left with a total of 69 lab values on which to run our models.

### Diagnosis codes

International Classification of Diseases, Ninth Revision (ICD-9) and International Classification of Diseases, Tenth Revision (ICD-10) codes are a standard set of diagnosis codes, primarily used for billing purposes. While these codes provide a standardized methodology for describing a diagnosis, they are very specific. To map these specific diagnosis codes into meaningful, distinct diseases/traits, Denny et al. aggregated the ICD codes into phenotype codes (Phecodes)^42,43^. Specifically, for each patient, we queried all diagnoses prior to the methylation sample collection date, and used the Phecode (version 1.2) mapping to aggregate ICD-9 and ICD-10 codes to unique, meaningful phenotypes. If a patient’s diagnosis record had both ICD-9 and ICD-10 labels, the ICD-10 to Phecode mapping was used instead of the ICD-9 to Phecode mapping. Each Phecode was treated as a binary variable, indicating the presence or absence of a relevant diagnosis code at any point in time before sample collection. We excluded rare Phecodes (occurrence in <5% of the patients) and, in total, our cohort contained 370 unique Phecode phenotypes.

### Preprocessing of genotype data for cross-validation

We measured the genotypes for 831 individuals based on their DNA sampled from whole blood using the ATLAS genotype array. We preprocessed the genotype data using Beagle (d20)^78^, PLINK (1.07)^54^, and GCTA (1.93.2)^79^. We restricted the genotypes to autosomal variants, removed rare variants (MAF <0.05), and filtered for variants that met Hardy-Weinberg equilibrium with *p*-value threshold 10^−6^. We also removed individuals and variants with more than 1% missing values. For the purpose of running cross-validation, we used Beagle to impute only any remaining missing values, but did not impute to an external dataset. We show that with our sample size and phenotypes evaluated, using genotypes imputed to an external reference does not significantly improve our results (Supplementary Fig. 14). In total we were left with 292,808 SNPs. To obtain principal components, we ran PCA using plink on the chipped genotypes.

### Preprocessing and imputation of genotype data for comparison to external models

We used a version of the ATLAS genotype data that was imputed to an external dataset, as detailed in ref. ^77^. Briefly, after performing quality control, genotypes were uploaded to the Michigan Imputation Server^80^. The server phases the genotype data using Eagle v2.4^81^ and performs imputation using the TOPMed Freeze5 imputation panel^82^ using minimac4^83^. We applied the same quality control and filters to the imputed genotypes as we did the chipped genotypes, and we were left with a total of 5,574,956 SNPs.

### Preprocessing of methylation array data

We measured methylation data for 831 individuals based on their DNA sampled from whole blood using the EPIC Illumina array. To generate beta-normalized methylation levels at each CpG, we ran the default pipeline of ENmix (1.22.0)^84^ on the raw probe data (IDAT files), which performs background correction, RELIC dye bias correction, and RCP probe-type bias adjustment. We removed from our analysis CpGs that coincided with SNP loci as well as CpGs on the sex chromosomes. We also filtered out outlier samples, defined as having a PC score more than 4 standard deviations away from the average PC score in the first two principal components. In the imputation tasks, we removed sites with low variability (standard deviation < 0.02) leading to a total of 269,471 sites.

### Imputation using baseline medical features

To establish a baseline level of imputation performance, we constructed a set of features derived from basic patient information. We trained a simple linear (or logistic) model with 10-fold cross validation using an intercept and patients’ age, sex, BMI, methylation-based cell-type proportions (from the reference-based method of Houseman et al.^41^), self-reported ancestry, first ten genetic principal components, and smoking status (never, former or current). Importantly, we wished to establish how well an outcome (medication, Phecode, or lab value) could be imputed by using covariates (e.g., ancestry, age, smoking status) that are known to be captured by genomics.

### Imputation using a single penalized linear model

After establishing a baseline level of imputation performance, we performed penalized logistic and linear regression using either individuals’ methylation or genotypes. More concretely, we fit 10-fold cross-validation using LASSO, elastic net and ridge regularization under the following two models:

2 $$y={\alpha }_{G}+G{\beta }_{G}+C{\beta }_{C}+{\varepsilon }_{G}$$

3 $$y={\alpha }_{M}+M{\beta }_{M}+C{\beta }_{C}+{\varepsilon }_{M}$$

where *y* corresponds to the outcome, *α* the model-specific intercept, *G* the *n* × *s* genotypes, *M* the *n* × *c* methylation data, *β* the vector of length-*s* or -*c* effect sizes for the given explanatory variable, *C* and *β*_*C*_ the covariates from the baseline model and their corresponding effect sizes, and *ε* the length *n* noise vector. We refer to models (2) and (3) as the PRS and MRS, respectively, and note that they also include the baseline features. After fitting all three penalized linear models (LASSO, elastic net, and ridge) for a given datatype and outcome, we selected a final model as determined by the model with the highest cross-validated metric (AUC or *R*^2^ if the outcome was binary or continuous, respectively). We fit all penalized models using package *bigstatsr*^40^. We share MRS weights for outcomes that were significantly imputed at https://github.com/cozygene/EHR_MRS_UCLA. We also include details on the number of CpGs selected for each MRS in these analyses in Supplementary Tables 5, 6, and 7.

### Imputing lab results using EHR data and MRS values with softImpute

Imputing a partially-observed matrix of values is often formulated as a matrix-completion problem. In a matrix completion problem, the observed values of the matrix are used to estimate the values of the unobserved values by assuming that there is some underlying structure that is responsible for generating the data. For example, in the popular SoftImpute method^38^, the data is assumed to be well-approximated by a low-rank representation, and the error between the observed values and the reconstructed values is minimized through a convex optimization procedure. However, since the unobserved values are, by definition, not observed, and therefore cannot be used to assess the imputation performance, the primary method for measuring the performance involves masking (removing) observed values and comparing the imputed values to these held-out, true values.

The EHR data used in the imputation procedure included demographic information, diagnosis codes, medication usage, and lab results, which were extracted from the EHR database using the previously described criteria. In addition to the EHR data, we also ran the imputation procedure while including relevant MRS values. Specifically, we included the MRS values for demographics, diagnosis codes, medication usage, and lab results that were imputed at a statistically significant level. These MRS values were added as additional observed features to the EHR matrix.

To estimate the imputation performance, we randomly masked 10% of the observed lab result values, and performed the imputation procedure (SoftImpute matrix completion) to generate estimates of the missing values. However, since labs are most often ordered in panels, for example a metabolic panel, if a lab is missing then typically other labs that are part of the same panel are also missing. We simulated a more realistic missingness scenario by, instead of masking out values only from a specific lab *l*, masking out all labs that are ordered as a panel that include lab *l*. This masking procedure was done per lab, using 10-fold cross-validation, such that 10% of the non-missing values of a particular lab result (and its associated lab panels) were masked (removed), and the remaining 90% of the observed values were used to complete the matrix. Matrix completion was performed using the SoftImpute algorithm, as implemented in the *fancyimpute*^85^ python package (version 0.5.5). The proportion of variance explained (*R*^2^) of the true lab values by the imputed lab values was used to measure the imputation performance. Confidence intervals were derived using bootstrapping.

### Hypothesis testing

To determine whether an imputation was significant or whether one predictor offered significant additional explanatory signal, we conducted our hypothesis tests using a linear (logistic) regression framework. Primarily, after running cross-validation or generating a single predictor $\hat{y}$ for an outcome *y*, we would test whether the imputation was significant by comparing it to *y*:

4 $$y=\alpha +\hat{y}\beta +\varepsilon$$

Where Eq. (4) corresponded to linear regression when the outcome was continuous, and logistic regression when the outcome was binary, *α* was the intercept, and *β* was an effect size indicating association of the predictor with the outcome. Notably, by building our testing framework as a linear model, we can easily extend it to include additional predictors in order to test whether the additional predictors significantly improve the fit of the regression—or more simply, whether predictor $\hat{{y}_{j}}$ offers additional predictive power over $\hat{{y}_{i}}$ by conducting a likelihood ratio test of the following nested models:

5 $$y={\alpha }_{i}+\hat{{y}_{i}}{\beta }_{i}+{\varepsilon }_{i}$$

6 $$y={\alpha }_{ij}+\hat{{y}_{i}}{\beta }_{i}+\hat{{y}_{j}}{\beta }_{j}+{\varepsilon }_{ij}$$

Where *i* and *j* index either the baseline, MRS, or PRS models. We corrected for multiple hypothesis tests within each outcome and genomic risk score by using a Bonferroni adjustment at *α* level 0.05.

### Imputing external polygenic risk scores into the ATLAS cohort

We compared our in-house built risk scores to risk scores learned in the UKBiobank dataset^50,52^. In both^50,52^, the authors construct their PRS using penalized regression akin to as we have done in our analyses. Notably, using penalized regression on individual-level genotypes allows one to automatically, optimally control for shrinkage and variable selection at the step of model generation^40,86^. This is in contrast with many commonly used polygenic risk score tools such as LDPred^87^ or PRSCS^88^, that attempt to perform shrinkage or variable selection post hoc on the level of summary statistics. After downloading the PRS from the PGS catalog^53^ listed in Supplementary Table 10, we imputed PRS into our cohort using our imputed genotypes using the score function of plink. To account for population structure, we limited our analysis to individuals who self-identified as white, and passed filtering using manual inspection of principal components (Supplementary Fig. 9).

### Reporting summary

Further information on research design is available in the Nature Research Reporting Summary linked to this article.

## Supplementary information

Supplement

Reporting Summary

## Data Availability

The data behind this study are part of the UCLA ATLAS project. The individual-level data are available behind the UCLA Mednet firewall and portal, but are prohibited by the IRB for external sharing at the individual level. Non-UCLA-affiliated researchers may access the data through collaborative studies or by becoming a UCLA Mednet contractor or employee. The data used for the replication analysis are publicly available via the Gene Expression Omnibus (T1D - GSE20067; RA - GSE42861; SZ - GSE80417, GSE84727).

## Code availability

The MRS weights for outcomes that were significantly imputed are located at: https://github.com/cozygene/EHR_MRS_UCLA. Codes are available from the corresponding author upon reasonable request.

## References

[1] Sudlow C (2015). UK Biobank: an open access resource for identifying the causes of a wide range of complex diseases of middle and old age. PLOS Med..

[2] McCarty CA, Wilke RA, Giampietro PF, Wesbrook SD, Caldwell MD (2005). Marshfield Clinic Personalized Medicine Research Project (PMRP): design, methods and recruitment for a large population-based biobank. Per. Med..

[3] Roden DM (2008). Development of a large-scale de-identified DNA biobank to enable personalized medicine. Clin. Pharmacol. Ther..

[4] Bastarache L (2018). Phenotype risk scores identify patients with unrecognized Mendelian disease patterns. Science.

[5] Hulsen T (2019). From big data to precision medicine. Front. Med..

[6] Liang H (2019). Evaluation and accurate diagnoses of pediatric diseases using artificial intelligence. Nat. Med..

[7] Clark MM (2019). Diagnosis of genetic diseases in seriously ill children by rapid whole-genome sequencing and automated phenotyping and interpretation. Sci. Transl. Med..

[8] Corey KM (2018). Development and validation of machine learning models to identify high-risk surgical patients using automatically curated electronic health record data (Pythia): a retrospective, single-site study. PLOS Med..

[9] Hill BL (2019). An automated machine learning-based model predicts postoperative mortality using readily-extractable preoperative electronic health record data. Br. J. Anaesth..

[10] Khera AV (2018). Genome-wide polygenic scores for common diseases identify individuals with risk equivalent to monogenic mutations. Nat. Genet..

[11] Mavaddat N (2019). Polygenic risk scores for prediction of breast cancer and breast cancer subtypes. Am. J. Hum. Genet..

[12] Lewis CM, Hagenaars SP (2019). Progressing polygenic medicine in psychiatry through electronic health records. JAMA Psychiatry.

[13] Lewis CM, Vassos E (2020). Polygenic risk scores: from research tools to clinical instruments. Genome Med..

[14] Kertai MD (2021). Predictive accuracy of a polygenic risk score for postoperative atrial fibrillation after cardiac surgery. Circ. Genom. Precis. Med..

[15] Hatib F (2018). Machine-learning algorithm to predict hypotension based on high-fidelity arterial pressure waveform analysis. Anesthesiology.

[16] Wijnberge M (2020). Effect of a machine learning-derived early warning system for intraoperative hypotension vs standard care on depth and duration of intraoperative hypotension during elective noncardiac surgery: the hype randomized clinical trial. JAMA.

[17] Gulshan V (2016). Development and validation of a deep learning algorithm for detection of diabetic retinopathy in retinal fundus photographs. JAMA.

[18] Beede, E. et al. A human-centered evaluation of a deep learning system deployed in clinics for the detection of diabetic retinopathy. in *Proceedings of the 2020 CHI Conference on Human Factors in Computing Systems* 1–12 (Association for Computing Machinery, 2020).

[19] Ghorbani A (2020). Deep learning interpretation of echocardiograms. npj Digit. Med..

[20] Ribeiro AH (2020). Automatic diagnosis of the 12-lead ECG using a deep neural network. Nat. Commun..

[21] Hannun AY (2019). Cardiologist-level arrhythmia detection and classification in ambulatory electrocardiograms using a deep neural network. Nat. Med..

[22] Komorowski, M. et al. The Artificial Intelligence Clinician learns optimal treatment strategies for sepsis in intensive care. *Nat. Med.*10.1038/s41591-018-0213-5 (2018). 10.1038/s41591-018-0213-5 30349085

[23] Maas P (2016). Breast cancer risk from modifiable and nonmodifiable risk factors among white women in the United States. JAMA Oncol..

[24] Schumacher FR (2018). Association analyses of more than 140,000 men identify 63 new prostate cancer susceptibility loci. Nat. Genet..

[25] Hüls A, Czamara D (2020). Methodological challenges in constructing DNA methylation risk scores. Epigenetics.

[26] Lee K, Pausova Z (2013). Cigarette smoking and DNA methylation. Front. Genet..

[27] Galanter JM (2017). Differential methylation between ethnic sub-groups reflects the effect of genetic ancestry and environmental exposures. eLife.

[28] Hibler E (2019). Impact of a diet and activity health promotion intervention on regional patterns of DNA methylation. Clin. Epigenet..

[29] White AJ (2013). Recreational and household physical activity at different time points and DNA global methylation. Eur. J. Cancer.

[30] Zhang FF (2011). Dietary patterns are associated with levels of global genomic DNA methylation in a cancer-free population. J. Nutr..

[31] Dick KJ (2014). DNA methylation and body-mass index: a genomewide analysis. Lancet.

[32] Levenson VV (2010). DNA methylation as a universal biomarker. Expert Rev. Mol. Diagn..

[33] Kamińska K (2019). Prognostic and predictive epigenetic biomarkers in oncology. Mol. Diagn. Ther..

[34] Chu AY (2017). Epigenome-wide association studies identify DNA methylation associated with kidney function. Nat. Commun..

[35] Liu Y (2013). Epigenome-wide association data implicate DNA methylation as an intermediary of genetic risk in rheumatoid arthritis. Nat. Biotechnol..

[36] Rakyan VK (2011). Identification of type 1 diabetes-associated DNA methylation variable positions that precede disease diagnosis. PLOS Genet..

[37] Huynh JL (2014). Epigenome-wide differences in pathology-free regions of multiple sclerosis-affected brains. Nat. Neurosci..

[38] Mazumder R, Hastie T, Tibshirani R (2010). Spectral regularization algorithms for learning large incomplete matrices. J. Mach. Learn. Res..

[39] Dudbridge F (2013). Power and predictive accuracy of polygenic risk scores. PLOS Genet..

[40] Privé F, Aschard H, Ziyatdinov A, Blum MGB (2018). Efficient analysis of large-scale genome-wide data with two R packages: bigstatsr and bigsnpr. Bioinformatics.

[41] Houseman EA (2012). DNA methylation arrays as surrogate measures of cell mixture distribution. BMC Bioinform..

[42] Denny JC (2010). PheWAS: demonstrating the feasibility of a phenome-wide scan to discover gene-disease associations. Bioinformatics.

[43] Denny JC (2013). Systematic comparison of phenome-wide association study of electronic medical record data and genome-wide association study data. Nat. Biotechnol..

[44] Beutler E, West C (2005). Hematologic differences between African-Americans and whites: the roles of iron deficiency and alpha-thalassemia on hemoglobin levels and mean corpuscular volume. Blood.

[45] Lim E, Miyamura J, Chen JJ (2015). Racial/ethnic-specific reference intervals for common laboratory tests: a comparison among Asians, blacks, Hispanics, and White. Hawaii J. Med. Public Health.

[46] Horvath S (2013). DNA methylation age of human tissues and cell types. Genom. Biol..

[47] Singmann P (2015). Characterization of whole-genome autosomal differences of DNA methylation between men and women. Epigenetics Chromatin.

[48] Trejo Banos D (2020). Bayesian reassessment of the epigenetic architecture of complex traits. Nat. Commun..

[49] Beaulieu-Jones BK (2018). Characterizing and managing missing structured data in electronic health records: data analysis. JMIR Med. Inform..

[50] Tanigawa Y (2022). Significant sparse polygenic risk scores across 813 traits in UK biobank. PLOS Genet..

[51] Vuckovic D (2020). The polygenic and monogenic basis of blood traits and diseases. Cell.

[52] Sinnott-Armstrong N (2021). Genetics of 35 blood and urine biomarkers in the UK biobank. Nat. Genet..

[53] Lambert SA (2021). The polygenic score catalog as an open database for reproducibility and systematic evaluation. Nat. Genet..

[54] Purcell S (2007). PLINK: a tool set for whole-genome association and population-based linkage analyses. Am. J. Hum. Genet..

[55] Duncan L (2019). Analysis of polygenic risk score usage and performance in diverse human populations. Nat. Commun..

[56] Kerminen S (2019). Geographic variation and bias in the polygenic scores of complex diseases and traits in Finland. Am. J. Hum. Genet..

[57] Martin AR (2019). Clinical use of current polygenic risk scores may exacerbate health disparities. Nat. Genet..

[58] Rahmani E (2017). Genome-wide methylation data mirror ancestry information. Epigenetics Chromatin.

[59] Barfield RT (2014). Accounting for population stratification in DNA methylation studies. Genetic Epidemiol..

[60] Moen EL (2013). Genome-wide variation of cytosine modifications between European and African populations and the implications for complex traits. Genetics.

[61] Bell CG (2010). Genome-wide DNA methylation analysis for diabetic nephropathy in type 1 diabetes mellitus. BMC Med. Genom..

[62] Hannon E (2016). An integrated genetic-epigenetic analysis of schizophrenia: evidence for co-localization of genetic associations and differential DNA methylation. Genome Biol..

[63] Liu Y (2013). Epigenome-wide association data implicate DNA methylation as an intermediary of genetic risk in rheumatoid arthritis. Nat. Biotechnol..

[64] Belsky DW (2017). Translating polygenic analysis for prevention: from who to how. Circ. Cardiovasc. Genet..

[65] Jazayeri A, Liang OS, Yang CC (2020). Imputation of missing data in electronic health records based on patients’ similarities. J. Health. Inform. Res..

[66] Miotto R, Li L, Kidd BA, Dudley JT (2016). Deep patient: an unsupervised representation to predict the future of patients from the electronic health records. Sci. Rep..

[67] Zheng T (2017). A machine learning-based framework to identify type 2 diabetes through electronic health records. Int. J. Med. Inf..

[68] Rahmani E (2016). Sparse PCA corrects for cell type heterogeneity in epigenome-wide association studies. Nat. Methods.

[69] Horvath S (2013). DNA methylation age of human tissues and cell types. Genome Biol..

[70] Fitzgerald KN (2021). Potential reversal of epigenetic age using a diet and lifestyle intervention: a pilot randomized clinical trial. Aging.

[71] Li, J., Grant, G. R., Hogenesch, J. B. & Hughes, M. E. in *Methods in Enzymology* (ed. Sehgal, A.) Vol. 551, 349–367 (Academic Press, 2015). 10.1016/bs.mie.2014.10.020 25662464

[72] Couto Alves A, Glastonbury CA, El-Sayed Moustafa JS, Small KS (2018). Fasting and time of day independently modulate circadian rhythm relevant gene expression in adipose and skin tissue. BMC Genom..

[73] Chaleckis R, Murakami I, Takada J, Kondoh H, Yanagida M (2016). Individual variability in human blood metabolites identifies age-related differences. Proc. Natl Acad. Sci. USA.

[74] Asher G, Sassone-Corsi P (2015). Time for food: the intimate interplay between nutrition, metabolism, and the circadian clock. Cell.

[75] Relton CL, Davey Smith G (2012). Two-step epigenetic Mendelian randomization: a strategy for establishing the causal role of epigenetic processes in pathways to disease. Int. J. Epidemiol.

[76] Hofer, I. S., Gabel, E., Pfeffer, M., Mahbouba, M. & Mahajan, A. A systematic approach to creation of a perioperative data warehouse. *Anesth. Analg.* **122**, https://journals.lww.com/anesthesia-analgesia/Fulltext/2016/06000/A_Systematic_Approach_to_Creation_of_a.25.aspx (2016). 10.1213/ANE.0000000000001201 27195633

[77] Johnson, R. et al. Leveraging genomic diversity for discovery in an EHR-linked biobank: the UCLA ATLAS community health initiative. Preprint at *medRxiv*10.1101/2022.02.12.22270895 (2021).

[78] Browning SR, Browning BL (2007). Rapid and accurate haplotype phasing and missing-data inference for whole-genome association studies by use of localized haplotype clustering. Am. J. Hum. Genet..

[79] Yang J, Lee SH, Goddard ME, Visscher PM (2011). GCTA: a tool for genome-wide complex trait analysis. Am. J. Hum. Genet..

[80] Das S (2016). Next-generation genotype imputation service and methods. Nat. Genet..

[81] Loh P-R (2016). Reference-based phasing using the haplotype reference consortium panel. Nat. Genet..

[82] Taliun D (2021). Sequencing of 53,831 diverse genomes from the NHLBI TOPMed program. Nature.

[83] Fuchsberger C, Abecasis GR, Hinds DA (2015). minimac2: faster genotype imputation. Bioinformatics.

[84] Xu Z, Niu L, Li L, Taylor JA (2016). ENmix: a novel background correction method for Illumina HumanMethylation450 BeadChip. Nucleic Acids Res..

[85] Rubinsteyn, A., Feldman, S., O’Donnell, T. & Beaulieu-Jones, B. hammerlab/fancyimpute: Version 0.2.0. https://zenodo.org/record/886614#.WtfmOC-ZNTY (2017).

[86] Abraham G, Kowalczyk A, Zobel J, Inouye M (2012). SparSNP: fast and memory-efficient analysis of all SNPs for phenotype prediction. BMC Bioinform..

[87] Vilhjálmsson BJ (2015). Modeling linkage disequilibrium increases accuracy of polygenic risk scores. Am. J. Hum. Genet..

[88] Ge T, Chen C-Y, Ni Y, Feng Y-CA, Smoller JW (2019). Polygenic prediction via bayesian regression and continuous shrinkage priors. Nat. Commun..

