# Supplementary material for: Methylation risk scores are associated with a collection of phenotypes within electronic health record systems
Source: NPJ Genom Med. 2022 Aug 25;7:50. doi: 10.1038/s41525-022-00320-1 (PMC9411568; doi:10.1038/s41525-022-00320-1)
Supplement: Supplementary file 1 — Supplement [file 41525_2022_320_MOESM1_ESM.pdf]

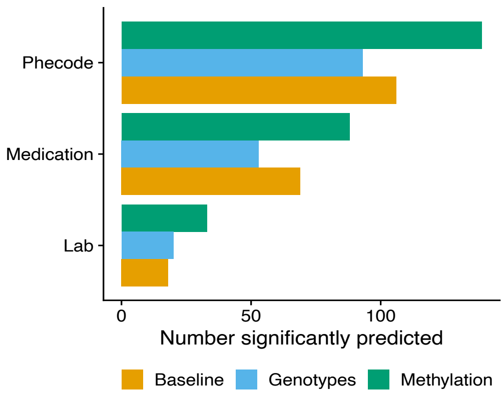

Supplementary Figure 1. Significantly predicted outcomes per data type Total number of significantly predicted outcomes when using the baseline alone, as well as including either set of genomic features in addition to the baseline. We used an association test of the cross-validated predictors and the true outcome and adjusted for multiple testing using Bonferroni correction at a nominal threshold of 0.05.

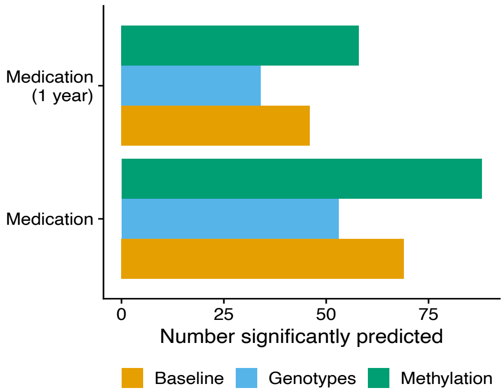

Supplementary Figure 2. Significantly predicted medications prescribed within one year of collection date or EHR history Total number of significantly predicted medications when using the baseline alone, as well as including either set of genomic features in addition to the baseline. We used an association test of the cross-validated predictors and the true outcome and adjusted for multiple testing using Bonferroni correction at a nominal threshold of 0.05. Using a patient's entire EHR history to generate their list of medications resulted in more significant associations than using just the year prior to sample collection date.

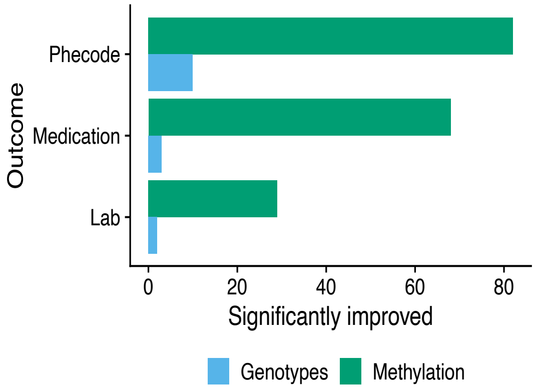

Supplementary Figure 3. Number of outcomes significantly improved when adding the corresponding predictor Similarly to the tests in which we surveyed whether the MRS or PRS predictors added predictive power over the baseline predictor, we conducted an analysis in which we examined whether the MRS predictor adds predictive power over the PRS predictor (Green "Methylation"), and whether the PRS predictor adds predictive power over the MRS predictor (Blue "Genotypes"). The MRS predictor improved the performance of the PRS predictor for a substantial number of outcomes

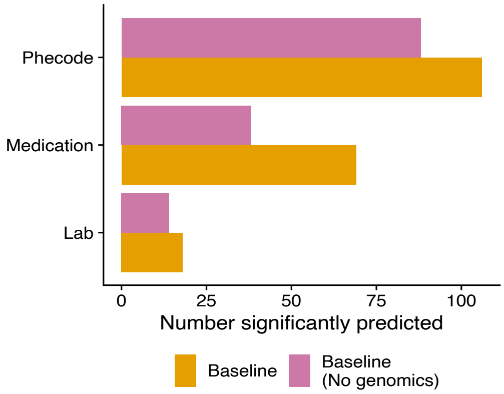

Supplementary Figure 4. Significantly predicted outcomes across baseline models Total number of significantly predicted outcomes when using the baseline model with and without variables derived from genomics (ancestry principal components, methylation-estimated cell-type composition estimates). We used an association test of the cross-validated predictors and the true outcome and adjusted for multiple testing using Bonferroni correction at a nominal threshold of 0.05.

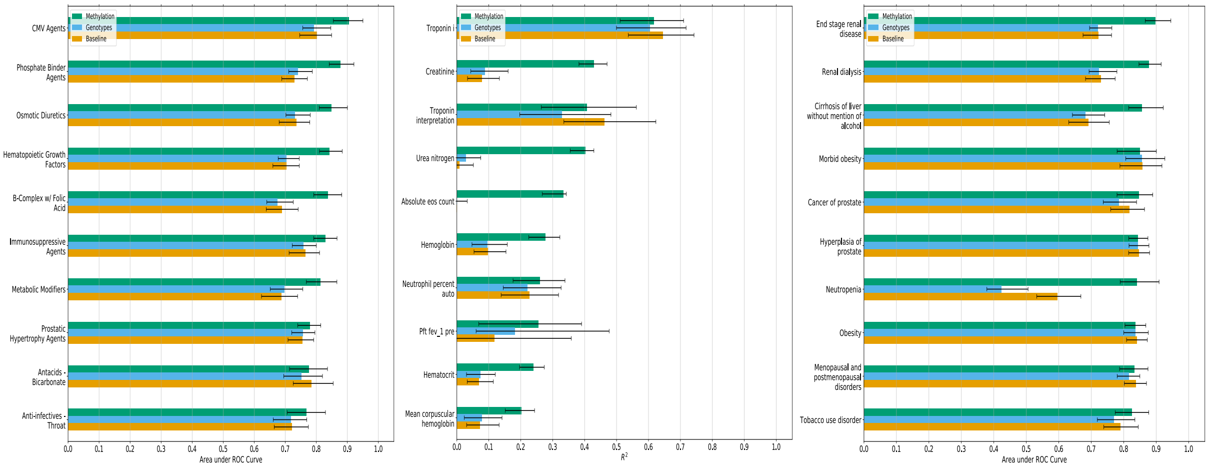

Supplementary Figure 5. Significantly-predicted outcomes per data type The top 10 methylation-predicted (Left) medications, (Middle) labs, and (Right) Phecodes, with Baseline and Genotype prediction performance results for comparison. Error bars indicate 95% confidence intervals.

Feature	Value	Missing	Overall
n			831
Age, mean (SD)		0	61.0 (15.8)
Sex, n (%)	F	0	352 (42.4)
	M		479 (57.6)
BMI, mean (SD)		1	27.2 (6.6)
AKIN Classification, n (%)	0.0	0	537 (64.6)
	1.0		189 (22.7)
	2.0		27 (3.2)
	3.0		78 (9.4)
GFR > 38, n (%)	False	0	375 (45.1)
	True		456 (54.9)
Heart Attack, n (%)	False	601	146 (63.5)
	True		84 (36.5)
Self-Reported Ethnicity, n (%)	Cuban	0	2 (0.2)
	Hispanic or Latino		116 (14.0)
	Hispanic/Spanish origin Other		14 (1.7)
	Mexican, Mexican American, Chicano/a		37 (4.5)
	Not Hispanic or Latino		655 (78.8)
	Patient Refused		5 (0.6)
	Puerto Rican		2 (0.2)
Self-Reported Race, n (%)	American Indian	0	2 (0.2)
	Asian		73 (8.8)
	Black		72 (8.7)
	Declined to Specify		6 (0.7)
	Other Race		132 (15.9)
	Pacific Islander		3 (0.4)
	Unknown		1 (0.1)
	White or Caucasian		542 (65.2)

Supplementary Table 1. Cohort patient demographics AKIN is the Acute Kidney Injury Network Classification, BMI is Body Mass Index, GFR is glomerular filtration rate.

Supplementary Table 2. Mean (95% confidence interval) area under the ROC curve for predicting medication usage, grouped by pharmaceutical subclass, using the baseline, methylation data, and genotype data. Confidence intervals determined using bootstrapping.

Pharmaceutical Subclass	Baseline	Methylation	Genotypes
CMV Agents	0.80 (0.75-0.85)	0.90 (0.86-0.94)	0.79 (0.74-0.84)
Phosphate Binder Agents	0.73 (0.68-0.77)	0.88 (0.84-0.91)	0.74 (0.70-0.78)
Osmotic Diuretics	0.74 (0.69-0.78)	0.85 (0.81-0.88)	0.73 (0.68-0.78)
Hematopoietic Growth Factors	0.70 (0.66-0.75)	0.84 (0.81-0.87)	0.70 (0.66-0.75)
B-Complex w/ Folic Acid	0.69 (0.64-0.74)	0.84 (0.79-0.87)	0.67 (0.62-0.72)
Immunosuppressive Agents	0.77 (0.72-0.81)	0.83 (0.79-0.86)	0.76 (0.70-0.80)
Metabolic Modifiers	0.69 (0.63-0.74)	0.81 (0.77-0.86)	0.70 (0.63-0.75)
Prostatic Hypertrophy Agents	0.76 (0.72-0.79)	0.78 (0.74-0.81)	0.76 (0.71-0.79)
Antacids - Bicarbonate	0.78 (0.71-0.85)	0.78 (0.71-0.83)	0.75 (0.69-0.81)
Anti-infectives - Throat	0.72 (0.67-0.77)	0.77 (0.71-0.82)	0.72 (0.66-0.78)
Cycloplegic Mydriatics	0.76 (0.71-0.82)	0.75 (0.68-0.81)	0.76 (0.69-0.82)
Thrombolytic Enzymes	0.67 (0.60-0.74)	0.75 (0.68-0.81)	0.63 (0.56-0.70)
Plasma Proteins	0.67 (0.62-0.72)	0.74 (0.69-0.78)	0.66 (0.61-0.71)
Potassium Removing Agents	0.64 (0.59-0.70)	0.74 (0.69-0.79)	0.62 (0.56-0.67)
Cephalosporins - 4th Generation	0.67 (0.59-0.74)	0.73 (0.64-0.80)	0.65 (0.57-0.74)
Gallstone Solubilizing Agents	0.59 (0.50-0.69)	0.72 (0.64-0.79)	0.50 (0.41-0.59)
Imidazole-Related Antifungals	0.69 (0.64-0.73)	0.72 (0.67-0.77)	0.68 (0.63-0.73)
HMG CoA Reductase Inhibitors	0.72 (0.67-0.75)	0.72 (0.68-0.75)	0.71 (0.68-0.74)
Alkalinizers	0.70 (0.62-0.76)	0.71 (0.63-0.78)	0.68 (0.59-0.75)
Bone Density Regulators	0.70 (0.63-0.75)	0.71 (0.65-0.75)	0.70 (0.64-0.76)
Parenteral Therapy Supplies	0.52 (0.45-0.59)	0.71 (0.65-0.76)	0.60 (0.54-0.66)
Vasodilators	0.54 (0.49-0.59)	0.70 (0.66-0.75)	0.48 (0.43-0.52)
Salicylates	0.69 (0.65-0.72)	0.70 (0.66-0.73)	0.68 (0.64-0.72)
Ophthalmic Local Anesthetics	0.69 (0.63-0.76)	0.69 (0.63-0.76)	0.68 (0.61-0.76)
Proton Pump Inhibitors	0.62 (0.58-0.66)	0.69 (0.66-0.73)	0.61 (0.57-0.66)

Continued on next page

Supplementary Table 2. Mean (95% confidence interval) area under the ROC curve for predicting medication usage, grouped by pharmaceutical subclass, using the baseline, methylation data, and genotype data. Confidence intervals determined using bootstrapping.

Pharmaceutical Subclass	Baseline	Methylation	Genotypes
Impotence Agents	0.70 (0.64-0.75)	0.68 (0.62-0.74)	0.67 (0.60-0.73)
Ophthalmic Steroids	0.70 (0.64-0.75)	0.68 (0.62-0.73)	0.68 (0.62-0.74)
Diabetic Supplies	0.64 (0.58-0.69)	0.67 (0.62-0.72)	0.62 (0.56-0.67)
Phosphate	0.65 (0.57-0.72)	0.67 (0.60-0.74)	0.60 (0.52-0.66)
Loop Diuretics	0.63 (0.60-0.67)	0.67 (0.63-0.71)	0.63 (0.59-0.67)
Antiseptics - Mouth/Throat	0.63 (0.57-0.68)	0.67 (0.62-0.73)	0.62 (0.54-0.67)
Anti-infective Agents - Misc.	0.61 (0.56-0.65)	0.67 (0.62-0.72)	0.60 (0.55-0.65)
Specialty Vitamins Products	0.60 (0.52-0.68)	0.67 (0.58-0.75)	0.61 (0.52-0.69)
Anti-infective Misc. - Combinations	0.64 (0.59-0.68)	0.67 (0.62-0.72)	0.62 (0.57-0.66)
Iron	0.66 (0.62-0.71)	0.67 (0.62-0.71)	0.66 (0.61-0.71)
Cephalosporins - 3rd Generation	0.62 (0.57-0.66)	0.66 (0.63-0.71)	0.61 (0.57-0.65)
Glucocorticosteroids	0.59 (0.55-0.63)	0.66 (0.63-0.70)	0.58 (0.54-0.62)
Antihistamines - Ethanolamines	0.62 (0.58-0.65)	0.66 (0.62-0.70)	0.62 (0.58-0.66)
Fluoroquinolones	0.54 (0.50-0.58)	0.66 (0.62-0.70)	0.52 (0.48-0.57)
Calcium	0.61 (0.57-0.65)	0.66 (0.62-0.71)	0.60 (0.56-0.64)
Benzodiazepines	0.61 (0.57-0.66)	0.66 (0.62-0.70)	0.61 (0.56-0.64)
Biguanides	0.67 (0.62-0.71)	0.66 (0.61-0.71)	0.65 (0.60-0.70)
Local Anesthetic Combinations	0.57 (0.53-0.62)	0.66 (0.61-0.70)	0.56 (0.52-0.61)
Ophthalmics - Misc.	0.66 (0.60-0.72)	0.66 (0.60-0.71)	0.65 (0.60-0.70)
Antacid Combinations	0.63 (0.58-0.68)	0.66 (0.61-0.70)	0.62 (0.56-0.67)
Dibenzapines	0.62 (0.56-0.68)	0.66 (0.58-0.72)	0.58 (0.52-0.63)
Nitrates	0.64 (0.61-0.68)	0.66 (0.61-0.70)	0.63 (0.59-0.67)
Insulin	0.63 (0.58-0.67)	0.66 (0.61-0.69)	0.62 (0.57-0.66)
Liquid Vehicles	0.59 (0.51-0.67)	0.65 (0.58-0.73)	0.58 (0.51-0.66)
Alternative Medicine - M's	0.55 (0.49-0.61)	0.65 (0.59-0.71)	0.59 (0.52-0.64)

Continued on next page

Supplementary Table 2. Mean (95% confidence interval) area under the ROC curve for predicting medication usage, grouped by pharmaceutical subclass, using the baseline, methylation data, and genotype data. Confidence intervals determined using bootstrapping.

Pharmaceutical Subclass	Baseline	Methylation	Genotypes
5-HT3 Receptor Antagonists	0.57 (0.53-0.60)	0.65 (0.61-0.69)	0.55 (0.51-0.60)
Antiperistaltic Agents	0.55 (0.48-0.62)	0.65 (0.57-0.73)	0.51 (0.44-0.58)
Analgesics Other	0.59 (0.55-0.63)	0.65 (0.61-0.68)	0.58 (0.54-0.62)
Carbohydrates	0.61 (0.57-0.65)	0.65 (0.61-0.69)	0.59 (0.54-0.64)
Laxatives - Miscellaneous	0.61 (0.57-0.65)	0.65 (0.61-0.68)	0.59 (0.55-0.64)
Alpha-Beta Blockers	0.57 (0.52-0.61)	0.65 (0.60-0.69)	0.57 (0.53-0.61)
Saline Laxatives	0.59 (0.54-0.63)	0.64 (0.60-0.70)	0.58 (0.54-0.62)
Potassium	0.59 (0.55-0.63)	0.64 (0.60-0.68)	0.58 (0.54-0.62)
Stimulant Laxatives	0.60 (0.56-0.64)	0.64 (0.60-0.68)	0.60 (0.56-0.64)
Urinary Anti-infectives	0.60 (0.53-0.67)	0.64 (0.55-0.71)	0.51 (0.42-0.58)
Calcium Channel Blockers	0.59 (0.56-0.63)	0.64 (0.60-0.67)	0.60 (0.56-0.63)
Glycopeptides	0.60 (0.56-0.65)	0.64 (0.59-0.68)	0.59 (0.54-0.63)
Magnesium	0.58 (0.54-0.62)	0.64 (0.60-0.67)	0.57 (0.53-0.61)
Heparins And Heparinoid-Like Agents	0.62 (0.58-0.65)	0.64 (0.60-0.67)	0.61 (0.57-0.64)
Bicarbonates	0.60 (0.53-0.67)	0.63 (0.56-0.70)	0.61 (0.53-0.68)
Electrolyte Mixtures	0.58 (0.55-0.62)	0.63 (0.60-0.67)	0.57 (0.54-0.61)
Thyroid Hormones	0.64 (0.58-0.69)	0.63 (0.58-0.68)	0.63 (0.58-0.68)
Antihypertensive Combinations	0.64 (0.56-0.70)	0.63 (0.57-0.71)	0.59 (0.50-0.67)
Folic Acid/Folates	0.61 (0.54-0.67)	0.63 (0.58-0.69)	0.60 (0.55-0.66)
Diagnostic Radiopharmaceuticals	0.58 (0.54-0.62)	0.63 (0.60-0.67)	0.57 (0.52-0.61)
Surfactant Laxatives	0.60 (0.57-0.64)	0.63 (0.58-0.66)	0.60 (0.56-0.64)
Thiazides and Thiazide-Like Diuretics	0.65 (0.61-0.71)	0.63 (0.58-0.68)	0.63 (0.58-0.67)
Expectorants	0.57 (0.47-0.65)	0.63 (0.54-0.72)	0.44 (0.36-0.52)
Platelet Aggregation Inhibitors	0.64 (0.59-0.68)	0.63 (0.58-0.67)	0.62 (0.58-0.67)
Antiflatulents	0.56 (0.50-0.61)	0.63 (0.58-0.68)	0.54 (0.48-0.60)

Continued on next page

Supplementary Table 2. Mean (95% confidence interval) area under the ROC curve for predicting medication usage, grouped by pharmaceutical subclass, using the baseline, methylation data, and genotype data. Confidence intervals determined using bootstrapping.

Pharmaceutical Subclass	Baseline	Methylation	Genotypes
Vasopressors	0.57 (0.53-0.61)	0.62 (0.59-0.65)	0.55 (0.52-0.60)
Opioid Antagonists	0.60 (0.54-0.65)	0.62 (0.58-0.67)	0.60 (0.54-0.65)
Antibiotics - Topical	0.55 (0.50-0.60)	0.62 (0.58-0.67)	0.50 (0.45-0.56)
Antidiarrheal/Probiotic Agents - Misc.	0.56 (0.49-0.63)	0.62 (0.55-0.69)	0.51 (0.43-0.59)
Serotonin-Norepinephrine Reuptake Inhibitors (S...	0.68 (0.61-0.74)	0.62 (0.54-0.70)	0.66 (0.58-0.73)
Phenothiazines	0.52 (0.46-0.56)	0.62 (0.56-0.66)	0.46 (0.41-0.50)
Beta Blockers Cardio-Selective	0.54 (0.51-0.58)	0.61 (0.58-0.65)	0.53 (0.49-0.57)
Misc. Nutritional Substances	0.55 (0.49-0.59)	0.61 (0.56-0.67)	0.54 (0.49-0.59)
Alpha-2 Receptor Antagonists (Tetracyclics)	0.66 (0.56-0.75)	0.61 (0.51-0.70)	0.67 (0.58-0.75)
Bronchodilators - Anticholinergics	0.63 (0.58-0.67)	0.61 (0.56-0.67)	0.61 (0.56-0.66)
Tetracyclines	0.51 (0.45-0.59)	0.61 (0.56-0.67)	0.40 (0.35-0.46)
Antacids - Calcium Salts	0.58 (0.53-0.63)	0.61 (0.56-0.66)	0.55 (0.48-0.60)
Penicillin Combinations	0.57 (0.52-0.61)	0.61 (0.56-0.65)	0.55 (0.51-0.60)
Gout Agents	0.65 (0.60-0.69)	0.61 (0.54-0.66)	0.63 (0.57-0.68)
Radiographic Contrast Media	0.61 (0.57-0.64)	0.61 (0.57-0.64)	0.60 (0.56-0.63)
Sodium	0.47 (0.43-0.52)	0.61 (0.56-0.65)	0.54 (0.48-0.59)
Diagnostic Tests	0.60 (0.55-0.65)	0.61 (0.55-0.66)	0.57 (0.52-0.63)
Sympathomimetics	0.59 (0.55-0.62)	0.61 (0.57-0.65)	0.57 (0.53-0.61)
Antiarrhythmics Type III	0.56 (0.48-0.62)	0.60 (0.53-0.67)	0.53 (0.46-0.61)
Antihistamines-Topical	0.48 (0.41-0.57)	0.60 (0.53-0.68)	0.39 (0.31-0.47)
Antidotes and Specific Antagonists	0.39 (0.33-0.46)	0.60 (0.54-0.67)	0.59 (0.52-0.65)
Coumarin Anticoagulants	0.62 (0.57-0.68)	0.60 (0.55-0.66)	0.58 (0.52-0.62)
Bacterial Vaccines	0.56 (0.51-0.62)	0.60 (0.55-0.65)	0.56 (0.52-0.61)
Genitourinary Irrigants	0.41 (0.36-0.46)	0.60 (0.55-0.65)	0.54 (0.49-0.59)
Anesthetics Topical Oral	0.63 (0.57-0.69)	0.60 (0.53-0.66)	0.60 (0.54-0.67)

Continued on next page

Supplementary Table 2. Mean (95% confidence interval) area under the ROC curve for predicting medication usage, grouped by pharmaceutical subclass, using the baseline, methylation data, and genotype data. Confidence intervals determined using bootstrapping.

Pharmaceutical Subclass	Baseline	Methylation	Genotypes
Cobalamins	0.60 (0.54-0.65)	0.60 (0.55-0.66)	0.57 (0.52-0.63)
Posterior Pituitary Hormones	0.56 (0.50-0.62)	0.59 (0.53-0.66)	0.50 (0.43-0.56)
Gastrointestinal Stimulants	0.52 (0.47-0.57)	0.59 (0.55-0.65)	0.53 (0.48-0.57)
Antiadrenergic Antihypertensives	0.55 (0.48-0.60)	0.59 (0.54-0.65)	0.49 (0.43-0.54)
Angiotensin II Receptor Antagonists	0.61 (0.56-0.66)	0.59 (0.55-0.63)	0.58 (0.54-0.62)
Antitussives	0.50 (0.43-0.56)	0.59 (0.53-0.65)	0.50 (0.44-0.55)
B-Complex Vitamins	0.49 (0.41-0.57)	0.59 (0.47-0.67)	0.48 (0.40-0.56)
Cephalosporins - 1st Generation	0.55 (0.51-0.59)	0.59 (0.55-0.63)	0.53 (0.49-0.58)
Diagnostic Drugs	0.59 (0.54-0.63)	0.59 (0.54-0.63)	0.59 (0.54-0.63)
Potassium Sparing Diuretics	0.60 (0.53-0.66)	0.58 (0.52-0.66)	0.57 (0.50-0.64)
Selective Serotonin Reuptake Inhibitors (SSRIs)	0.59 (0.55-0.64)	0.58 (0.53-0.64)	0.57 (0.52-0.62)
Anesthetics - Misc.	0.53 (0.49-0.56)	0.58 (0.55-0.62)	0.52 (0.49-0.55)
Irrigation Solutions	0.58 (0.50-0.64)	0.58 (0.51-0.64)	0.55 (0.47-0.62)
Lozenges	0.58 (0.50-0.65)	0.58 (0.49-0.66)	0.51 (0.43-0.60)
Aminoglycosides	0.55 (0.48-0.62)	0.58 (0.50-0.64)	0.45 (0.38-0.54)
Non-Barbiturate Hypnotics	0.55 (0.51-0.59)	0.57 (0.53-0.61)	0.53 (0.49-0.57)
Ophthalmic Anti-infectives	0.61 (0.56-0.66)	0.57 (0.53-0.63)	0.61 (0.54-0.66)
Antiarrhythmics Type I-B	0.54 (0.50-0.58)	0.57 (0.54-0.62)	0.51 (0.48-0.55)
Oil Soluble Vitamins	0.57 (0.53-0.61)	0.57 (0.53-0.61)	0.59 (0.56-0.64)
Direct Factor Xa Inhibitors	0.58 (0.52-0.64)	0.57 (0.51-0.63)	0.57 (0.51-0.63)
H-2 Antagonists	0.52 (0.48-0.57)	0.57 (0.53-0.62)	0.50 (0.47-0.55)
Multivitamins	0.51 (0.46-0.55)	0.57 (0.52-0.62)	0.52 (0.47-0.57)
Hemostatics - Topical	0.57 (0.50-0.63)	0.57 (0.50-0.64)	0.53 (0.47-0.58)
Artificial Tears and Lubricants	0.56 (0.48-0.63)	0.57 (0.51-0.64)	0.58 (0.51-0.65)
Anti-inflammatory Agents - Topical	0.58 (0.52-0.65)	0.57 (0.51-0.64)	0.58 (0.51-0.64)

Continued on next page

Supplementary Table 2. Mean (95% confidence interval) area under the ROC curve for predicting medication usage, grouped by pharmaceutical subclass, using the baseline, methylation data, and genotype data. Confidence intervals determined using bootstrapping.

Pharmaceutical Subclass	Baseline	Methylation	Genotypes
Opioid Agonists	0.58 (0.53-0.62)	0.57 (0.53-0.61)	0.55 (0.51-0.60)
Leukotriene Modulators	0.55 (0.46-0.63)	0.57 (0.48-0.66)	0.50 (0.40-0.57)
Antianxiety Agents - Misc.	0.58 (0.52-0.65)	0.57 (0.50-0.64)	0.53 (0.46-0.60)
Local Anesthetics - Amides	0.58 (0.53-0.62)	0.57 (0.53-0.61)	0.56 (0.51-0.60)
Water Soluble Vitamins	0.57 (0.52-0.62)	0.57 (0.52-0.62)	0.56 (0.51-0.61)
Nondepolarizing Muscle Relaxants	0.56 (0.52-0.60)	0.57 (0.52-0.61)	0.54 (0.51-0.58)
Urinary Antispasmodic - Antimuscarinics (Antich...	0.59 (0.51-0.66)	0.57 (0.49-0.64)	0.59 (0.51-0.66)
Bulk Laxatives	0.53 (0.46-0.61)	0.56 (0.50-0.64)	0.53 (0.45-0.61)
Antiemetics - Anticholinergic	0.53 (0.46-0.60)	0.56 (0.50-0.63)	0.45 (0.39-0.51)
Aminopenicillins	0.55 (0.49-0.60)	0.56 (0.51-0.62)	0.54 (0.48-0.59)
Serotonin Modulators	0.53 (0.48-0.58)	0.56 (0.51-0.61)	0.53 (0.48-0.59)
Viral Vaccines	0.56 (0.51-0.61)	0.56 (0.51-0.60)	0.52 (0.47-0.57)
Laxative Combinations	0.57 (0.52-0.63)	0.56 (0.50-0.61)	0.55 (0.50-0.61)
Antimyasthenic/Cholinergic Agents	0.54 (0.50-0.59)	0.56 (0.50-0.60)	0.52 (0.46-0.58)
Azithromycin	0.59 (0.54-0.63)	0.55 (0.51-0.60)	0.57 (0.53-0.63)
Local Anesthetics - Topical	0.54 (0.49-0.58)	0.55 (0.51-0.60)	0.52 (0.47-0.56)
ACE Inhibitors	0.51 (0.47-0.55)	0.55 (0.50-0.60)	0.48 (0.43-0.52)
Herpes Agents	0.53 (0.45-0.59)	0.55 (0.48-0.62)	0.48 (0.41-0.55)
Influenza Agents	0.51 (0.44-0.58)	0.54 (0.48-0.61)	0.45 (0.38-0.53)
Beta Blockers Non-Selective	0.52 (0.44-0.59)	0.54 (0.45-0.64)	0.59 (0.50-0.69)
Sulfonylureas	0.59 (0.51-0.66)	0.54 (0.47-0.62)	0.61 (0.55-0.69)
Nasal Steroids	0.56 (0.51-0.60)	0.54 (0.50-0.59)	0.51 (0.46-0.57)
Antispasmodics	0.56 (0.52-0.60)	0.54 (0.50-0.58)	0.56 (0.52-0.59)
Cough/Cold/Allergy Combinations	0.58 (0.52-0.65)	0.54 (0.48-0.61)	0.55 (0.49-0.63)
Cephalosporins - 2nd Generation	0.52 (0.44-0.59)	0.54 (0.47-0.62)	0.49 (0.42-0.56)

Continued on next page

Supplementary Table 2. Mean (95% confidence interval) area under the ROC curve for predicting medication usage, grouped by pharmaceutical subclass, using the baseline, methylation data, and genotype data. Confidence intervals determined using bootstrapping.

Pharmaceutical Subclass	Baseline	Methylation	Genotypes
Opioid Combinations	0.53 (0.49-0.57)	0.54 (0.50-0.58)	0.50 (0.46-0.53)
Anticonvulsants - Misc.	0.54 (0.49-0.58)	0.54 (0.50-0.58)	0.51 (0.47-0.55)
Multiple Vitamins w/ Minerals	0.58 (0.51-0.65)	0.54 (0.47-0.59)	0.54 (0.49-0.60)
Protamine	0.53 (0.46-0.61)	0.54 (0.44-0.64)	0.50 (0.39-0.59)
Lincosamides	0.52 (0.46-0.59)	0.53 (0.47-0.59)	0.52 (0.44-0.59)
Hemostatics - Systemic	0.46 (0.37-0.55)	0.53 (0.44-0.62)	0.54 (0.47-0.62)
Antihistamines - Non-Sedating	0.54 (0.47-0.60)	0.52 (0.46-0.57)	0.53 (0.46-0.58)
Acne Products	0.59 (0.49-0.66)	0.50 (0.41-0.60)	0.41 (0.33-0.50)
Miscellaneous Contrast Media	0.49 (0.46-0.53)	0.50 (0.46-0.54)	0.53 (0.49-0.57)
Nonsteroidal Anti-inflammatory Agents (NSAIDs)	0.52 (0.49-0.57)	0.49 (0.45-0.53)	0.53 (0.48-0.57)
Toxoid Combinations	0.55 (0.49-0.63)	0.49 (0.42-0.57)	0.55 (0.48-0.63)
Corticosteroids - Topical	0.51 (0.46-0.56)	0.49 (0.43-0.53)	0.43 (0.38-0.47)
Tricyclic Agents	0.53 (0.44-0.61)	0.48 (0.39-0.56)	0.58 (0.51-0.65)
Central Muscle Relaxants	0.52 (0.48-0.57)	0.48 (0.43-0.52)	0.48 (0.43-0.53)
Depolarizing Muscle Relaxants	0.53 (0.47-0.58)	0.46 (0.40-0.51)	0.49 (0.43-0.54)
Antifungals - Topical	0.50 (0.44-0.55)	0.45 (0.40-0.50)	0.48 (0.43-0.54)
Cardiac Glycosides	0.52 (0.42-0.60)	0.43 (0.33-0.52)	0.54 (0.46-0.63)
Alternative Medicine - C's	0.53 (0.47-0.60)	0.37 (0.30-0.44)	0.51 (0.45-0.60)

Supplementary Table 3. Mean (95% confidence interval) R^2 for predicting the most recent lab result using the baseline, methylation data, and genotype data. Confidence intervals determined using bootstrapping. Activated Partial Thromboplastin Time (APTT); Point of care (POC); Pulmonary function test (PFT); Forced expiratory volume in 1 second (FEV1)

Lab Test	Baseline	Methylation	Genotypes
Troponin	0.65 (0.53-0.74)	0.62 (0.51-0.72)	0.60 (0.50-0.70)

Continued on next page

Supplementary Table 3. Mean (95% confidence interval) R^2 for predicting the most recent lab result using the baseline, methylation data, and genotype data. Confidence intervals determined using bootstrapping. Activated Partial Thromboplastin Time (APTT); Point of care (POC); Pulmonary function test (PFT); Forced expiratory volume in 1 second (FEV1)

Lab Test	Baseline	Methylation	Genotypes
Creatinine	0.08 (0.01-0.13)	0.43 (0.38-0.47)	0.09 (0.04-0.13)
Troponin interpretation	0.46 (0.31-0.62)	0.41 (0.26-0.54)	0.33 (0.20-0.48)
Urea nitrogen	0.01 (-0.04-0.05)	0.40 (0.35-0.45)	0.03 (-0.00-0.05)
Absolute eosinophil count	-0.06 (-0.10–0.03)	0.33 (0.27-0.40)	-0.01 (-0.02-0.00)
Hemoglobin	0.10 (0.04-0.15)	0.28 (0.23-0.33)	0.10 (0.05-0.14)
Neutrophil percent (auto)	0.23 (0.12-0.32)	0.26 (0.18-0.33)	0.22 (0.13-0.30)
PFT FEV1 (pre)	0.12 (-0.18-0.36)	0.26 (0.07-0.38)	0.18 (-0.02-0.32)
Hematocrit	0.07 (0.02-0.11)	0.24 (0.20-0.28)	0.07 (0.04-0.11)
Mean corpuscular hemoglobin	0.07 (0.01-0.13)	0.20 (0.15-0.26)	0.08 (0.04-0.12)
Mean corpuscular volume	0.09 (0.03-0.14)	0.18 (0.12-0.24)	0.09 (0.04-0.13)
Absolute lymphocyte count	0.06 (-0.04-0.17)	0.17 (0.08-0.33)	0.10 (0.03-0.23)
Platelet count (auto)	-0.00 (-0.05-0.05)	0.16 (0.12-0.21)	0.02 (-0.00-0.04)
Absolute neutrophil count	0.08 (0.01-0.13)	0.15 (0.10-0.20)	0.08 (0.04-0.11)
Albumin	0.07 (0.01-0.13)	0.14 (0.08-0.18)	0.08 (0.04-0.12)
Chloride	-0.01 (-0.05-0.03)	0.13 (0.09-0.18)	0.01 (-0.01-0.03)
Absolute immature granulocyte count	-0.09 (-0.25-0.01)	0.13 (0.05-0.20)	-0.00 (-0.04-0.02)
Absolute monocyte count	0.08 (0.00-0.14)	0.12 (0.06-0.17)	0.07 (0.01-0.13)
White blood cell count	-0.01 (-0.07-0.04)	0.11 (0.05-0.19)	0.02 (-0.01-0.05)
Neutrophils absolute (prelim).	0.07 (0.01-0.13)	0.11 (0.06-0.18)	0.08 (0.03-0.13)
HgbA1C	-0.07 (-0.15-0.01)	0.11 (0.06-0.17)	-0.01 (-0.04-0.01)
Total protein	0.09 (0.03-0.14)	0.11 (0.06-0.16)	0.08 (0.04-0.12)
Sodium	-0.00 (-0.04-0.04)	0.10 (0.07-0.14)	0.03 (0.00-0.05)
Ferritin	-0.21 (-0.39–0.07)	0.10 (0.04-0.15)	-0.02 (-0.05–0.00)
Sedimentation rate erythrocyte	-0.10 (-0.38-0.12)	0.10 (-0.01-0.18)	0.08 (-0.01-0.17)

Continued on next page

Supplementary Table 3. Mean (95% confidence interval) R^2 for predicting the most recent lab result using the baseline, methylation data, and genotype data. Confidence intervals determined using bootstrapping. Activated Partial Thromboplastin Time (APTT); Point of care (POC); Pulmonary function test (PFT); Forced expiratory volume in 1 second (FEV1)

Lab Test	Baseline	Methylation	Genotypes
Iron binding capacity	-0.10 (-0.19–0.00)	0.09 (0.03-0.14)	-0.00 (-0.03-0.03)
Absolute basophil count	-0.10 (-0.25–0.02)	0.07 (0.05-0.11)	-0.01 (-0.02–0.00)
Glucose	-0.02 (-0.07-0.02)	0.07 (0.04-0.10)	0.01 (-0.01-0.02)
Qrs.duration	-0.04 (-0.11-0.02)	0.05 (0.00-0.08)	0.01 (-0.01-0.02)
Cholesterol HDL	0.03 (-0.07-0.12)	0.04 (-0.03-0.10)	0.06 (0.00-0.12)
Hematocrit OSL	-0.31 (-0.81-0.09)	0.04 (-0.13-0.19)	-0.05 (-0.22-0.08)
Cholesterol	-0.05 (-0.11-0.02)	0.03 (-0.02-0.08)	0.01 (-0.02-0.04)
Ventricular rate	-0.06 (-0.14-0.01)	0.03 (-0.00-0.06)	0.02 (-0.01-0.04)
Anion gap	-0.04 (-0.07–0.01)	0.02 (-0.00-0.05)	-0.00 (-0.02-0.01)
Alanine aminotransferase	-0.05 (-0.09–0.02)	0.02 (0.01-0.04)	-0.01 (-0.01-0.00)
R axis	-0.03 (-0.10-0.03)	0.01 (-0.01-0.04)	0.00 (-0.02-0.03)
Magnesium	-0.11 (-0.19–0.04)	0.01 (-0.02-0.05)	-0.00 (-0.03-0.02)
Alkaline phosphatase	-0.02 (-0.07-0.02)	0.01 (-0.01-0.03)	0.00 (-0.01-0.01)
T.axis	-0.08 (-0.19–0.01)	0.01 (-0.02-0.04)	-0.00 (-0.01-0.01)
Cholesterol LDL (calculated)	-0.10 (-0.22–0.02)	0.01 (-0.02-0.04)	-0.01 (-0.03-0.01)
Potassium	-0.03 (-0.07-0.00)	0.01 (-0.01-0.02)	-0.01 (-0.02-0.01)
Aspartate aminotransferase	-0.02 (-0.07-0.03)	0.01 (-0.01-0.03)	0.00 (-0.02-0.02)
International normalized ratio (INR)	-0.02 (-0.06-0.02)	0.01 (-0.01-0.03)	0.00 (-0.02-0.02)
Bilirubin total	-0.05 (-0.16–0.01)	0.01 (-0.02-0.02)	-0.00 (-0.02-0.00)
Prothrombin time	-0.02 (-0.10-0.02)	0.01 (-0.01-0.03)	0.00 (-0.01-0.02)
QT interval	-0.07 (-0.12–0.01)	0.00 (-0.02-0.02)	0.01 (-0.01-0.02)
Glucose (POC)	-0.08 (-0.21-0.02)	0.00 (-0.03-0.03)	0.00 (-0.04-0.04)
X saturation	-0.18 (-0.30–0.06)	0.00 (-0.04-0.04)	-0.01 (-0.03–0.00)
PR interval	-0.05 (-0.15-0.03)	0.00 (-0.04-0.03)	-0.00 (-0.03-0.02)

Continued on next page

Supplementary Table 3. Mean (95% confidence interval) R^2 for predicting the most recent lab result using the baseline, methylation data, and genotype data. Confidence intervals determined using bootstrapping. Activated Partial Thromboplastin Time (APTT); Point of care (POC); Pulmonary function test (PFT); Forced expiratory volume in 1 second (FEV1)

Lab Test	Baseline	Methylation	Genotypes
Brain natriuretic peptide (BNP)	-0.51 (-1.38–0.03)	0.00 (-0.08-0.05)	0.03 (-0.07-0.11)
Glucose whole blood	-0.24 (-0.49–0.09)	-0.00 (-0.03-0.03)	-0.03 (-0.08-0.00)
Atrial rate	-0.05 (-0.13-0.01)	-0.00 (-0.02-0.01)	-0.00 (-0.02-0.02)
P axis	-0.04 (-0.10-0.01)	-0.00 (-0.02-0.02)	-0.00 (-0.02-0.02)
QtC calculation (bezet)	-0.07 (-0.14–0.01)	-0.00 (-0.02-0.02)	-0.00 (-0.02-0.01)
PFT FEV1 (pre) (percent ref)	-0.31 (-0.58–0.06)	-0.00 (-0.06-0.04)	-0.03 (-0.07–0.01)
Blood lactate	-0.29 (-0.64-0.02)	-0.01 (-0.08-0.05)	0.00 (-0.06-0.05)
APTT	-0.05 (-0.12–0.01)	-0.01 (-0.02-0.00)	-0.01 (-0.03-0.00)
Triglycerides	-0.13 (-0.23–0.05)	-0.01 (-0.03-0.01)	-0.01 (-0.03–0.00)
Thyroid stimulating hormone (TSH)	-0.13 (-0.37–0.05)	-0.01 (-0.04–0.01)	-0.02 (-0.04–0.00)
Calcium	-0.05 (-0.08–0.02)	-0.01 (-0.02–0.00)	-0.02 (-0.03–0.00)
Iron	-0.13 (-0.26–0.00)	-0.02 (-0.06-0.03)	-0.02 (-0.05-0.01)
Urea nitrogen (OSL)	-0.61 (-1.93-0.00)	-0.04 (-0.17-0.05)	-0.04 (-0.22-0.03)
Bilirubin conjugated	-0.57 (-1.25–0.17)	-0.06 (-0.18–0.00)	-0.06 (-0.17–0.03)
C reactive protein (CRP)	-0.44 (-1.14–0.13)	-0.06 (-0.24–0.01)	-0.01 (-0.08-0.02)
Left ventricular ejection fraction	-1.80 (-3.29–0.91)	-0.06 (-0.21-0.01)	-0.07 (-0.28-0.03)
Hemoglobin (OSL)	-2.66 (-12.80–0.23)	-0.07 (-0.37–0.02)	-0.09 (-0.45–0.02)
Chloride (OSL)	-1.96 (-4.43–0.46)	-0.19 (-0.70-0.03)	-0.17 (-0.65-0.04)
Sodium (OSL)	-304.86 (-2851.16–0.13)	-2.03 (-16.23–0.02)	-1.57 (-16.42–0.01)
Potassium (OSL).	-178.77 (-828.09–0.14)	-2.33 (-8.10–0.02)	-2.42 (-8.14–0.02)

Supplementary Table 4. Mean (95% confidence interval) area under the ROC curve for predicting patient diagnoses, grouped into Phecode phenotypes, using the baseline, methylation data, and genotype data. Confidence intervals determined using bootstrapping.

Phecode Phenotype	Baseline	Methylation	Genotypes
End stage renal disease	0.72 (0.68-0.76)	0.90 (0.87-0.92)	0.72 (0.67-0.77)
Renal dialysis	0.73 (0.67-0.77)	0.88 (0.85-0.91)	0.72 (0.68-0.76)
Cirrhosis of liver without mention of alcohol	0.69 (0.63-0.76)	0.86 (0.82-0.89)	0.68 (0.62-0.75)
Morbid obesity	0.86 (0.79-0.92)	0.85 (0.78-0.90)	0.86 (0.79-0.91)
Cancer of prostate	0.82 (0.76-0.86)	0.85 (0.78-0.89)	0.78 (0.73-0.83)
Hyperplasia of prostate	0.85 (0.81-0.88)	0.84 (0.82-0.87)	0.84 (0.81-0.88)
Neutropenia	0.60 (0.51-0.67)	0.84 (0.79-0.88)	0.42 (0.36-0.49)
Obesity	0.84 (0.80-0.87)	0.84 (0.80-0.87)	0.84 (0.80-0.87)
Menopausal and postmenopausal disorders	0.84 (0.80-0.87)	0.83 (0.79-0.87)	0.82 (0.78-0.86)
Tobacco use disorder	0.79 (0.72-0.84)	0.83 (0.77-0.88)	0.77 (0.72-0.82)
Noninflammatory female genital disorders	0.80 (0.77-0.83)	0.82 (0.78-0.85)	0.78 (0.74-0.81)
Chronic renal failure [CKD]	0.63 (0.59-0.68)	0.82 (0.78-0.85)	0.63 (0.58-0.66)
Immunity deficiency	0.76 (0.70-0.80)	0.82 (0.77-0.86)	0.75 (0.70-0.80)
Poisoning by primarily systemic agents	0.63 (0.55-0.71)	0.82 (0.74-0.88)	0.55 (0.46-0.62)
Kidney replaced by transpant	0.74 (0.69-0.79)	0.81 (0.77-0.86)	0.72 (0.67-0.78)
Decreased white blood cell count	0.66 (0.59-0.71)	0.81 (0.76-0.86)	0.62 (0.56-0.68)
Anemia in chronic kidney disease	0.71 (0.65-0.76)	0.81 (0.77-0.85)	0.71 (0.65-0.77)
Portal hypertension	0.70 (0.63-0.76)	0.80 (0.73-0.86)	0.68 (0.60-0.75)
Disorders involving the immune mechanism	0.76 (0.72-0.80)	0.80 (0.76-0.84)	0.75 (0.70-0.79)
Hypertensive chronic kidney disease	0.62 (0.57-0.66)	0.80 (0.76-0.84)	0.59 (0.55-0.64)
Renal failure	0.62 (0.58-0.66)	0.80 (0.76-0.83)	0.60 (0.57-0.64)
Disorders resulting from impaired renal function	0.68 (0.62-0.74)	0.79 (0.74-0.84)	0.68 (0.61-0.73)
Antineoplastic and immunosuppressive drugs caus...	0.67 (0.59-0.75)	0.79 (0.71-0.87)	0.48 (0.41-0.58)
Anemia of chronic disease	0.70 (0.65-0.73)	0.79 (0.76-0.82)	0.70 (0.65-0.74)
Overweight, obesity and other hyperalimentation	0.79 (0.75-0.83)	0.79 (0.75-0.83)	0.79 (0.75-0.83)

Continued on next page

Supplementary Table 4. Mean (95% confidence interval) area under the ROC curve for predicting patient diagnoses, grouped into Phecode phenotypes, using the baseline, methylation data, and genotype data. Confidence intervals determined using bootstrapping.

Phecode Phenotype	Baseline	Methylation	Genotypes
Liver abscess and sequelae of chronic liver dis...	0.72 (0.64-0.79)	0.78 (0.72-0.84)	0.66 (0.58-0.73)
Osteoarthrosis	0.78 (0.73-0.82)	0.78 (0.73-0.82)	0.77 (0.73-0.81)
Liver replaced by transplant	0.68 (0.61-0.77)	0.76 (0.70-0.82)	0.64 (0.56-0.74)
Secondary hyperparathyroidism (of renal origin)	0.67 (0.60-0.73)	0.76 (0.71-0.82)	0.65 (0.57-0.71)
Osteoarthrosis NOS	0.77 (0.72-0.82)	0.76 (0.71-0.81)	0.76 (0.72-0.81)
Disorders of phosphorus metabolism	0.73 (0.65-0.80)	0.76 (0.69-0.82)	0.71 (0.63-0.78)
Abnormal involuntary movements	0.39 (0.31-0.49)	0.76 (0.68-0.82)	0.54 (0.44-0.62)
Fluid overload	0.66 (0.61-0.73)	0.76 (0.70-0.81)	0.68 (0.62-0.75)
Erectile dysfunction [ED]	0.72 (0.65-0.78)	0.76 (0.70-0.82)	0.72 (0.64-0.77)
Osteoarthritis; localized	0.76 (0.71-0.82)	0.76 (0.70-0.82)	0.74 (0.68-0.80)
Coagulation defects	0.68 (0.62-0.75)	0.74 (0.67-0.80)	0.64 (0.56-0.72)
Acid-base balance disorder	0.66 (0.60-0.72)	0.73 (0.68-0.79)	0.64 (0.58-0.71)
Osteoarthrosis, localized, primary	0.75 (0.69-0.81)	0.73 (0.66-0.78)	0.75 (0.69-0.80)
Respiratory failure, insufficiency, arrest	0.61 (0.55-0.67)	0.73 (0.68-0.78)	0.56 (0.48-0.64)
Ascites (non malignant)	0.73 (0.66-0.80)	0.73 (0.65-0.79)	0.73 (0.66-0.79)
Degenerative skin conditions and other dermatoses	0.72 (0.67-0.76)	0.73 (0.68-0.78)	0.70 (0.65-0.75)
Hypertensive heart and/or renal disease	0.56 (0.51-0.61)	0.72 (0.69-0.77)	0.58 (0.53-0.63)
Altered mental status	0.62 (0.53-0.71)	0.72 (0.64-0.81)	0.59 (0.50-0.68)
Arthropathy NOS	0.72 (0.66-0.78)	0.72 (0.66-0.78)	0.71 (0.65-0.77)
Cataract	0.73 (0.68-0.77)	0.72 (0.67-0.76)	0.72 (0.67-0.76)
Nephritis and nephropathy in diseases classifie...	0.69 (0.61-0.76)	0.71 (0.64-0.78)	0.70 (0.63-0.77)
Family history	0.64 (0.56-0.71)	0.71 (0.65-0.79)	0.66 (0.56-0.75)
Purpura and other hemorrhagic conditions	0.61 (0.53-0.68)	0.71 (0.65-0.78)	0.61 (0.54-0.67)
Chronic liver disease and cirrhosis	0.61 (0.55-0.68)	0.71 (0.65-0.77)	0.62 (0.55-0.68)
Osteoporosis NOS	0.72 (0.64-0.79)	0.71 (0.64-0.78)	0.71 (0.64-0.78)

Continued on next page

Supplementary Table 4. Mean (95% confidence interval) area under the ROC curve for predicting patient diagnoses, grouped into Phecode phenotypes, using the baseline, methylation data, and genotype data. Confidence intervals determined using bootstrapping.

Phecode Phenotype	Baseline	Methylation	Genotypes
Splenomegaly	0.65 (0.59-0.71)	0.71 (0.63-0.77)	0.65 (0.57-0.73)
Hyperpotassemia	0.66 (0.61-0.71)	0.71 (0.66-0.75)	0.64 (0.59-0.69)
Disorders of calcium/phosphorus metabolism	0.67 (0.58-0.73)	0.71 (0.65-0.76)	0.65 (0.59-0.72)
Other disorders of metabolism	0.57 (0.48-0.65)	0.71 (0.63-0.77)	0.52 (0.43-0.59)
Viral infection	0.68 (0.61-0.75)	0.70 (0.64-0.77)	0.66 (0.58-0.73)
Acute renal failure	0.60 (0.55-0.64)	0.70 (0.66-0.74)	0.57 (0.53-0.62)
Essential hypertension	0.67 (0.64-0.71)	0.70 (0.66-0.74)	0.67 (0.64-0.70)
Seborrheic keratosis	0.70 (0.65-0.75)	0.70 (0.65-0.75)	0.68 (0.63-0.73)
Diverticulosis and diverticulitis	0.72 (0.65-0.78)	0.70 (0.64-0.75)	0.68 (0.60-0.75)
Hyperlipidemia	0.70 (0.66-0.74)	0.70 (0.66-0.73)	0.70 (0.66-0.74)
Thrombocytopenia	0.62 (0.56-0.69)	0.70 (0.64-0.76)	0.63 (0.56-0.69)
Type 2 diabetes	0.66 (0.62-0.71)	0.69 (0.65-0.73)	0.65 (0.62-0.69)
Other ill-defined and unknown causes of morbidi...	0.72 (0.65-0.78)	0.69 (0.63-0.77)	0.68 (0.60-0.75)
Osteoporosis	0.72 (0.65-0.78)	0.69 (0.62-0.76)	0.68 (0.61-0.76)
Poisoning by hormones and synthetic substitutes	0.60 (0.51-0.70)	0.69 (0.61-0.77)	0.56 (0.47-0.65)
Renal failure NOS	0.61 (0.55-0.69)	0.69 (0.60-0.76)	0.56 (0.48-0.62)
Type 2 diabetes with neurological manifestations	0.67 (0.60-0.74)	0.69 (0.62-0.76)	0.65 (0.56-0.73)
Polyneuropathy in diabetes	0.64 (0.57-0.71)	0.69 (0.60-0.77)	0.62 (0.53-0.70)
Respiratory failure	0.65 (0.58-0.72)	0.69 (0.61-0.76)	0.61 (0.53-0.68)
Other immunological findings	0.65 (0.56-0.73)	0.69 (0.61-0.77)	0.62 (0.54-0.71)
Deep vein thrombosis [DVT]	0.67 (0.59-0.74)	0.69 (0.63-0.76)	0.61 (0.53-0.69)
Carditis	0.60 (0.51-0.69)	0.69 (0.61-0.76)	0.41 (0.34-0.50)
Other disorders of the kidney and ureters	0.56 (0.51-0.60)	0.69 (0.65-0.72)	0.54 (0.49-0.60)
Skin cancer	0.71 (0.66-0.75)	0.69 (0.63-0.74)	0.69 (0.64-0.75)
Other non-epithelial cancer of skin	0.71 (0.65-0.76)	0.68 (0.63-0.74)	0.68 (0.64-0.74)

Continued on next page

Supplementary Table 4. Mean (95% confidence interval) area under the ROC curve for predicting patient diagnoses, grouped into Phecode phenotypes, using the baseline, methylation data, and genotype data. Confidence intervals determined using bootstrapping.

Phecode Phenotype	Baseline	Methylation	Genotypes
Cardiac pacemaker/device in situ	0.43 (0.36-0.51)	0.68 (0.62-0.75)	0.61 (0.53-0.69)
Type 2 diabetes with renal manifestations	0.63 (0.57-0.68)	0.68 (0.62-0.74)	0.65 (0.58-0.71)
Ischemic Heart Disease	0.68 (0.64-0.72)	0.68 (0.64-0.72)	0.67 (0.64-0.71)
Protein-calorie malnutrition	0.67 (0.59-0.73)	0.68 (0.62-0.74)	0.65 (0.59-0.71)
Nephritis and nephropathy without mention of gl...	0.59 (0.52-0.66)	0.68 (0.63-0.74)	0.61 (0.54-0.67)
Coronary atherosclerosis	0.68 (0.65-0.72)	0.68 (0.65-0.72)	0.68 (0.64-0.71)
Disorders of diaphragm	0.66 (0.59-0.73)	0.68 (0.60-0.75)	0.64 (0.56-0.71)
Myocardial infarction	0.66 (0.60-0.72)	0.68 (0.63-0.73)	0.65 (0.60-0.71)
Other and unspecified coagulation defects	0.59 (0.52-0.67)	0.68 (0.60-0.75)	0.50 (0.43-0.58)
Intestinal infection	0.66 (0.58-0.74)	0.68 (0.60-0.75)	0.62 (0.52-0.72)
Chronic Kidney Disease, Stage IV	0.55 (0.48-0.62)	0.68 (0.61-0.75)	0.49 (0.43-0.55)
Sensorineural hearing loss	0.71 (0.62-0.79)	0.68 (0.58-0.77)	0.67 (0.58-0.76)
Other arthropathies	0.70 (0.64-0.76)	0.68 (0.61-0.74)	0.69 (0.63-0.76)
Diverticulosis	0.71 (0.62-0.79)	0.68 (0.59-0.77)	0.70 (0.60-0.78)
Pneumonia	0.67 (0.62-0.73)	0.67 (0.63-0.72)	0.66 (0.60-0.71)
Glaucoma	0.67 (0.61-0.74)	0.67 (0.61-0.73)	0.65 (0.59-0.72)
Postinflammatory pulmonary fibrosis	0.66 (0.57-0.75)	0.67 (0.59-0.75)	0.59 (0.51-0.68)
Hypertension	0.65 (0.61-0.69)	0.67 (0.64-0.71)	0.64 (0.60-0.68)
Sleep apnea	0.66 (0.61-0.73)	0.67 (0.61-0.72)	0.67 (0.61-0.72)
Aortic aneurysm	0.68 (0.61-0.74)	0.67 (0.58-0.75)	0.65 (0.57-0.73)
Septicemia	0.66 (0.60-0.72)	0.67 (0.63-0.73)	0.63 (0.57-0.69)
Disorders of lipoid metabolism	0.67 (0.63-0.70)	0.67 (0.63-0.71)	0.67 (0.64-0.71)
Iron deficiency anemia secondary to blood loss ...	0.51 (0.44-0.59)	0.67 (0.59-0.74)	0.49 (0.42-0.56)
Actinic keratosis	0.69 (0.62-0.75)	0.67 (0.60-0.73)	0.73 (0.67-0.79)
Other anemias	0.62 (0.58-0.66)	0.67 (0.63-0.71)	0.62 (0.58-0.66)

Continued on next page

Supplementary Table 4. Mean (95% confidence interval) area under the ROC curve for predicting patient diagnoses, grouped into Phecode phenotypes, using the baseline, methylation data, and genotype data. Confidence intervals determined using bootstrapping.

Phecode Phenotype	Baseline	Methylation	Genotypes
Abnormal movement	0.65 (0.59-0.71)	0.67 (0.61-0.73)	0.63 (0.57-0.69)
Disorders of fluid, electrolyte, and acid-base ...	0.62 (0.58-0.67)	0.67 (0.62-0.71)	0.63 (0.59-0.67)
Acidosis	0.64 (0.58-0.68)	0.67 (0.61-0.72)	0.61 (0.54-0.67)
Sepsis	0.63 (0.58-0.69)	0.67 (0.61-0.72)	0.60 (0.53-0.65)
Atherosclerosis	0.53 (0.47-0.60)	0.66 (0.61-0.73)	0.45 (0.39-0.52)
Nephritis; nephrosis; renal sclerosis	0.56 (0.49-0.63)	0.66 (0.61-0.72)	0.58 (0.51-0.63)
Osteoporosis, osteopenia and pathological fracture	0.64 (0.59-0.68)	0.66 (0.62-0.71)	0.63 (0.59-0.68)
Other disorders of intestine	0.58 (0.49-0.66)	0.66 (0.57-0.74)	0.51 (0.43-0.60)
Tachycardia NOS	0.62 (0.55-0.68)	0.66 (0.60-0.72)	0.59 (0.52-0.66)
Iron deficiency anemias, unspecified or not due...	0.67 (0.61-0.72)	0.66 (0.61-0.71)	0.70 (0.63-0.76)
Other retinal disorders	0.63 (0.55-0.71)	0.66 (0.57-0.73)	0.57 (0.49-0.64)
Effects radiation NOS	0.64 (0.55-0.71)	0.66 (0.58-0.74)	0.59 (0.52-0.69)
Diabetes mellitus	0.65 (0.61-0.69)	0.66 (0.62-0.70)	0.64 (0.61-0.68)
Other disorders of eye	0.69 (0.64-0.75)	0.66 (0.59-0.72)	0.69 (0.62-0.76)
Disorders of vitreous body	0.67 (0.60-0.75)	0.66 (0.59-0.73)	0.69 (0.62-0.76)
Emphysema	0.66 (0.58-0.73)	0.66 (0.58-0.72)	0.62 (0.54-0.71)
Senile cataract	0.69 (0.64-0.73)	0.66 (0.60-0.72)	0.67 (0.61-0.71)
Nonrheumatic aortic valve disorders	0.66 (0.61-0.71)	0.66 (0.60-0.71)	0.65 (0.60-0.70)
Hypotension	0.63 (0.58-0.69)	0.66 (0.60-0.71)	0.61 (0.55-0.66)
Electrolyte imbalance	0.64 (0.60-0.68)	0.66 (0.61-0.70)	0.62 (0.58-0.67)
Atherosclerosis of aorta	0.67 (0.62-0.72)	0.66 (0.60-0.71)	0.65 (0.60-0.70)
Sepsis and SIRS	0.65 (0.59-0.71)	0.65 (0.60-0.71)	0.63 (0.57-0.69)
Acute posthemorrhagic anemia	0.67 (0.62-0.72)	0.65 (0.59-0.70)	0.66 (0.61-0.70)
Urinary tract infection	0.62 (0.57-0.67)	0.65 (0.61-0.70)	0.62 (0.57-0.66)
Other chronic ischemic heart disease, unspecified	0.70 (0.62-0.77)	0.65 (0.58-0.72)	0.67 (0.60-0.74)

Continued on next page

Supplementary Table 4. Mean (95% confidence interval) area under the ROC curve for predicting patient diagnoses, grouped into Phecode phenotypes, using the baseline, methylation data, and genotype data. Confidence intervals determined using bootstrapping.

Phecode Phenotype	Baseline	Methylation	Genotypes
Nausea and vomiting	0.53 (0.48-0.59)	0.65 (0.61-0.69)	0.47 (0.41-0.52)
Chronic Kidney Disease, Stage III	0.58 (0.52-0.63)	0.65 (0.60-0.70)	0.55 (0.49-0.62)
Fever of unknown origin	0.63 (0.58-0.68)	0.65 (0.59-0.71)	0.63 (0.55-0.69)
Acute pain	0.59 (0.53-0.65)	0.65 (0.60-0.71)	0.58 (0.52-0.64)
Obstructive sleep apnea	0.65 (0.59-0.71)	0.65 (0.58-0.72)	0.65 (0.58-0.71)
Alcoholism	0.60 (0.49-0.70)	0.65 (0.57-0.74)	0.52 (0.41-0.62)
Secondary malignant neoplasm	0.56 (0.48-0.65)	0.65 (0.57-0.72)	0.66 (0.58-0.73)
Cystitis and urethritis	0.59 (0.53-0.66)	0.65 (0.57-0.72)	0.55 (0.47-0.63)
Angina pectoris	0.64 (0.59-0.70)	0.65 (0.58-0.70)	0.59 (0.53-0.65)
Urinary incontinence	0.65 (0.59-0.73)	0.65 (0.57-0.73)	0.63 (0.56-0.69)
Hypercholesterolemia	0.64 (0.59-0.68)	0.65 (0.60-0.69)	0.63 (0.57-0.67)
Articular cartilage disorder	0.64 (0.57-0.69)	0.65 (0.58-0.71)	0.61 (0.54-0.68)
Aortic valve disease	0.65 (0.59-0.70)	0.64 (0.59-0.70)	0.56 (0.50-0.61)
Enthesopathy	0.66 (0.58-0.72)	0.64 (0.57-0.71)	0.63 (0.55-0.71)
Heart failure with preserved EF [Diastolic hear...	0.66 (0.58-0.74)	0.64 (0.56-0.73)	0.55 (0.47-0.63)
Hemorrhage of gastrointestinal tract	0.66 (0.57-0.75)	0.64 (0.57-0.72)	0.50 (0.40-0.58)
Bacterial infection NOS	0.62 (0.56-0.68)	0.64 (0.59-0.70)	0.60 (0.54-0.65)
Osteopenia or other disorder of bone and cartilage	0.64 (0.59-0.69)	0.64 (0.59-0.69)	0.63 (0.58-0.68)
Disorders of protein plasma/amino-acid transpor...	0.65 (0.57-0.74)	0.64 (0.57-0.72)	0.62 (0.55-0.69)
Cardiac shunt/ heart septal defect	0.62 (0.54-0.71)	0.64 (0.55-0.72)	0.54 (0.44-0.63)
Hyposmolality and/or hyponatremia	0.61 (0.56-0.66)	0.64 (0.59-0.69)	0.60 (0.56-0.65)
Heart valve disorders	0.64 (0.59-0.68)	0.64 (0.60-0.68)	0.62 (0.57-0.67)
Disorders of mineral metabolism	0.63 (0.58-0.68)	0.64 (0.59-0.69)	0.62 (0.56-0.68)
Pain in joint	0.63 (0.58-0.68)	0.64 (0.59-0.68)	0.61 (0.57-0.65)
Pulmonary collapse; interstitial and compensato...	0.60 (0.54-0.66)	0.64 (0.57-0.71)	0.55 (0.49-0.62)

Continued on next page

Supplementary Table 4. Mean (95% confidence interval) area under the ROC curve for predicting patient diagnoses, grouped into Phecode phenotypes, using the baseline, methylation data, and genotype data. Confidence intervals determined using bootstrapping.

Phecode Phenotype	Baseline	Methylation	Genotypes
Other forms of chronic heart disease	0.49 (0.40-0.57)	0.64 (0.56-0.71)	0.49 (0.41-0.58)
Other venous embolism and thrombosis	0.57 (0.51-0.64)	0.64 (0.58-0.71)	0.55 (0.48-0.61)
Complications of transplants and reattached limbs	0.63 (0.55-0.72)	0.63 (0.54-0.72)	0.61 (0.51-0.69)
Edema	0.59 (0.53-0.64)	0.63 (0.58-0.68)	0.58 (0.53-0.63)
Benign neoplasm of colon	0.59 (0.53-0.64)	0.63 (0.58-0.69)	0.58 (0.53-0.65)
Symptoms concerning nutrition, metabolism, and ...	0.61 (0.56-0.67)	0.63 (0.57-0.69)	0.59 (0.53-0.66)
Other disorders of bladder	0.62 (0.55-0.70)	0.63 (0.55-0.70)	0.59 (0.51-0.66)
Chronic pulmonary heart disease	0.57 (0.50-0.63)	0.63 (0.57-0.70)	0.55 (0.47-0.61)
Diseases of the larynx and vocal cords	0.49 (0.40-0.58)	0.63 (0.56-0.70)	0.40 (0.32-0.48)
Chronic airway obstruction	0.64 (0.58-0.70)	0.63 (0.56-0.69)	0.61 (0.55-0.69)
Candidiasis	0.63 (0.56-0.70)	0.63 (0.56-0.69)	0.61 (0.54-0.68)
Neoplasm of uncertain behavior	0.58 (0.49-0.66)	0.63 (0.55-0.70)	0.41 (0.33-0.50)
Shortness of breath	0.62 (0.57-0.67)	0.63 (0.58-0.67)	0.61 (0.56-0.65)
Degeneration of intervertebral disc	0.63 (0.57-0.70)	0.62 (0.56-0.69)	0.61 (0.54-0.67)
Symptoms involving respiratory system and other...	0.54 (0.45-0.64)	0.62 (0.54-0.70)	0.62 (0.52-0.70)
Peripheral vascular disease	0.65 (0.59-0.70)	0.62 (0.57-0.67)	0.61 (0.55-0.66)
Heart failure NOS	0.55 (0.50-0.61)	0.62 (0.56-0.68)	0.49 (0.44-0.55)
Cardiomegaly	0.52 (0.48-0.57)	0.62 (0.58-0.67)	0.49 (0.45-0.54)
Congenital anomalies of great vessels	0.64 (0.59-0.70)	0.62 (0.57-0.68)	0.63 (0.58-0.70)
Pleurisy; pleural effusion	0.60 (0.56-0.65)	0.62 (0.58-0.67)	0.58 (0.54-0.63)
Mixed hyperlipidemia	0.63 (0.57-0.68)	0.62 (0.56-0.68)	0.61 (0.55-0.66)
Other aneurysm	0.63 (0.55-0.71)	0.62 (0.54-0.69)	0.60 (0.53-0.67)
Abnormality of gait	0.69 (0.61-0.75)	0.62 (0.56-0.69)	0.66 (0.59-0.73)
Atrial fibrillation and flutter	0.64 (0.59-0.69)	0.62 (0.57-0.66)	0.63 (0.59-0.68)
Infection/inflammation of internal prosthetic d...	0.59 (0.51-0.67)	0.62 (0.54-0.71)	0.60 (0.51-0.68)

Continued on next page

Supplementary Table 4. Mean (95% confidence interval) area under the ROC curve for predicting patient diagnoses, grouped into Phecode phenotypes, using the baseline, methylation data, and genotype data. Confidence intervals determined using bootstrapping.

Phecode Phenotype	Baseline	Methylation	Genotypes
Occlusion and stenosis of precerebral arteries	0.69 (0.61-0.76)	0.62 (0.54-0.70)	0.58 (0.51-0.66)
Other chronic nonalcoholic liver disease	0.60 (0.55-0.67)	0.62 (0.56-0.68)	0.61 (0.54-0.68)
Bacteremia	0.61 (0.54-0.70)	0.61 (0.53-0.70)	0.58 (0.48-0.67)
Bacterial pneumonia	0.64 (0.55-0.72)	0.61 (0.52-0.70)	0.66 (0.58-0.74)
Atrial fibrillation	0.63 (0.58-0.68)	0.61 (0.56-0.66)	0.59 (0.53-0.65)
Substance addiction and disorders	0.62 (0.52-0.70)	0.61 (0.55-0.68)	0.57 (0.49-0.67)
Contusion	0.50 (0.41-0.59)	0.61 (0.53-0.69)	0.49 (0.40-0.58)
Inflammation of the eye	0.65 (0.58-0.71)	0.61 (0.51-0.69)	0.59 (0.52-0.67)
Congestive heart failure (CHF) NOS	0.59 (0.54-0.65)	0.61 (0.55-0.67)	0.53 (0.47-0.58)
Other tests	0.54 (0.46-0.62)	0.61 (0.53-0.68)	0.59 (0.50-0.65)
Other diseases of blood and blood-forming organs	0.58 (0.50-0.66)	0.61 (0.53-0.70)	0.47 (0.38-0.56)
Blood in stool	0.54 (0.46-0.64)	0.61 (0.51-0.68)	0.39 (0.32-0.47)
Iron deficiency anemias	0.63 (0.56-0.70)	0.61 (0.54-0.67)	0.60 (0.53-0.67)
Other hypertensive complications	0.62 (0.55-0.67)	0.60 (0.55-0.66)	0.62 (0.55-0.69)
Hypertensive heart disease	0.61 (0.54-0.67)	0.60 (0.53-0.67)	0.68 (0.61-0.74)
Hypovolemia	0.56 (0.48-0.66)	0.60 (0.51-0.69)	0.47 (0.37-0.55)
Insulin pump user	0.59 (0.53-0.65)	0.60 (0.53-0.66)	0.58 (0.51-0.65)
Primary/intrinsic cardiomyopathies	0.55 (0.50-0.61)	0.60 (0.53-0.68)	0.46 (0.40-0.51)
Peripheral enthesopathies and allied syndromes	0.61 (0.55-0.68)	0.60 (0.54-0.66)	0.57 (0.51-0.63)
Other diseases of lung	0.55 (0.50-0.60)	0.60 (0.54-0.65)	0.52 (0.47-0.57)
Dermatophytosis / Dermatomycosis	0.60 (0.54-0.67)	0.60 (0.53-0.68)	0.60 (0.53-0.65)
Shock	0.59 (0.50-0.66)	0.60 (0.52-0.69)	0.51 (0.42-0.59)
Heart valve replaced	0.56 (0.49-0.63)	0.60 (0.53-0.67)	0.51 (0.43-0.57)
Pain	0.56 (0.52-0.61)	0.60 (0.56-0.64)	0.55 (0.50-0.59)
Dizziness and giddiness (Light-headedness and v...	0.61 (0.54-0.67)	0.60 (0.53-0.65)	0.59 (0.53-0.64)

Continued on next page

Supplementary Table 4. Mean (95% confidence interval) area under the ROC curve for predicting patient diagnoses, grouped into Phecode phenotypes, using the baseline, methylation data, and genotype data. Confidence intervals determined using bootstrapping.

Phecode Phenotype	Baseline	Methylation	Genotypes
Nonrheumatic mitral valve disorders	0.58 (0.53-0.64)	0.60 (0.54-0.65)	0.56 (0.51-0.62)
Diseases of white blood cells	0.52 (0.42-0.59)	0.60 (0.53-0.66)	0.41 (0.35-0.48)
Peripheral vascular disease, unspecified	0.60 (0.54-0.65)	0.59 (0.53-0.65)	0.59 (0.53-0.65)
Cystitis	0.61 (0.53-0.69)	0.59 (0.50-0.67)	0.55 (0.48-0.62)
Diseases of esophagus	0.58 (0.53-0.62)	0.59 (0.55-0.63)	0.54 (0.49-0.58)
Other disorders of synovium, tendon, and bursa	0.61 (0.54-0.69)	0.59 (0.52-0.66)	0.61 (0.54-0.69)
GERD	0.57 (0.53-0.62)	0.59 (0.55-0.64)	0.55 (0.50-0.60)
Other biliary tract disease	0.63 (0.54-0.71)	0.59 (0.51-0.67)	0.59 (0.50-0.68)
Inflammatory and toxic neuropathy	0.53 (0.45-0.61)	0.59 (0.52-0.64)	0.43 (0.36-0.52)
Other disorders of liver	0.60 (0.53-0.65)	0.59 (0.53-0.64)	0.56 (0.49-0.63)
Other infectious and parasitic diseases	0.50 (0.40-0.59)	0.59 (0.49-0.66)	0.57 (0.46-0.66)
Open wounds of head; neck; and trunk	0.44 (0.36-0.54)	0.58 (0.48-0.69)	0.47 (0.38-0.57)
Cerebrovascular disease	0.57 (0.52-0.63)	0.58 (0.52-0.64)	0.53 (0.47-0.60)
Abnormal electrocardiogram [ECG] [EKG]	0.49 (0.44-0.54)	0.58 (0.54-0.64)	0.46 (0.41-0.50)
Generalized anxiety disorder	0.53 (0.46-0.60)	0.58 (0.49-0.67)	0.51 (0.43-0.59)
Lymphadenitis	0.56 (0.48-0.62)	0.58 (0.51-0.65)	0.53 (0.46-0.60)
Secondary diabetes mellitus	0.63 (0.55-0.72)	0.58 (0.50-0.66)	0.67 (0.58-0.76)
Myalgia and myositis unspecified	0.52 (0.46-0.58)	0.58 (0.52-0.64)	0.45 (0.39-0.50)
Hypopotassemia	0.55 (0.46-0.63)	0.58 (0.50-0.67)	0.51 (0.43-0.58)
Other symptoms/disorders or the urinary system	0.57 (0.53-0.62)	0.58 (0.53-0.62)	0.56 (0.51-0.61)
Benign neoplasm of skin	0.59 (0.54-0.64)	0.58 (0.52-0.63)	0.57 (0.52-0.63)
Other disorders of circulatory system	0.44 (0.39-0.50)	0.58 (0.52-0.65)	0.61 (0.54-0.67)
Dysuria	0.52 (0.45-0.59)	0.58 (0.51-0.66)	0.45 (0.39-0.53)
Disorder of skin and subcutaneous tissue NOS	0.58 (0.52-0.64)	0.58 (0.52-0.63)	0.53 (0.47-0.61)
Pulmonary heart disease	0.53 (0.47-0.58)	0.58 (0.52-0.64)	0.56 (0.50-0.63)

Continued on next page

Supplementary Table 4. Mean (95% confidence interval) area under the ROC curve for predicting patient diagnoses, grouped into Phecode phenotypes, using the baseline, methylation data, and genotype data. Confidence intervals determined using bootstrapping.

Phecode Phenotype	Baseline	Methylation	Genotypes
Secondary malignancy of lymph nodes	0.61 (0.52-0.68)	0.58 (0.48-0.69)	0.50 (0.41-0.60)
Gastrointestinal hemorrhage	0.60 (0.52-0.67)	0.58 (0.51-0.65)	0.56 (0.49-0.62)
Other abnormal blood chemistry	0.53 (0.49-0.58)	0.58 (0.53-0.63)	0.49 (0.44-0.54)
Cardiac conduction disorders	0.54 (0.50-0.60)	0.58 (0.53-0.62)	0.52 (0.47-0.57)
Nevus, non-neoplastic	0.58 (0.49-0.66)	0.58 (0.48-0.66)	0.47 (0.39-0.54)
Congestive heart failure; nonhypertensive	0.57 (0.52-0.62)	0.58 (0.52-0.64)	0.53 (0.48-0.58)
Malaise and fatigue	0.59 (0.54-0.64)	0.57 (0.53-0.62)	0.56 (0.51-0.61)
Abnormal findings examination of lungs	0.54 (0.49-0.60)	0.57 (0.52-0.62)	0.50 (0.45-0.55)
Chronic pain	0.58 (0.54-0.63)	0.57 (0.53-0.62)	0.57 (0.52-0.62)
Asthma	0.57 (0.50-0.65)	0.57 (0.50-0.64)	0.50 (0.42-0.57)
Hypothyroidism	0.58 (0.51-0.63)	0.57 (0.51-0.62)	0.54 (0.48-0.60)
Hearing loss	0.60 (0.55-0.66)	0.57 (0.51-0.62)	0.60 (0.53-0.66)
Depression	0.55 (0.49-0.61)	0.57 (0.50-0.63)	0.51 (0.46-0.56)
Other peripheral nerve disorders	0.55 (0.49-0.62)	0.57 (0.51-0.64)	0.50 (0.42-0.58)
Frequency of urination and polyuria	0.58 (0.52-0.64)	0.57 (0.49-0.65)	0.53 (0.46-0.62)
Pericarditis	0.53 (0.44-0.62)	0.57 (0.49-0.65)	0.51 (0.44-0.59)
Retention of urine	0.58 (0.52-0.64)	0.57 (0.49-0.64)	0.52 (0.44-0.59)
Spinal stenosis	0.60 (0.53-0.67)	0.57 (0.50-0.63)	0.56 (0.48-0.64)
Other headache syndromes	0.45 (0.38-0.51)	0.57 (0.51-0.63)	0.50 (0.45-0.56)
Other alveolar and parietoalveolar pneumonopathy	0.62 (0.51-0.71)	0.56 (0.47-0.66)	0.56 (0.46-0.66)
Spinal stenosis of lumbar region	0.61 (0.54-0.68)	0.56 (0.47-0.65)	0.54 (0.47-0.63)
Proteinuria	0.53 (0.45-0.60)	0.56 (0.49-0.63)	0.50 (0.42-0.59)
Cardiomyopathy	0.52 (0.46-0.59)	0.56 (0.50-0.63)	0.46 (0.40-0.53)
Mitral valve disease	0.56 (0.50-0.63)	0.56 (0.50-0.62)	0.58 (0.52-0.64)
Visual disturbances	0.53 (0.44-0.59)	0.56 (0.49-0.65)	0.58 (0.50-0.65)

Continued on next page

Supplementary Table 4. Mean (95% confidence interval) area under the ROC curve for predicting patient diagnoses, grouped into Phecode phenotypes, using the baseline, methylation data, and genotype data. Confidence intervals determined using bootstrapping.

Phecode Phenotype	Baseline	Methylation	Genotypes
Insomnia	0.52 (0.46-0.59)	0.56 (0.49-0.61)	0.53 (0.46-0.58)
Cough	0.57 (0.53-0.62)	0.56 (0.52-0.61)	0.56 (0.53-0.61)
Diseases of hair and hair follicles	0.44 (0.35-0.53)	0.56 (0.48-0.65)	0.49 (0.38-0.58)
Other symptoms of respiratory system	0.58 (0.54-0.62)	0.56 (0.52-0.61)	0.56 (0.52-0.60)
Back pain	0.58 (0.53-0.63)	0.56 (0.51-0.60)	0.55 (0.51-0.60)
Neurological disorders	0.55 (0.47-0.61)	0.56 (0.48-0.62)	0.54 (0.47-0.62)
Dermatophytosis	0.59 (0.52-0.67)	0.56 (0.49-0.62)	0.56 (0.48-0.63)
Alcohol-related disorders	0.65 (0.54-0.76)	0.56 (0.45-0.64)	0.58 (0.48-0.68)
Pulmonary congestion and hypostasis	0.63 (0.56-0.70)	0.56 (0.48-0.65)	0.59 (0.50-0.67)
Mood disorders	0.55 (0.49-0.60)	0.56 (0.50-0.61)	0.49 (0.44-0.55)
Cancer, suspected or other	0.57 (0.51-0.64)	0.56 (0.50-0.63)	0.60 (0.53-0.66)
Hypotension NOS	0.57 (0.50-0.62)	0.56 (0.50-0.61)	0.52 (0.46-0.59)
Other dyspnea	0.57 (0.52-0.61)	0.56 (0.50-0.62)	0.53 (0.48-0.58)
Heart failure with reduced EF [Systolic or comb...	0.59 (0.53-0.66)	0.56 (0.48-0.63)	0.56 (0.50-0.63)
Migraine	0.58 (0.49-0.67)	0.56 (0.47-0.66)	0.55 (0.45-0.66)
Abnormal findings on exam of gastrointestinal t...	0.55 (0.49-0.65)	0.56 (0.47-0.64)	0.49 (0.41-0.60)
Sleep disorders	0.57 (0.51-0.63)	0.56 (0.51-0.61)	0.52 (0.46-0.57)
Other local infections of skin and subcutaneous...	0.57 (0.48-0.66)	0.56 (0.47-0.64)	0.53 (0.43-0.63)
Anxiety disorder	0.53 (0.47-0.59)	0.56 (0.51-0.61)	0.46 (0.41-0.52)
Hemangioma and lymphangioma, any site	0.56 (0.47-0.65)	0.56 (0.47-0.66)	0.45 (0.36-0.55)
Hypothyroidism NOS	0.60 (0.55-0.66)	0.55 (0.50-0.60)	0.58 (0.52-0.63)
Spondylosis and allied disorders	0.56 (0.50-0.63)	0.55 (0.49-0.62)	0.53 (0.46-0.59)
Abdominal hernia	0.50 (0.45-0.55)	0.55 (0.49-0.61)	0.46 (0.40-0.51)
Cardiac pacemaker in situ	0.42 (0.34-0.52)	0.55 (0.46-0.64)	0.55 (0.47-0.64)
Cerebral ischemia	0.45 (0.36-0.54)	0.55 (0.46-0.63)	0.46 (0.38-0.53)

Continued on next page

Supplementary Table 4. Mean (95% confidence interval) area under the ROC curve for predicting patient diagnoses, grouped into Phecode phenotypes, using the baseline, methylation data, and genotype data. Confidence intervals determined using bootstrapping.

Phecode Phenotype	Baseline	Methylation	Genotypes
Malignant neoplasm, other	0.52 (0.44-0.59)	0.55 (0.48-0.62)	0.46 (0.40-0.53)
Dermatophytosis of nail	0.59 (0.52-0.68)	0.55 (0.46-0.65)	0.46 (0.37-0.55)
Cardiac dysrhythmias	0.56 (0.51-0.60)	0.55 (0.50-0.59)	0.55 (0.50-0.59)
Cyst of kidney, acquired	0.53 (0.45-0.60)	0.55 (0.47-0.62)	0.46 (0.38-0.53)
Impacted cerumen	0.55 (0.46-0.63)	0.55 (0.46-0.62)	0.44 (0.36-0.51)
Abnormal heart sounds	0.56 (0.51-0.62)	0.55 (0.48-0.62)	0.49 (0.43-0.56)
Neoplasm of uncertain behavior of skin	0.58 (0.52-0.65)	0.55 (0.47-0.62)	0.57 (0.50-0.63)
Palpitations	0.49 (0.43-0.55)	0.54 (0.47-0.62)	0.51 (0.45-0.58)
Dysphagia	0.53 (0.46-0.59)	0.54 (0.46-0.61)	0.55 (0.48-0.62)
Abnormal glucose	0.55 (0.50-0.61)	0.54 (0.48-0.59)	0.50 (0.45-0.55)
Functional digestive disorders	0.49 (0.40-0.57)	0.54 (0.45-0.63)	0.68 (0.58-0.78)
Syncope and collapse	0.43 (0.36-0.50)	0.54 (0.46-0.62)	0.51 (0.42-0.60)
Pruritus and related conditions	0.47 (0.37-0.55)	0.54 (0.44-0.63)	0.69 (0.61-0.75)
Allergic rhinitis	0.56 (0.49-0.63)	0.54 (0.46-0.61)	0.57 (0.52-0.64)
Esophagitis, GERD and related diseases	0.56 (0.51-0.60)	0.54 (0.49-0.59)	0.53 (0.49-0.58)
Cardiac congenital anomalies	0.46 (0.39-0.53)	0.54 (0.46-0.61)	0.53 (0.44-0.61)
Major depressive disorder	0.55 (0.48-0.63)	0.54 (0.45-0.61)	0.50 (0.42-0.57)
Hemorrhoids	0.63 (0.56-0.71)	0.54 (0.46-0.62)	0.55 (0.46-0.64)
Premature beats	0.55 (0.48-0.63)	0.53 (0.47-0.59)	0.56 (0.48-0.63)
Ill-defined descriptions and complications of h...	0.57 (0.50-0.62)	0.53 (0.46-0.60)	0.49 (0.42-0.57)
Abnormal results of function study of liver	0.60 (0.51-0.69)	0.53 (0.44-0.62)	0.54 (0.45-0.62)
Chronic sinusitis	0.42 (0.36-0.49)	0.53 (0.46-0.61)	0.46 (0.39-0.54)
Hepatomegaly	0.51 (0.44-0.59)	0.53 (0.46-0.60)	0.51 (0.42-0.58)
Diaphragmatic hernia	0.56 (0.49-0.63)	0.53 (0.46-0.60)	0.53 (0.46-0.60)
Thoracic or lumbosacral neuritis or radiculitis...	0.51 (0.44-0.59)	0.53 (0.47-0.61)	0.48 (0.42-0.55)

Continued on next page

Supplementary Table 4. Mean (95% confidence interval) area under the ROC curve for predicting patient diagnoses, grouped into Phecode phenotypes, using the baseline, methylation data, and genotype data. Confidence intervals determined using bootstrapping.

Phecode Phenotype	Baseline	Methylation	Genotypes
Nonspecific chest pain	0.51 (0.46-0.55)	0.53 (0.48-0.57)	0.44 (0.39-0.49)
Spondylosis without myelopathy	0.55 (0.48-0.62)	0.53 (0.45-0.60)	0.48 (0.41-0.55)
Atopic/contact dermatitis due to other or unspe...	0.55 (0.50-0.62)	0.53 (0.47-0.60)	0.56 (0.50-0.63)
Noninfectious gastroenteritis	0.47 (0.39-0.56)	0.53 (0.45-0.60)	0.53 (0.45-0.60)
Voice disturbance	0.53 (0.46-0.62)	0.53 (0.43-0.61)	0.35 (0.26-0.43)
Chronic kidney disease, Stage I or II	0.49 (0.43-0.56)	0.53 (0.45-0.61)	0.41 (0.35-0.48)
Other specified cardiac dysrhythmias	0.56 (0.51-0.62)	0.53 (0.46-0.58)	0.52 (0.47-0.58)
Nonrheumatic tricuspid valve disorders	0.50 (0.41-0.57)	0.53 (0.45-0.60)	0.45 (0.37-0.53)
Transient cerebral ischemia	0.45 (0.37-0.53)	0.52 (0.44-0.60)	0.62 (0.54-0.68)
Intervertebral disc disorders	0.57 (0.51-0.63)	0.52 (0.46-0.58)	0.55 (0.49-0.60)
Nonspecific findings on examination of blood	0.45 (0.37-0.51)	0.52 (0.45-0.60)	0.53 (0.44-0.59)
Superficial cellulitis and abscess	0.59 (0.52-0.65)	0.52 (0.45-0.59)	0.59 (0.53-0.66)
Other abnormal glucose	0.51 (0.46-0.57)	0.52 (0.48-0.57)	0.43 (0.38-0.48)
Vitamin D deficiency	0.56 (0.51-0.62)	0.52 (0.47-0.58)	0.53 (0.47-0.59)
Rheumatic disease of the heart valves	0.50 (0.42-0.57)	0.52 (0.46-0.59)	0.44 (0.38-0.51)
Cancer of urinary organs (incl. kidney and blad...	0.54 (0.46-0.63)	0.52 (0.42-0.62)	0.45 (0.37-0.53)
Symptoms and disorders of the joints	0.57 (0.50-0.64)	0.52 (0.45-0.60)	0.58 (0.49-0.66)
Empyema and pneumothorax	0.56 (0.48-0.64)	0.52 (0.41-0.60)	0.52 (0.44-0.60)
Varicose veins	0.54 (0.47-0.63)	0.52 (0.43-0.63)	0.39 (0.32-0.47)
Other disorders of biliary tract	0.57 (0.50-0.66)	0.52 (0.42-0.61)	0.49 (0.39-0.59)
Sciatica	0.47 (0.39-0.55)	0.52 (0.44-0.59)	0.49 (0.41-0.58)
Abdominal pain	0.54 (0.50-0.59)	0.52 (0.47-0.57)	0.55 (0.50-0.60)
Swelling of limb	0.57 (0.51-0.63)	0.52 (0.46-0.58)	0.54 (0.49-0.61)
Circulatory disease NEC	0.47 (0.40-0.54)	0.51 (0.45-0.57)	0.50 (0.44-0.57)
Sprains and strains	0.60 (0.53-0.67)	0.51 (0.43-0.59)	0.52 (0.45-0.60)

Continued on next page

Supplementary Table 4. Mean (95% confidence interval) area under the ROC curve for predicting patient diagnoses, grouped into Phecode phenotypes, using the baseline, methylation data, and genotype data. Confidence intervals determined using bootstrapping.

Phecode Phenotype	Baseline	Methylation	Genotypes
Gout	0.55 (0.46-0.63)	0.51 (0.43-0.60)	0.56 (0.48-0.65)
Hematuria	0.56 (0.49-0.63)	0.51 (0.43-0.57)	0.50 (0.43-0.57)
Arrhythmia (cardiac) NOS	0.53 (0.46-0.59)	0.50 (0.43-0.58)	0.54 (0.47-0.62)
Dyschromia and Vitiligo	0.58 (0.51-0.65)	0.50 (0.41-0.58)	0.49 (0.43-0.57)
Elevated white blood cell count	0.52 (0.44-0.59)	0.50 (0.43-0.58)	0.37 (0.30-0.43)
Encounter for long-term (current) use of antico...	0.53 (0.46-0.61)	0.50 (0.45-0.58)	0.50 (0.41-0.57)
Anxiety disorders	0.54 (0.50-0.60)	0.50 (0.44-0.55)	0.50 (0.44-0.55)
Allergy/adverse effect of penicillin	0.53 (0.45-0.61)	0.50 (0.42-0.60)	0.55 (0.45-0.64)
Diseases of sebaceous glands	0.49 (0.41-0.59)	0.50 (0.42-0.58)	0.61 (0.54-0.68)
Rash and other nonspecific skin eruption	0.54 (0.48-0.61)	0.50 (0.43-0.56)	0.45 (0.39-0.52)
Acute upper respiratory infections of multiple ...	0.52 (0.45-0.57)	0.49 (0.44-0.55)	0.53 (0.48-0.59)
Hypercalcemia	0.58 (0.48-0.67)	0.49 (0.41-0.57)	0.46 (0.38-0.55)
Other upper respiratory disease	0.49 (0.43-0.56)	0.49 (0.42-0.57)	0.51 (0.43-0.58)
Abnormal serum enzyme levels	0.48 (0.42-0.55)	0.49 (0.41-0.56)	0.47 (0.39-0.55)
Paroxysmal ventricular tachycardia	0.49 (0.43-0.58)	0.49 (0.41-0.57)	0.43 (0.36-0.52)
Sinoatrial node dysfunction (Bradycardia)	0.60 (0.50-0.69)	0.49 (0.40-0.56)	0.64 (0.55-0.72)
Vitamin deficiency	0.54 (0.49-0.59)	0.49 (0.43-0.54)	0.51 (0.46-0.56)
Other disorders of arteries and arterioles	0.46 (0.39-0.53)	0.49 (0.42-0.57)	0.44 (0.38-0.52)
Gout and other crystal arthropathies	0.53 (0.43-0.61)	0.49 (0.41-0.56)	0.57 (0.50-0.66)
Chronic ulcer of skin	0.49 (0.42-0.58)	0.49 (0.40-0.57)	0.41 (0.33-0.48)
Swelling, mass, or lump in head and neck [Space...	0.46 (0.38-0.54)	0.49 (0.40-0.58)	0.59 (0.50-0.66)
Cardiac and circulatory congenital anomalies	0.50 (0.42-0.56)	0.48 (0.42-0.54)	0.40 (0.32-0.47)
Nonspecific abnormal findings on radiological a...	0.54 (0.45-0.63)	0.48 (0.40-0.57)	0.50 (0.41-0.58)
Erythematous conditions	0.57 (0.49-0.67)	0.48 (0.39-0.58)	0.51 (0.41-0.60)
Poisoning by antibiotics	0.51 (0.43-0.59)	0.48 (0.39-0.57)	0.59 (0.50-0.68)

Continued on next page

Supplementary Table 4. Mean (95% confidence interval) area under the ROC curve for predicting patient diagnoses, grouped into Phecode phenotypes, using the baseline, methylation data, and genotype data. Confidence intervals determined using bootstrapping.

Phecode Phenotype	Baseline	Methylation	Genotypes
Solitary pulmonary nodule	0.53 (0.45-0.60)	0.48 (0.42-0.54)	0.43 (0.34-0.50)
Disorders of refraction and accommodation; blin...	0.59 (0.51-0.70)	0.48 (0.39-0.59)	0.49 (0.39-0.60)
Disturbance of skin sensation	0.50 (0.43-0.58)	0.47 (0.40-0.56)	0.49 (0.41-0.58)
Paroxysmal tachycardia, unspecified	0.41 (0.34-0.49)	0.47 (0.38-0.55)	0.40 (0.32-0.49)
Disease of tricuspid valve	0.45 (0.38-0.54)	0.47 (0.38-0.55)	0.55 (0.48-0.64)
Other symptoms involving abdomen and pelvis	0.56 (0.47-0.62)	0.47 (0.39-0.55)	0.55 (0.47-0.61)
Other specified diseases of hair and hair folli...	0.39 (0.31-0.47)	0.47 (0.38-0.56)	0.52 (0.43-0.61)
Other dyschromia	0.56 (0.48-0.63)	0.46 (0.38-0.54)	0.50 (0.42-0.58)
Valvular heart disease/ heart chambers	0.48 (0.41-0.56)	0.45 (0.37-0.54)	0.54 (0.45-0.62)
Heart transplant/surgery	0.49 (0.40-0.58)	0.45 (0.37-0.53)	0.48 (0.41-0.55)
Calculus of kidney	0.54 (0.47-0.61)	0.45 (0.37-0.52)	0.52 (0.45-0.58)
Acute pharyngitis	0.43 (0.35-0.51)	0.45 (0.37-0.53)	0.42 (0.35-0.48)
Acute bronchitis and bronchiolitis	0.52 (0.44-0.61)	0.44 (0.36-0.55)	0.55 (0.45-0.63)
Diseases of pancreas	0.51 (0.42-0.58)	0.44 (0.37-0.53)	0.43 (0.36-0.49)
Paroxysmal supraventricular tachycardia	0.49 (0.42-0.57)	0.43 (0.34-0.51)	0.57 (0.48-0.65)
Opiates and related narcotics causing adverse e...	0.59 (0.50-0.68)	0.43 (0.35-0.51)	0.45 (0.36-0.54)
Other diseases of respiratory system, not elsew...	0.51 (0.40-0.60)	0.42 (0.34-0.50)	0.49 (0.40-0.57)
Cervicalgia	0.51 (0.44-0.59)	0.42 (0.36-0.51)	0.52 (0.44-0.59)
Complications of cardiac/vascular device, impla...	0.54 (0.47-0.61)	0.42 (0.32-0.53)	0.51 (0.42-0.59)
Other hypertrophic and atrophic conditions of skin	0.55 (0.47-0.63)	0.40 (0.32-0.47)	0.42 (0.36-0.50)

Supplementary Table 5. The imputation accuracy, p-value and number of CpGs selected for significantly imputed MRS that also significantly improved over the baseline model.

Pharmaceutical Subclass	Medication GPI	AUC	p-value	Number of CpGs
Phosphate Binder Agents	5280	0.876	1.106E-50	2715
Hematopoietic Growth Factors	8240	0.840	1.747E-45	996
Immunosuppressive Agents	9940	0.828	9.443E-41	2870
CMV Agents	1220	0.905	1.720E-38	1223
Osmotic Diuretics	3740	0.848	6.372E-34	510
B-Complex w/ Folic Acid	7813	0.836	3.442E-31	2593
Metabolic Modifiers	3090	0.813	1.213E-28	2200
Prostatic Hypertrophy Agents	5685	0.779	2.411E-25	2586
Plasma Proteins	8540	0.739	4.379E-24	3353
Proton Pump Inhibitors	4927	0.693	3.500E-23	1097
Anti-infectives - Throat	8810	0.765	2.443E-20	453
Imidazole-Related Antifungals	1140	0.720	2.249E-17	2507
Vasodilators	3640	0.708	6.753E-17	654
Glucocorticosteroids	2210	0.663	1.354E-16	4508
Loop Diuretics	3720	0.674	5.020E-16	4529
Anti-infective Misc. - Combinations	1699	0.665	7.711E-16	3503
Analgesics Other	6420	0.651	1.084E-15	833
Antihistamines - Ethanolamines	4120	0.660	1.056E-14	649
Laxatives - Miscellaneous	4660	0.648	4.984E-14	4203
Anti-infective Agents - Misc.	1600	0.674	9.525E-14	657
5-HT3 Receptor Antagonists	5025	0.653	1.049E-13	4570
Fluoroquinolones	500	0.658	1.885E-13	510
Benzodiazepines	5710	0.658	1.957E-13	4482
Potassium Removing Agents	9945	0.736	2.568E-13	2048
Cephalosporins - 3rd Generation	230	0.665	4.926E-13	2379

Continued on next page

Supplementary Table 5. The imputation accuracy, p-value and number of CpGs selected for significantly imputed MRS that also significantly improved over the baseline model.

Pharmaceutical Subclass	Medication GPI	AUC	p-value	Number of CpGs
Stimulant Laxatives	4620	0.642	1.014E-12	3949
Calcium Channel Blockers	3400	0.638	3.149E-12	4658
Insulin	2710	0.654	3.947E-12	3385
Carbohydrates	8010	0.652	5.207E-12	4246
Thrombolytic Enzymes	8560	0.747	5.481E-12	1504
Parenteral Therapy Supplies	9705	0.709	1.580E-11	554
Local Anesthetic Combinations	6999	0.655	2.178E-11	3235
Heparins And Heparinoid-Like Agents	8310	0.637	2.486E-11	607
Electrolyte Mixtures	7999	0.634	4.224E-11	591
Potassium	7970	0.643	4.564E-11	870
Calcium	7910	0.659	6.164E-11	3126
Alpha-Beta Blockers	3330	0.647	7.206E-11	4029
Beta Blockers Cardio-Selective	3320	0.618	8.699E-11	5159
Glycopeptides	1628	0.639	2.870E-10	2999
Saline Laxatives	4610	0.643	3.819E-10	3509
Magnesium	7940	0.636	1.011E-09	3643
Diagnostic Radiopharmaceuticals	9435	0.630	4.355E-09	652
Antiseptics - Mouth/Throat	8815	0.673	5.057E-09	2134
Vasopressors	3800	0.624	9.485E-09	4378
Phenothiazines	5920	0.616	1.141E-07	353
Antiperistaltic Agents	4710	0.655	3.923E-07	468
Alternative Medicine - C's	9509	0.658	2.111E-06	135
Liquid Vehicles	9840	0.655	2.814E-06	1372
Antiflatulents	5220	0.624	5.187E-06	1750
Alternative Medicine - M's	9539	0.652	6.305E-06	1792

Continued on next page

Supplementary Table 5. The imputation accuracy, p-value and number of CpGs selected for significantly imputed MRS that also significantly improved over the baseline model.

**Pharmaceutical Subclass**	**Medication GPI**	**AUC**	**p-value**	**Number of CpGs**
Misc. Nutritional Substances	8050	0.616	1.029E-05	2702
Gallstone Solubilizing Agents	5210	0.724	1.071E-05	1177
Thiazides and Thiazide-Like Diuretics	3760	0.629	1.291E-05	73
Corticosteroids - Topical	9055	0.575	1.354E-04	44

**Lab**	R^2	**p-value**	**Number of CpGs**
Creatinine	0.457	1.266E-95	32364
Urea Nitrogen	0.435	2.502E-87	4218
Absolute Eos Count	0.352	3.960E-51	3602
Hemoglobin	0.284	3.024E-50	25995
Hematocrit	0.246	1.144E-42	1827
Neutrophil Percent, Auto	0.264	1.139E-36	897
Mean Corpuscular Hemoglobin	0.208	7.037E-35	33151
Mean Corpuscular Volume	0.183	1.439E-30	60383
Platelet Count, Auto	0.168	1.956E-28	15362
Chloride	0.141	1.493E-24	44943
Albumin	0.143	6.472E-22	6020
Absolute Immature Gran Count	0.155	1.545E-20	1705
White Blood Cell Count	0.120	3.405E-20	29995
Absolute Neut Count	0.149	4.974E-20	19924
Sodium	0.115	5.060E-20	35598
Absolute Lymphocyte Count	0.128	2.920E-17	10575
Absolute Mono Count	0.121	2.579E-16	3675
Neutrophils Abs (Prelim)	0.116	1.263E-15	18192
Glucose	0.075	4.033E-13	973
Absolute Baso Count	0.086	7.020E-12	566
Hgb A1c - Hplc	0.125	2.588E-11	902
Ferritin	0.122	1.331E-07	40620
Iron Binding Capacity	0.104	8.842E-07	7121
Qrs Duration	0.052	1.722E-06	4731
Anion Gap	0.027	1.729E-05	29869
Alanine Aminotransferase	0.028	2.801E-05	546
Cholesterol, Hdl	0.054	4.612E-05	60
Ventricular Rate	0.033	1.499E-04	92303
Bilirubin,Total	0.022	3.094E-04	535

Supplementary Table 6. The imputation accuracy, p-value and number of CpGs selected for significantly imputed MRS that also significantly improved over the baseline model.

Supplementary Table 7. The imputation accuracy, p-value and number of CpGs selected for significantly imputed MRS that also significantly improved over the baseline model.

Phenotype	Phecode	AUC	p-value	Number of CpGs
End stage renal disease	585.32	0.898	5.459E-72	2790
Chronic renal failure [CKD]	585.3	0.820	1.814E-56	4357
Renal dialysis	585.31	0.880	9.402E-56	2654
Renal failure	585.0	0.798	6.810E-51	4836
Hypertensive chronic kidney disease	401.22	0.801	1.446E-42	3851
Immunity deficiency	279.1	0.821	7.942E-33	624
Anemia of chronic disease	285.2	0.789	1.395E-32	3391
Disorders involving the immune mechanism	279.0	0.799	1.072E-30	2681
Anemia in chronic kidney disease	285.21	0.813	5.977E-27	537
Kidney replaced by transpant	587.0	0.813	8.000E-27	605
Morbid obesity	278.11	0.847	2.416E-26	158
Cirrhosis of liver without mention of alcohol	571.51	0.856	2.588E-25	441
Essential hypertension	401.1	0.701	1.007E-22	4666
Disorders resulting from impaired renal function	588.0	0.796	1.982E-20	1944
Decreased white blood cell count	288.1	0.811	7.196E-20	1716
Neutropenia	288.11	0.836	1.106E-19	1137
Type 2 diabetes	250.2	0.698	8.981E-19	500
Hypertensive heart and/or renal disease	401.2	0.723	1.408E-17	2804
Acute renal failure	585.1	0.704	1.739E-17	551
Hypertension	401.0	0.674	1.852E-17	563
Secondary hyperparathyroidism (of renal origin)	588.2	0.763	1.011E-15	1942
Poisoning by primarily systemic agents	963.0	0.818	1.029E-14	1022
Other anemias	285.0	0.667	1.134E-14	4294
Other disorders of the kidney and ureters	586.0	0.685	3.876E-14	3263
Hyperpotassemia	276.13	0.706	1.047E-13	2642

Continued on next page

Supplementary Table 7. The imputation accuracy, p-value and number of CpGs selected for significantly imputed MRS that also significantly improved over the baseline model.

Phenotype	Phecode	AUC	p-value	Number of CpGs
Fluid overload	276.6	0.756	3.137E-13	1667
Acid-base balance disorder	276.4	0.733	3.331E-13	1863
Respiratory failure, insufficiency, arrest	509.0	0.729	1.338E-11	491
Portal hypertension	571.81	0.803	2.149E-11	1347
Chronic liver disease and cirrhosis	571.0	0.707	6.362E-11	429
Disorders of fluid, electrolyte, and acid-base balance	276.0	0.664	7.581E-11	3827
Abnormal involuntary movements	350.1	0.757	7.668E-10	284
Liver abscess and sequelae of chronic liver disease	571.8	0.782	9.916E-10	1420
Purpura and other hemorrhagic conditions	287.0	0.714	1.059E-09	1747
Liver replaced by transplant	573.2	0.764	1.119E-09	317
Thrombocytopenia	287.3	0.697	1.367E-09	433
Splenomegaly	579.2	0.705	6.780E-09	350
Coagulation defects	286.0	0.738	1.068E-08	453
Altered mental status	292.4	0.722	1.817E-08	1054
Nephritis and nephropathy without mention of glomerulonephritis	580.3	0.679	2.788E-08	1640
Type 2 diabetes with renal manifestations	250.22	0.680	3.467E-08	1716
Nephritis; nephrosis; renal sclerosis	580.0	0.664	4.350E-08	2296
Nausea and vomiting	789.0	0.650	7.959E-08	2725
Antineoplastic and immunosuppressive drugsadverse effects	963.1	0.786	3.432E-07	290
Acute pain	338.1	0.649	4.095E-07	2306
Cardiomegaly	416.0	0.622	4.106E-07	3672
Senile cataract	366.2	0.657	4.269E-07	1875

Continued on next page

Supplementary Table 7. The imputation accuracy, p-value and number of CpGs selected for significantly imputed MRS that also significantly improved over the baseline model.

Phenotype	Phecode	AUC	p-value	Number of CpGs
Other disorders of metabolism	277.0	0.704	5.053E-07	1362
Chronic Kidney Disease, Stage III	585.33	0.651	1.493E-06	515
Renal failure NOS	585.2	0.692	1.697E-06	1197
Chronic Kidney Disease, Stage IV	585.34	0.675	3.079E-06	1956
Atherosclerosis	440.0	0.660	8.130E-06	329
Heart failure NOS	428.2	0.623	1.243E-05	2759
Poisoning by hormones and synthetic substitutes	962.0	0.690	1.919E-05	1061
Iron deficiency anemia secondary to blood loss (chronic)	280.2	0.667	3.463E-05	269
Other hypertrophic and atrophic conditions of skin	701.0	0.632	5.942E-05	951

Supplementary Table 8. Number of samples with reported usage of medications in the pharmaceutical subclasses. Pharmaceutical subclasses are sorted by number of samples.

Pharmaceutical Subclass	Number of Samples (Percent)
Sodium	699 (80.9%)
Opioid Agonists	639 (74.0%)
Local Anesthetics - Amides	589 (68.2%)
Non-Barbiturate Hypnotics	584 (67.6%)
5-HT3 Receptor Antagonists	549 (63.5%)
Analgesics Other	544 (63.0%)
Radiographic Contrast Media	535 (61.9%)
Anesthetics - Misc.	507 (58.7%)
Glucocorticosteroids	499 (57.8%)
Salicylates	459 (53.1%)
Heparins And Heparinoid-Like Agents	459 (53.1%)
Opioid Combinations	458 (53.0%)
HMG CoA Reductase Inhibitors	456 (52.8%)
Proton Pump Inhibitors	443 (51.3%)
Oil Soluble Vitamins	434 (50.2%)
Vasopressors	421 (48.7%)
Surfactant Laxatives	398 (46.1%)
Electrolyte Mixtures	390 (45.1%)
Antiarrhythmics Type I-B	383 (44.3%)
Beta Blockers Cardio-Selective	383 (44.3%)
Cephalosporins - 1st Generation	369 (42.7%)
Calcium Channel Blockers	367 (42.5%)
Loop Diuretics	346 (40.0%)
Miscellaneous Contrast Media	341 (39.5%)
Nondepolarizing Muscle Relaxants	336 (38.9%)
Fluoroquinolones	327 (37.8%)
Stimulant Laxatives	326 (37.7%)

Nonsteroidal Anti-inflammatory Agents (NSAIDs)	313 (36.2%)
Sympathomimetics	308 (35.6%)
Antihistamines - Ethanolamines	301 (34.8%)
Laxatives - Miscellaneous	293 (33.9%)
Magnesium	290 (33.6%)
Local Anesthetics - Topical	280 (32.4%)
Potassium	277 (32.1%)
Insulin	269 (31.1%)
Benzodiazepines	265 (30.7%)
Diagnostic Radiopharmaceuticals	264 (30.6%)
Anticonvulsants - Misc.	260 (30.1%)
Carbohydrates	252 (29.2%)
Saline Laxatives	250 (28.9%)
Antispasmodics	250 (28.9%)
H-2 Antagonists	232 (26.9%)
Angiotensin II Receptor Antagonists	231 (26.7%)
ACE Inhibitors	225 (26.0%)
Penicillin Combinations	219 (25.3%)
Cephalosporins - 3rd Generation	217 (25.1%)
Nitrates	215 (24.9%)
Glycopeptides	213 (24.7%)
Alpha-Beta Blockers	210 (24.3%)
Calcium	207 (24.0%)
Multivitamins	207 (24.0%)
Local Anesthetic Combinations	200 (23.1%)
Anti-infective Misc. - Combinations	198 (22.9%)
Anti-infective Agents - Misc.	193 (22.3%)
Plasma Proteins	190 (22.0%)
Diagnostic Drugs	189 (21.9%)
Water Soluble Vitamins	188 (21.8%)

Phenothiazines	184 (21.3%)
Gastrointestinal Stimulants	182 (21.1%)
Corticosteroids - Topical	182 (21.1%)
Central Muscle Relaxants	181 (20.9%)
Viral Vaccines	175 (20.3%)
Iron	170 (19.7%)
Vasodilators	168 (19.4%)
Antibiotics - Topical	166 (19.2%)
Hematopoietic Growth Factors	165 (19.1%)
Azithromycin	164 (19.0%)
Antacids - Calcium Salts	164 (19.0%)
Antimyasthenic/Cholinergic Agents	158 (18.3%)
Nasal Steroids	158 (18.3%)
Selective Serotonin Reuptake Inhibitors (SSRIs)	157 (18.2%)
Thiazides and Thiazide-Like Diuretics	157 (18.2%)
Misc. Nutritional Substances	155 (17.9%)
Opioid Antagonists	155 (17.9%)
Platelet Aggregation Inhibitors	154 (17.8%)
Thyroid Hormones	149 (17.2%)
Antifungals - Topical	149 (17.2%)
Bacterial Vaccines	144 (16.7%)
Immunosuppressive Agents	142 (16.4%)
Phosphate Binder Agents	140 (16.2%)
Serotonin Modulators	136 (15.7%)
Laxative Combinations	136 (15.7%)
Biguanides	135 (15.6%)
Depolarizing Muscle Relaxants	135 (15.6%)
Genitourinary Irrigants	134 (15.5%)
Prostatic Hypertrophy Agents	134 (15.5%)
Bronchodilators - Anticholinergics	131 (15.2%)

Antiflatulents	130 (15.0%)
Antacid Combinations	127 (14.7%)
Aminopenicillins	126 (14.6%)
Imidazole-Related Antifungals	125 (14.5%)
Diagnostic Tests	120 (13.9%)
Cobalamins	118 (13.7%)
Folic Acid/Folates	116 (13.4%)
B-Complex w/ Folic Acid	116 (13.4%)
Antihistamines - Non-Sedating	113 (13.1%)
Anesthetics Topical Oral	108 (12.5%)
Diabetic Supplies	107 (12.4%)
Osmotic Diuretics	106 (12.3%)
Tetracyclines	105 (12.2%)
Multiple Vitamins w/ Minerals	105 (12.2%)
Ophthalmic Anti-infectives	104 (12.0%)
Metabolic Modifiers	102 (11.8%)
Potassium Removing Agents	102 (11.8%)
Potassium Sparing Diuretics	101 (11.7%)
Hemostatics - Topical	101 (11.7%)
Ophthalmics - Misc.	101 (11.7%)
Gout Agents	100 (11.6%)
Alternative Medicine - M's	99 (11.5%)
Parenteral Therapy Supplies	99 (11.5%)
Cough/Cold/Allergy Combinations	99 (11.5%)
Antiseptics - Mouth/Throat	98 (11.3%)
Direct Factor Xa Inhibitors	97 (11.2%)
Anti-infectives - Throat	94 (10.9%)
Anti-inflammatory Agents - Topical	93 (10.8%)
Coumarin Anticoagulants	92 (10.6%)
Posterior Pituitary Hormones	91 (10.5%)

Antidotes and Specific Antagonists	90 (10.4%)
Antiadrenergic Antihypertensives	90 (10.4%)
Ophthalmic Steroids	90 (10.4%)
Antitussives	88 (10.2%)
Lincosamides	84 (9.7%)
Dibenzapines	83 (9.6%)
Bone Density Regulators	81 (9.4%)
Antianxiety Agents - Misc.	80 (9.3%)
Phosphate	78 (9.0%)
Antiemetics - Anticholinergic	77 (8.9%)
Antiperistaltic Agents	76 (8.8%)
Herpes Agents	76 (8.8%)
Bicarbonates	75 (8.7%)
Liquid Vehicles	72 (8.3%)
Antiarrhythmics Type III	72 (8.3%)
Artificial Tears and Lubricants	71 (8.2%)
Antidiarrheal/Probiotic Agents - Misc.	71 (8.2%)
Toxoid Combinations	70 (8.1%)
Urinary Antispasmodic - Antimuscarinics (Antich...	67 (7.8%)
Lozenges	67 (7.8%)
CMV Agents	66 (7.6%)
Thrombolytic Enzymes	66 (7.6%)
Impotence Agents	65 (7.5%)
Alternative Medicine - C's	64 (7.4%)
Sulfonylureas	63 (7.3%)
Antihypertensive Combinations	63 (7.3%)
Specialty Vitamins Products	63 (7.3%)
Aminoglycosides	61 (7.1%)
Cephalosporins - 2nd Generation	60 (6.9%)
Alkalinizers	59 (6.8%)

Opioid Partial Agonists	73 (6.8%)
Urinary Anti-infectives	58 (6.7%)
Irrigation Solutions	58 (6.7%)
Influenza Agents	57 (6.6%)
Expectorants	57 (6.6%)
Beta Blockers Non-Selective	56 (6.5%)
Tricyclic Agents	56 (6.5%)
Serotonin-Norepinephrine Reuptake Inhibitors (S...	56 (6.5%)
Cephalosporins - 4th Generation	55 (6.4%)
Antihistamines-Topical	55 (6.4%)
Antacids - Bicarbonate	54 (6.2%)
Bulk Laxatives	53 (6.1%)
Alpha-2 Receptor Antagonists (Tetracyclics)	52 (6.0%)
Ophthalmic Local Anesthetics	49 (5.7%)
Hemostatics - Systemic	49 (5.7%)
Zinc	48 (5.6%)
Dipeptidyl Peptidase-4 (DPP-4) Inhibitors	47 (5.4%)
Gallstone Solubilizing Agents	47 (5.4%)
Cycloplegic Mydriatics	47 (5.4%)
Protamine	58 (5.4%)
Butyrophenones	46 (5.3%)
Antidepressants - Misc.	45 (5.2%)
Mucolytics	45 (5.2%)
Leukotriene Modulators	44 (5.1%)
B-Complex Vitamins	44 (5.1%)
Acne Products	44 (5.1%)

Supplementary Table 9. Medications used in each pharmaceutical subclass

Pharmaceutical Subclass	Drug Name
ALKALINIZERS	BICITRA
ALKALINIZERS	CITRIC
ALKALINIZERS	CYTRA-2
ALKALINIZERS	CYTRA-3
ALKALINIZERS	POT
ALKALINIZERS	POTASSIUM
ANTI-INFECTIVES - THROAT	CLOTRIMAZOLE
ANTI-INFECTIVES - THROAT	MICONAZOLE
ANTI-INFECTIVES - THROAT	NYSTATIN
B-COMPLEX W/ FOLIC ACID	B
B-COMPLEX W/ FOLIC ACID	B-COMPLEX
B-COMPLEX W/ FOLIC ACID	DIALYVITE
B-COMPLEX W/ FOLIC ACID	FULL
B-COMPLEX W/ FOLIC ACID	NEPHRO-VITE
B-COMPLEX W/ FOLIC ACID	NEPHROCAPS
B-COMPLEX W/ FOLIC ACID	RENA-VITE
B-COMPLEX W/ FOLIC ACID	RENAL
B-COMPLEX W/ FOLIC ACID	RENAL-VITE
B-COMPLEX W/ FOLIC ACID	VOL-CARE
B-COMPLEX W/ FOLIC ACID	VP-VITE
BIGUANIDES	METFORMIN
CALCIUM CHANNEL BLOCKERS	ADALAT
CALCIUM CHANNEL BLOCKERS	AFEDITAB
CALCIUM CHANNEL BLOCKERS	AMLODIPINE
CALCIUM CHANNEL BLOCKERS	CARTIA
CALCIUM CHANNEL BLOCKERS	DILT-XR
CALCIUM CHANNEL BLOCKERS	DILTIAZEM
CALCIUM CHANNEL BLOCKERS	FELODIPINE

CALCIUM CHANNEL BLOCKERS	ISRADIPINE
CALCIUM CHANNEL BLOCKERS	NICARDIPINE
CALCIUM CHANNEL BLOCKERS	NIFEDICAL
CALCIUM CHANNEL BLOCKERS	NIFEDIPINE
CALCIUM CHANNEL BLOCKERS	NIMODIPINE
CALCIUM CHANNEL BLOCKERS	NORVASC
CALCIUM CHANNEL BLOCKERS	VERAPAMIL
CMV AGENTS	VALCYTE
CMV AGENTS	VALGANCICLOVIR
DIBENZAPINES	OLANZAPINE
DIBENZAPINES	QUETIAPINE
DIBENZAPINES	ZYPREXA
HEMATOPOIETIC GROWTH FACTORS	ARANESP
HEMATOPOIETIC GROWTH FACTORS	DARBEPOETIN
HEMATOPOIETIC GROWTH FACTORS	EPOETIN
HEMATOPOIETIC GROWTH FACTORS	EPOGEN
HEMATOPOIETIC GROWTH FACTORS	FILGRASTIM
HEMATOPOIETIC GROWTH FACTORS	FILGRASTIM-SNDZ
HEMATOPOIETIC GROWTH FACTORS	MIRCERA
HEMATOPOIETIC GROWTH FACTORS	NEULASTA
HEMATOPOIETIC GROWTH FACTORS	NEUPOGEN
HEMATOPOIETIC GROWTH FACTORS	PEGFILGRASTIM
HEMATOPOIETIC GROWTH FACTORS	PROCRIT
HEMATOPOIETIC GROWTH FACTORS	ROMIPLOSTIM
HEMATOPOIETIC GROWTH FACTORS	ZARXIO
IMMUNOSUPPRESSIVE AGENTS	ANTI-THYMOCYTE
IMMUNOSUPPRESSIVE AGENTS	AZATHIOPRINE
IMMUNOSUPPRESSIVE AGENTS	BASILIXIMAB
IMMUNOSUPPRESSIVE AGENTS	BELATACEPT
IMMUNOSUPPRESSIVE AGENTS	CELLCEPT

IMMUNOSUPPRESSIVE AGENTS	CYCLOSPORINE
IMMUNOSUPPRESSIVE AGENTS	EVEROLIMUS
IMMUNOSUPPRESSIVE AGENTS	IDS
IMMUNOSUPPRESSIVE AGENTS	MYCOPHENOLATE
IMMUNOSUPPRESSIVE AGENTS	MYCOPHENOLIC
IMMUNOSUPPRESSIVE AGENTS	MYFORTIC
IMMUNOSUPPRESSIVE AGENTS	NEORAL
IMMUNOSUPPRESSIVE AGENTS	PROGRAF
IMMUNOSUPPRESSIVE AGENTS	RAPAMUNE
IMMUNOSUPPRESSIVE AGENTS	SIROLIMUS
IMMUNOSUPPRESSIVE AGENTS	TACROLIMUS
METABOLIC MODIFIERS	CALCITRIOL
METABOLIC MODIFIERS	CINACALCET
METABOLIC MODIFIERS	DOXERCALCIFEROL
METABOLIC MODIFIERS	HECTOROL
METABOLIC MODIFIERS	PARICALCITOL
METABOLIC MODIFIERS	ROCALTROL
METABOLIC MODIFIERS	SENSIPAR
METABOLIC MODIFIERS	ZEMPLAR
OSMOTIC DIURETICS	MANNITOL
PHOSPHATE BINDER AGENTS	AURYXIA
PHOSPHATE BINDER AGENTS	CALCIUM
PHOSPHATE BINDER AGENTS	FERRIC
PHOSPHATE BINDER AGENTS	FOSRENOL
PHOSPHATE BINDER AGENTS	LANTHANUM
PHOSPHATE BINDER AGENTS	PHOSLO
PHOSPHATE BINDER AGENTS	RENAGEL
PHOSPHATE BINDER AGENTS	RENVELA
PHOSPHATE BINDER AGENTS	SEVELAMER
PHOSPHATE BINDER AGENTS	SUCROFERRIC

PHOSPHATE BINDER AGENTS	VELPHORO
POTASSIUM REMOVING RESINS	KALEXATE
POTASSIUM REMOVING RESINS	KAYEXALATE
POTASSIUM REMOVING RESINS	KIONEX
POTASSIUM REMOVING RESINS	PATIROMER
POTASSIUM REMOVING RESINS	SODIUM
POTASSIUM REMOVING RESINS	VELTASSA
SELECTIVE SEROTONIN REUPTAKE INHIBITORS (SSRIS)	CITALOPRAM
SELECTIVE SEROTONIN REUPTAKE INHIBITORS (SSRIS)	ESCITALOPRAM
SELECTIVE SEROTONIN REUPTAKE INHIBITORS (SSRIS)	FLUOXETINE
SELECTIVE SEROTONIN REUPTAKE INHIBITORS (SSRIS)	FLUVOXAMINE
SELECTIVE SEROTONIN REUPTAKE INHIBITORS (SSRIS)	LEXAPRO
SELECTIVE SEROTONIN REUPTAKE INHIBITORS (SSRIS)	PAROXETINE
SELECTIVE SEROTONIN REUPTAKE INHIBITORS (SSRIS)	SERTRALINE
SELECTIVE SEROTONIN REUPTAKE INHIBITORS (SSRIS)	ZOLOFT
SPECIALTY VITAMINS PRODUCTS	MG-PLUS
SPECIALTY VITAMINS PRODUCTS	ONE-A-DAY
SPECIALTY VITAMINS PRODUCTS	PROSTATE
SULFONYLUREAS	GLIMEPIRIDE
SULFONYLUREAS	GLIPIZIDE
SULFONYLUREAS	GLYBURIDE
THROMBOLYTIC ENZYMES	ALTEPLASE
VASODILATORS	HYDRALAZINE
VASODILATORS	MINOXIDIL
VASODILATORS	NITROPRUSSIDE

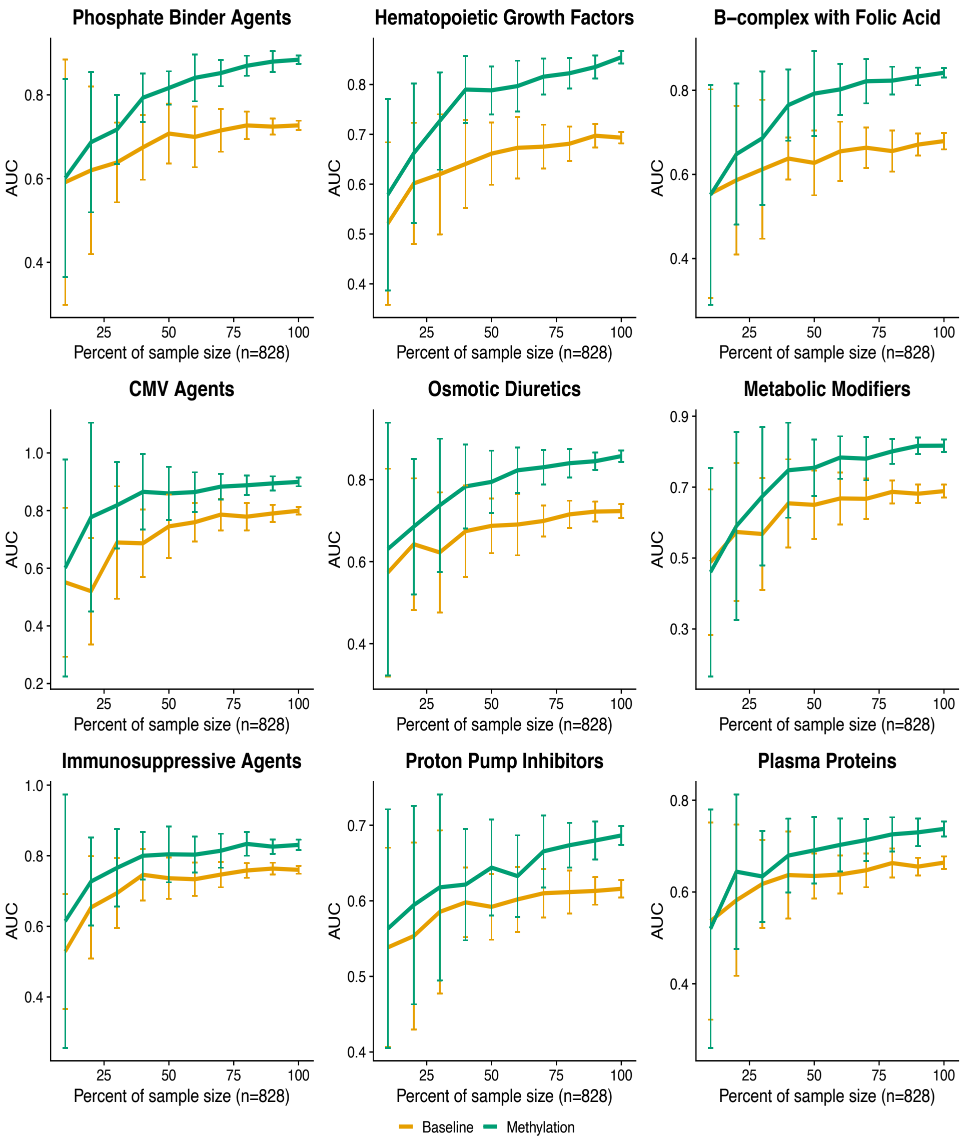

Supplementary Figure 6. Downsampled performance on additional medications We extended the downsampling experiments to include the top 10 most accurately imputed medications that also offered significant predictive power over the baseline features. We include here the remaining 9 medications. Error bars indicate 95% confidence intervals.

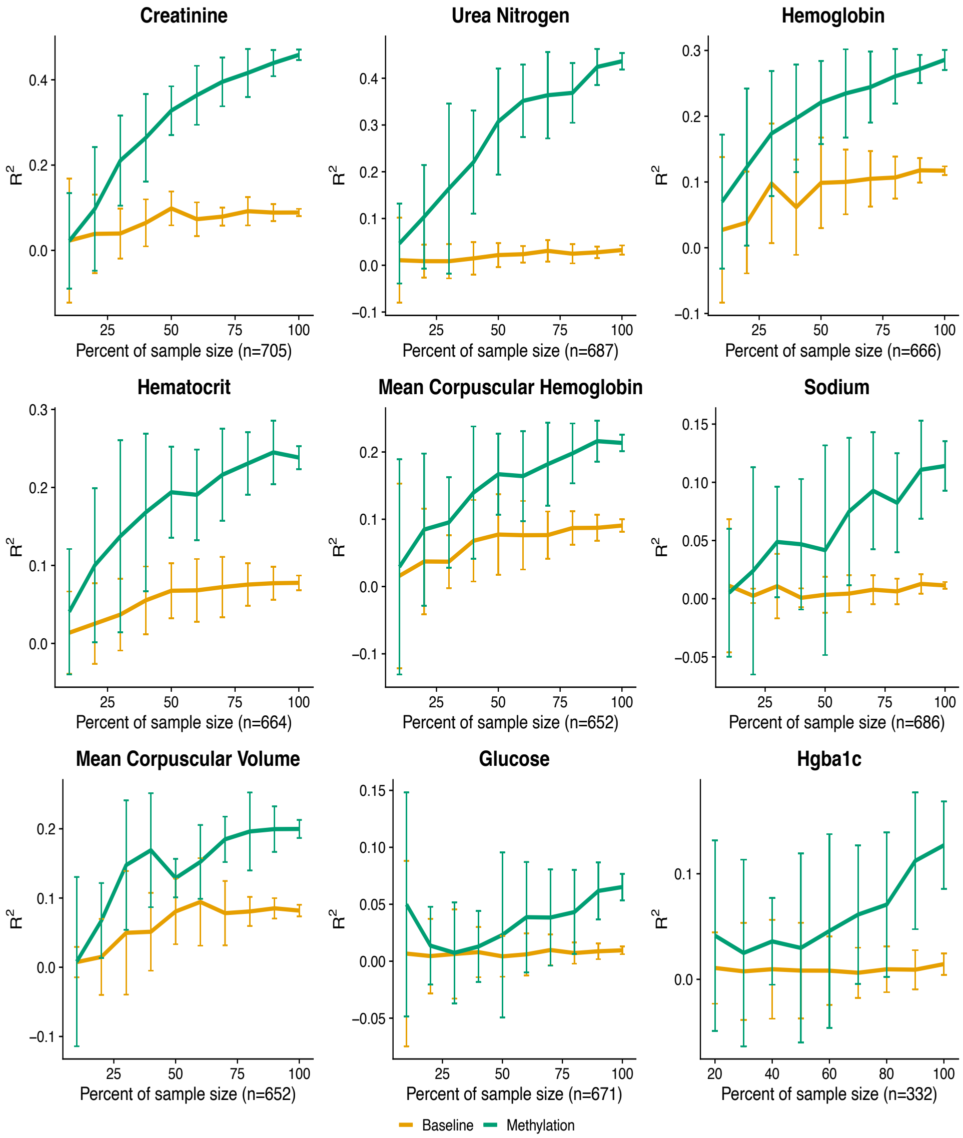

Supplementary Figure 7. Downsampled performance on additional labs We extended the downsampling experiments to include the top 10 most accurately imputed labs that also offered significant predictive power over the baseline features. We include here the remaining 9 labs. Error bars indicate 95% confidence intervals.

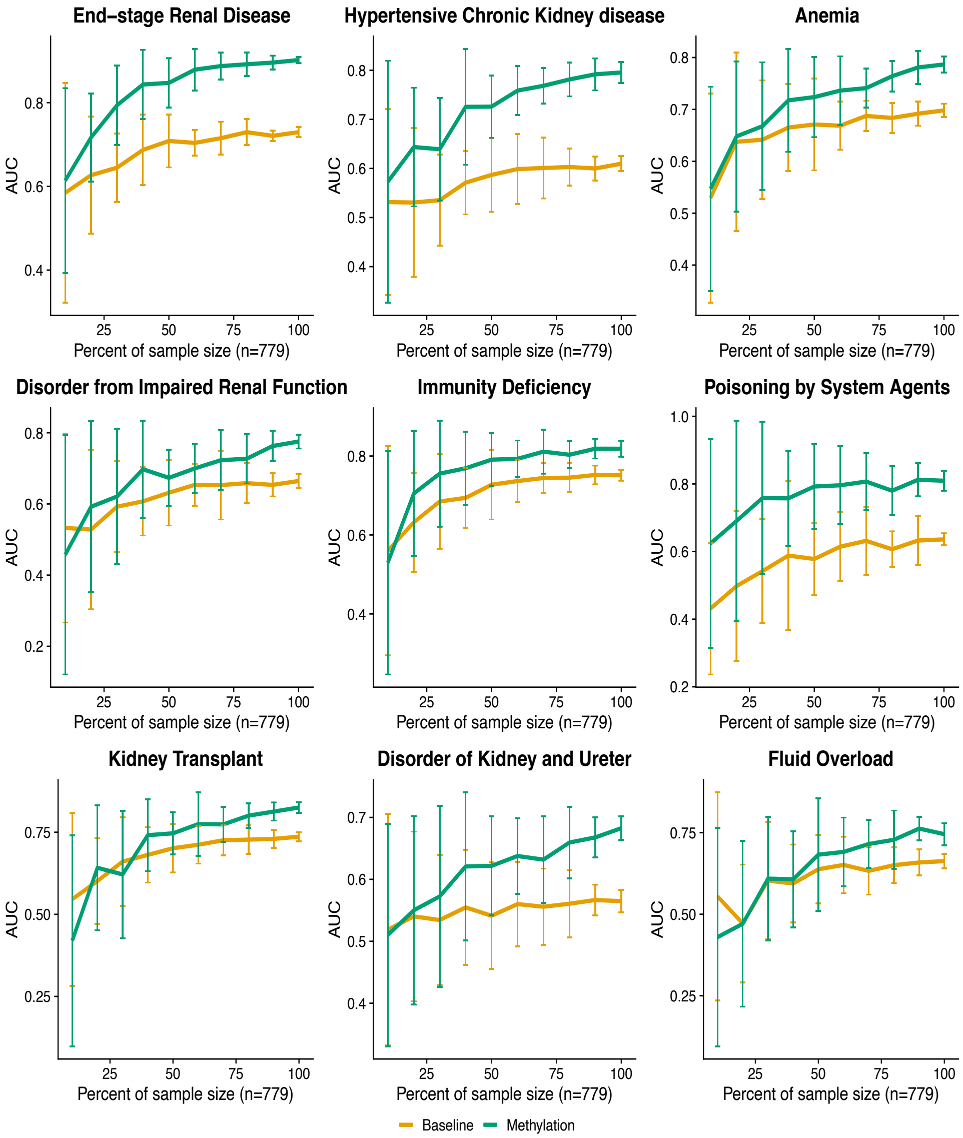

Supplementary Figure 8. Downsampled performance on additional Phecodes We extended the downsampling experiments to include the top 10 most accurately imputed Phecodes that also offered significant predictive power over the baseline features. We include here the remaining 9 Phecodes. Error bars indicate 95% confidence intervals.

Lab	PGS accession	Study	Number of variants in weight	Number of variants present in our data
Albumin	PGS000669	Sinnott-Armstrong et al.	11,912	9,172
Cholesterol	PGS000677	Sinnott-Armstrong et al.	17,204	13,401
Creatinine	PGS000679	Sinnott-Armstrong et al.	5,469	4,242
HGBA1C	PGS000685	Sinnott-Armstrong et al.	14,658	11,208
HDL	PGS000686	Sinnott-Armstrong et al.	25,070	19,123
Hematocrit	PGS001225	Tanigawa et al.	15,721	11,898
Hemoglobin	PGS001400	Tanigawa et al.	15,602	11,770
Mean corpuscular hemoglobin	PGS001219	Tanigawa et al.	13,003	9,853
Mean corpuscular volume	PGS001220	Tanigawa et al.	17,311	13,181
Urea nitrogen	PGS000701	Sinnott-Armstrong et al.	12,351	9,473

Supplementary Table 10. Polygenic scores used for the imputed genotypes We list below the weights used for computing the polygenic risk scores. We downloaded the weights from the Polygenic Score Catalogue (PGS)[54] from two studies of the UKBiobank [51, 53].

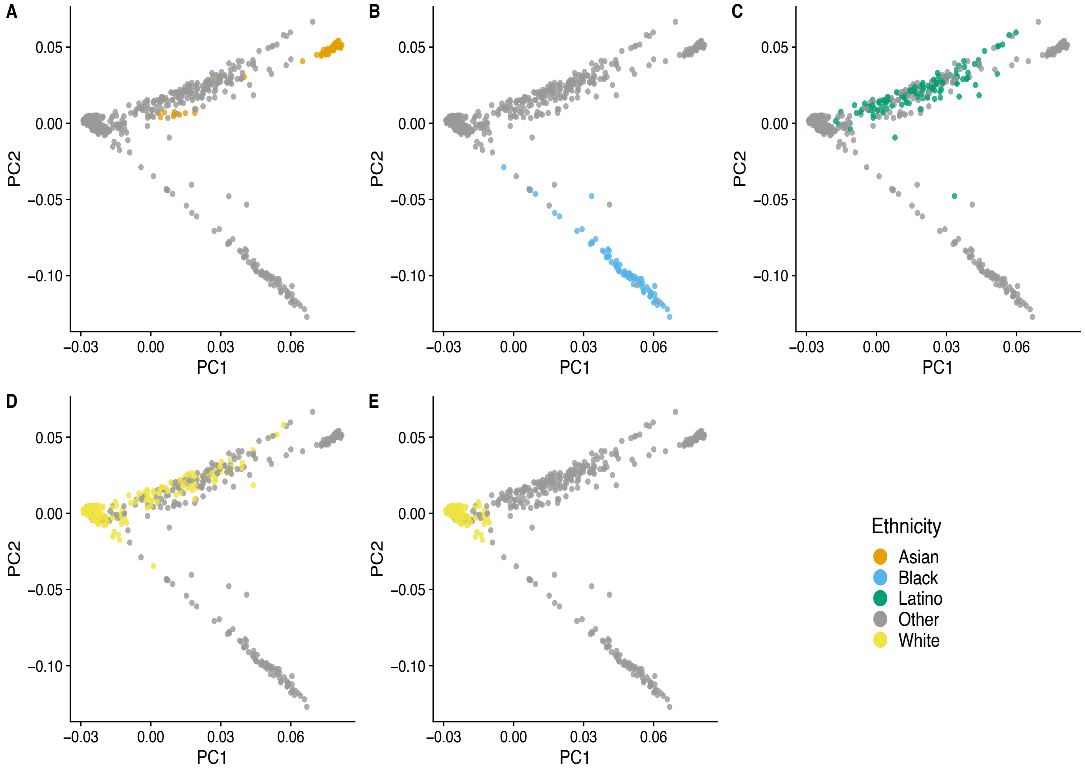

Supplementary Figure 9. Self-reported ancestry along genetic PCs We show the primary self-identified ethnicity in each plot individually. For the analysis using external PRS we limited the set of white-identifying individuals to those who additionally had a PC1 score of $- < .01$. We show the individuals used in our analysis in plot E.

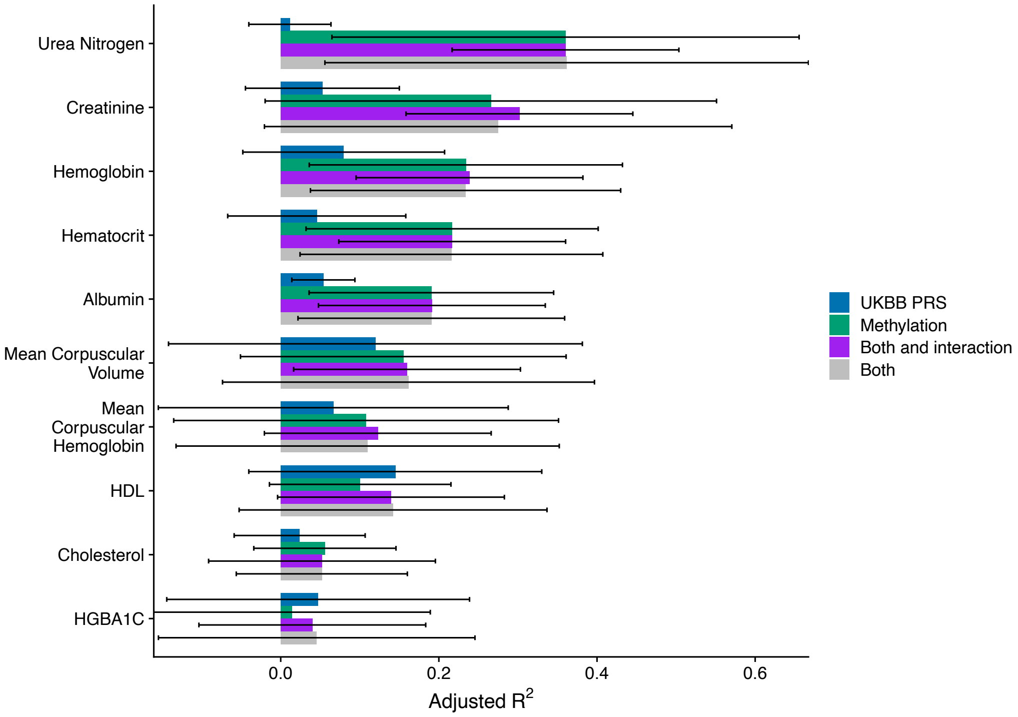

Supplementary Figure 10. Labs as predicted by methylation, an externally-trained polygenic risk score, both, and a model that includes both as well as their interaction The cross-validated adjusted R^2 between the true and imputed lab value on 541 unrelated patients of non-Hispanic-Latino white-identifying individuals using predictors that leveraged baseline features with either an MRS, a PRS externally-trained from the UKBiobank, both the MRS and the PRS, or a model that used both as well as the interaction between the MRS and PRS. Creatinine was the only outcome for which the interaction between both risk scores was statistically significant (p=9.16e-05), however the interaction for mean corpuscular hemoglobin was nominally significant (p=1.44e-02). HDL corresponds to high-density lipoprotein cholesterol and HGBA1C to glycated hemoglobin. Error bars indicate 95% confidence intervals.

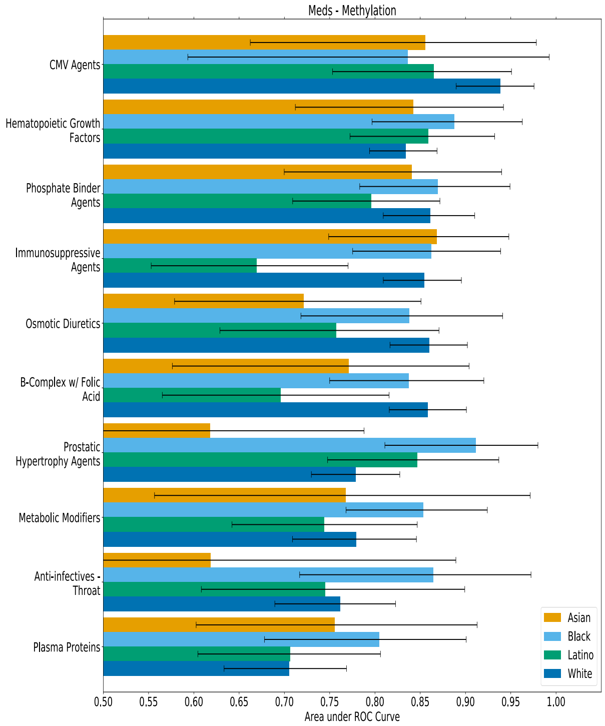

Supplementary Figure 11. Best methylation-predicted medications within ancestral populations After training a model on the entire heterogeneous set of individuals, we evaluated the predictive performance within each population separately. We observed no significant differences within self-reported ancestral groupings. Error bars indicate 95% confidence intervals.

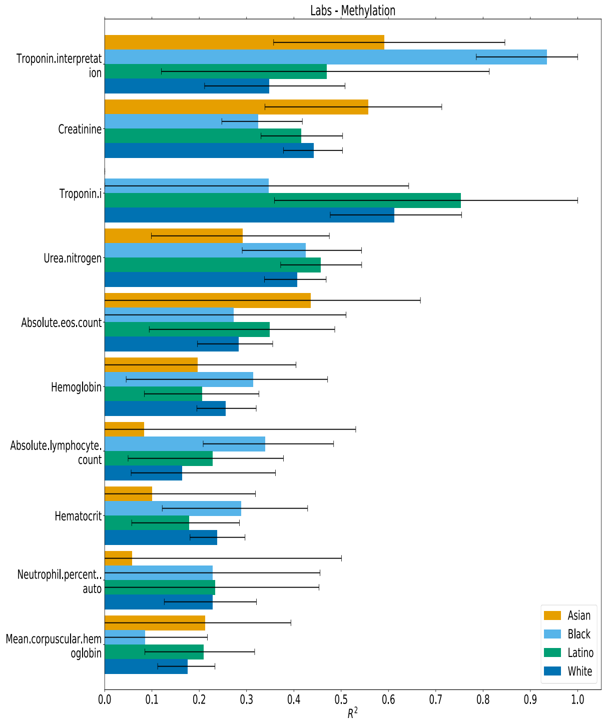

Supplementary Figure 12. Best methylation-predicted lab panels within ancestral populations After training a model on the entire heterogeneous set of individuals, we evaluated the predictive performance within each population separately. We observed no significant differences within self-reported ancestral groupings. Error bars indicate 95% confidence intervals.

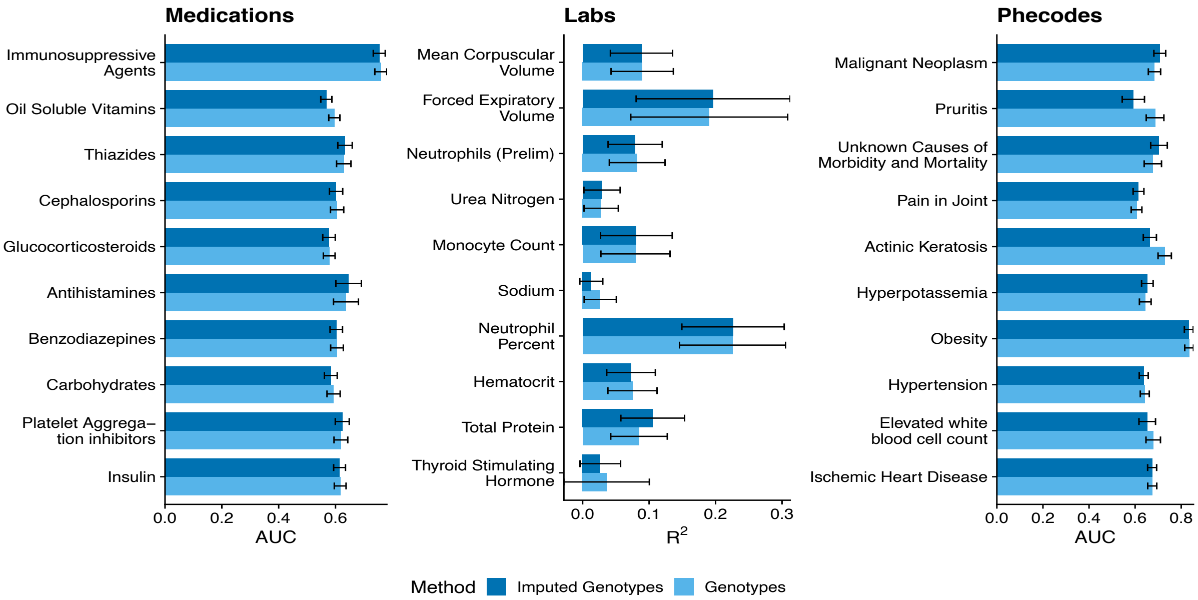

Supplementary Figure 13. Imputation accuracy when constructing PRS using chipped genotypes compared to using imputed genotypes We fit models using the imputed genotypes on the outcomes that were best imputed by the chipped genotypes and that significantly improved over the baseline model. Using the imputed genotypes did not result in significant differences in imputation accuracy when compared to the chipped genotypes. Error bars indicate 95% confidence intervals.

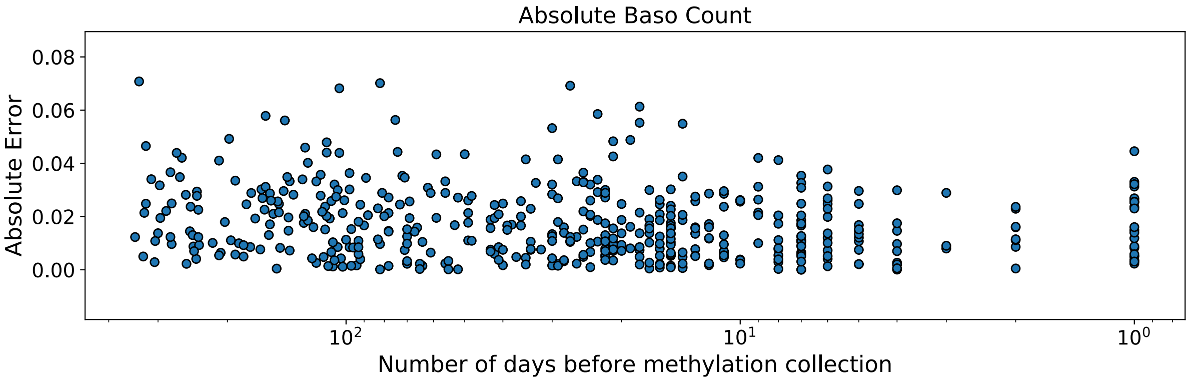

Supplementary Figure 14. Imputation error as a function of time since methylation sample collection date We analyzed the lab value imputation residuals to see if the errors were associated with the number of days between the lab result and the methylation collection date. After correcting for multiple hypotheses, only one lab showed a significant association between the imputed value residuals and time between collection dates (absolute basophil count, Pearson R=0.178, Bonferroni corrected p-value=0.0045). Here we show the absolute residual error as a function of the number of days the lab resulted before methylation collection for the only significantly associated lab, absolute basophil count (note the log scaling on the x-axis)
