# Supplementary material for: Methylation risk scores are associated with a collection of phenotypes within electronic health record systems
Source: NPJ Genom Med. 2022 Aug 25;7:50. doi: 10.1038/s41525-022-00320-1 (PMC9411568; doi:10.1038/s41525-022-00320-1)
Supplement: Supplementary file 2 — Reporting Summary [file 41525_2022_320_MOESM2_ESM.pdf]

nature portfolio

Corresponding author(s): Eran Halperin, Mike Thompson

Last updated by author(s): Feb 10, 2022

Reporting Summary

Nature Portfolio wishes to improve the reproducibility of the work that we publish. This form provides structure for consistency and transparency in reporting. For further information on Nature Portfolio policies, see our Editorial Policies and the Editorial Policy Checklist.

Statistics

For all statistical analyses, confirm that the following items are present in the figure legend, table legend, main text, or Methods section.

n/a	Confirmed	
☐	☒	The exact sample size (n) for each experimental group/condition, given as a discrete number and unit of measurement
☐	☐	A statement on whether measurements were taken from distinct samples or whether the same sample was measured repeatedly
☐	☐	The statistical test(s) used AND whether they are one- or two-sided<br>*Only common tests should be described solely by name; describe more complex techniques in the Methods section.*
☐	☒	A description of all covariates tested
☐	☒	A description of any assumptions or corrections, such as tests of normality and adjustment for multiple comparisons
☐	☒	A full description of the statistical parameters including central tendency (e.g. means) or other basic estimates (e.g. regression coefficient) AND variation (e.g. standard deviation) or associated estimates of uncertainty (e.g. confidence intervals)
☐	☒	For null hypothesis testing, the test statistic (e.g. F, t, r) with confidence intervals, effect sizes, degrees of freedom and P value noted<br>*Give P values as exact values whenever suitable.*
☒	☐	For Bayesian analysis, information on the choice of priors and Markov chain Monte Carlo settings
☒	☐	For hierarchical and complex designs, identification of the appropriate level for tests and full reporting of outcomes
☒	☐	Estimates of effect sizes (e.g. Cohen's d, Pearson's r), indicating how they were calculated

Our web collection on statistics for biologists contains articles on many of the points above.

Software and code

Policy information about availability of computer code

Data collection	De-identified electronic health record data for this study was extracted from the perioperative data warehouse (PDW), a custom-built, robust data warehouse containing all patients who have undergone surgery at UCLA Health since the implementation of UCLA's EMR (EPIC Systems, Madison, WI, USA) in March 2013. The PDW, which has been described previously[66], has a two-stage design. First, data are extracted from EPIC's Clarity database into 29 tables organised around three distinct concepts: patients, surgical procedures, and health system encounters. Then, these data are used to populate a series of 4000 distinct measures and metrics such as procedure duration, admission ICD codes, lab results, and medication orders.
Data analysis	We proprocessed genotype data using bigstatsr's implementation of Beagle (d20), Plink 1.07 and 1.9, and GCTA 1.93.2. Genotype imputation was performed on the Michigan Imputation Server with Eagle v2.4 with Freeze5 imputation panel and minimac4. Methylation arrays were preprocessed using ENmix 1.22.0. softImpute was run with package fancyimpute 0.5.5.

For manuscripts utilizing custom algorithms or software that are central to the research but not yet described in published literature, software must be made available to editors and reviewers. We strongly encourage code deposition in a community repository (e.g. GitHub). See the Nature Portfolio guidelines for submitting code & software for further information.

Data

Policy information about availability of data

All manuscripts must include a data availability statement. This statement should provide the following information, where applicable:

- Accession codes, unique identifiers, or web links for publicly available datasets
- A description of any restrictions on data availability
- For clinical datasets or third party data, please ensure that the statement adheres to our policy

De-identified patient medical records along with genomic data are not authorized for public sharing.

Field-specific reporting

Please select the one below that is the best fit for your research. If you are not sure, read the appropriate sections before making your selection.

☒ Life sciences ☐ Behavioural & social sciences ☐ Ecological, evolutionary & environmental sciences

For a reference copy of the document with all sections, see nature.com/documents/nr-reporting-summary-flat.pdf

Life sciences study design

All studies must disclose on these points even when the disclosure is negative.

Sample size	833 samples were ascertained on whether they had measured genotypes from a previous study as well as whether they had kidney injury or heart attack. Controls were matched using propensity scoring. No power analyses were conducted prior to sample collection.
Data exclusions	*Describe any data exclusions. If no data were excluded from the analyses, state so OR if data were excluded, describe the exclusions and the rationale behind them, indicating whether exclusion criteria were pre-established.*
Replication	We trained models for kidney disease (and associated phenotypes) as well as medication intake for Schizophrenia and Rheumatoid Arthritis patients. We replicated our imputations across all three outcomes.
Randomization	Individuals were randomized across methylation array plates.
Blinding	*Describe whether the investigators were blinded to group allocation during data collection and/or analysis. If blinding was not possible, describe why OR explain why blinding was not relevant to your study.*

Reporting for specific materials, systems and methods

We require information from authors about some types of materials, experimental systems and methods used in many studies. Here, indicate whether each material, system or method listed is relevant to your study. If you are not sure if a list item applies to your research, read the appropriate section before selecting a response.

Materials & experimental systems

n/a	Involved in the study	
☒	☐	Antibodies
☒	☐	Eukaryotic cell lines
☒	☐	Palaeontology and archaeology
☒	☐	Animals and other organisms
☐	☒	Human research participants
☒	☐	Clinical data
☒	☐	Dual use research of concern

Methods

n/a	Involved in the study	
☒	☐	ChIP-seq
☒	☐	Flow cytometry
☒	☐	MRI-based neuroimaging

Human research participants

Policy information about studies involving human research participants

Population characteristics	Patients spanned self-identified race/ethnicity categories of Asian, Black, Latino, and White. Patients ranged from age 18 to 90 and included both male and female sex. See ascertainment above for disease characteristics .
Recruitment	833 samples were ascertained on whether they had measured genotypes from a previous study as well as whether they had kidney injury or heart attack. Controls were matched using propensity scoring. The cohort is comprised of self-identified Asian, Black, Latino, and White individuals. Individuals who had consented to genotyping were selected for methylation assaying based on the ascertainment procedure described above.

Ethics oversight

Patient Recruitment and Sample Collection for Precision Health Activities at UCLA is an approved study by the UCLA Institutional Review Board (UCLA IRB). IRB#17-001013

Note that full information on the approval of the study protocol must also be provided in the manuscript.
